# Binding of inhibitors to the monomeric and dimeric SARS-CoV-2 Mpro†

**DOI:** 10.1039/d0ra09858b

**Published:** 2021-01-13

**Authors:** Nguyen Minh Tam, Pham Cam Nam, Duong Tuan Quang, Nguyen Thanh Tung, Van V. Vu, Son Tung Ngo

**Affiliations:** Computational Chemistry Research Group, Ton Duc Thang University Ho Chi Minh City Vietnam; Faculty of Applied Sciences, Ton Duc Thang University Ho Chi Minh City Vietnam; Department of Chemistry, The University of Danang, University of Science and Technology Danang Vietnam; University of Education, Hue University Vietnam; Institute of Materials Science, Vietnam Academy of Science and Technology Hanoi Vietnam; Graduate University of Science and Technology, Vietnam Academy of Science and Technology Hanoi Vietnam; NTT Hi-Tech Institute, Nguyen Tat Thanh University Ho Chi Minh City Vietnam; Laboratory of Theoretical and Computational Biophysics, Ton Duc Thang University Ho Chi Minh City Vietnam ngosontung@tdtu.edu.vn

## Abstract

SARS-CoV-2 rapidly infects millions of people worldwide since December 2019. There is still no effective treatment for the virus, resulting in the death of more than one million patients. Inhibiting the activity of SARS-CoV-2 main protease (Mpro), 3C-like protease (3CLP), is able to block the viral replication and proliferation. In this context, our study has revealed that *in silico* screening for inhibitors of SARS-CoV-2 Mpro can be reliably done using the monomeric structure of the Mpro instead of the dimeric one. Docking and fast pulling of ligand (FPL) simulations for both monomeric and dimeric forms correlate well with the corresponding experimental binding affinity data of 24 compounds. The obtained results were also confirmed *via* binding pose and noncovalent contact analyses. Our study results show that it is possible to speed up computer-aided drug design for SARS-CoV-2 Mpro by focusing on the monomeric form instead of the larger dimeric one.

## Abstract 2

Binding of inhibitors to the monomeric SARS-CoV-2 Mpro is similar to the dimeric one.

## Graphical abstract

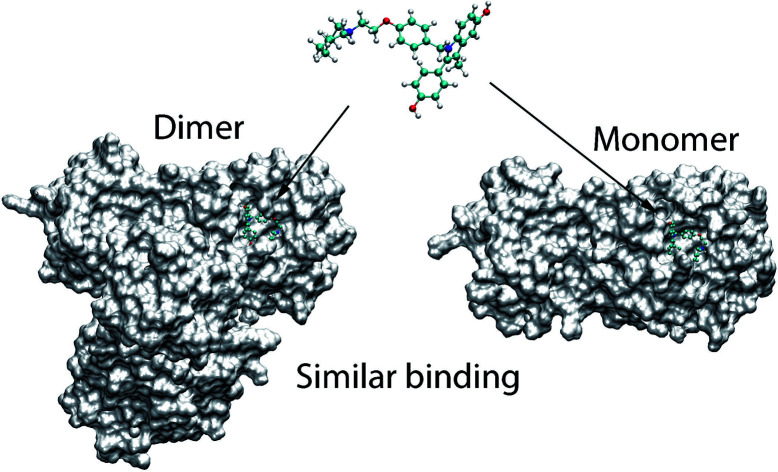

## Introduction

The novel coronavirus (2019-nCoV or SARS-CoV-2), a member of the Coronaviridae virus family, has been reported to be able to spread among humans.^1^ The virus initially appeared in December 2019 in Wuhan, Hubei Province, China.^2–4^ It shares more than 82% identical RNA genome to the SARS-CoV, SARS-CoV-2 severe cases of respiratory syndromes.^5^ Although the bat has been thought of as the original reservoir, the intermediate host is still unknown.^6^ Moreover, it is known that the SARS-CoV-2 can endure in aerosol for more than 3 hours,^7^ which may be a major factor behind the outbreak of COVID-19 pandemic, which has caused several hundred thousands of deaths worldwide.^5^ Therefore, the COVID-19 pandemic becomes an urgency for community health, which requires to develop an effective treatment or vaccine immediately.

Coronaviruses genomes occupy *ca.* 26–32 kb in length which is the largest sequence among RNA viruses.^8,9^ The SARS-CoV-2 genome encodes more than 20 various structural and non-structural proteins. Particularly, the SARS-CoV-2 main protease (Mpro), 3C-like protease (3CLP), is one of the most important viral enzymes, having more than 96% similarity with SARS-CoV 3CLP.^9,10^ SARS-CoV-2 Mpro cleaves nascent polyproteins, which are produced by the translation of the viral RNA. During this process, 11 non-structural polyproteins are auto-cleaved to become polypeptides, which are required for the viral replication and transcription.^9^ Therefore, SARS-CoV-2 Mpro turns out to be an attractive target for antiviral drug development since blocking viral protease can inhibit viral replication and proliferation.^10,11^ Numerous investigations following this strategy have been carried out and shown some initial success.^12–21^ However, unfortunately, an effective drug for COVID-19 is still unavailable.

Currently, it should be noted that the time and cost to advance a drug has been significantly decreased by using the power of computational approaches.^22–25^ Generally, the binding free energy, Δ*G*, between a ligand and an enzyme can be probed *via* computational approaches. The Δ*G* is associated with the experimental inhibition constant, *k*_i_, *via* formula Δ*G*_bind_ = *RT* ln(*k*_i_), where *R* is gas constant, *T* is absolute temperature, and *k*_i_ is a critical metric revealing the nature of binding between two biomolecules.^22^ Accurate assessment of the ligand-binding free energy is very important in computer-aided drug design (CADD) problem.^26^

In addition, it should be noted that the dimer was shown to be the biologically active form of the SARS-CoV Mpro instead of the monomeric one.^27^ Moreover, the SARS-CoV-2 Mpro possibly acts like the SARS-CoV Mpro due to the dissimilitude of only is 12 of 306 amino acids. However, fortunately, the interface of SARS-CoV-2 Mpro does not contain a ligand-binding pocket,^14^ the computational screening potential inhibitors for SARS-CoV-2 Mpro are thus possible to carry out based on the monoclinic structure.^28,29^ However, an important question is raised that what is the difference when we use the monomeric form of SARS-CoV-2 Mpro as inhibitor-screening target instead of the dimeric one to reduce CPU time consumption? In this context, the binding affinity of available inhibitors^12–21^ to the monomeric and dimeric SARS-CoV-2 Mpro was examined *via* docking and FPL schemes. The affinity of the some inhibitors of SARS-CoV-2 to the Mpro was also evaluated. The high correlation coefficients between computational values of monomeric and dimeric systems suggests that we can use the monomeric form of SARS-CoV-2 Mpro as CADD target instead of the dimeric form. Moreover, the similar of Pearson correlation between computed and experimental metrics of SARS-CoV-2 Mpro monomer and dimer was observed. The obtained results can be beneficial to the COVID-19 therapy by speeding up CADD progression.

## Materials and methods

### Structure of inhibitors and SARS-CoV-2 Mpro

Three-dimensional structures of the monomeric and dimeric SARS-COV-2 Mpro were downloaded from the Protein Data Bank with ID 6Y2F^14^ and 6XBG,^30^ respectively. The protonation states of the protease at pH = 7.0 was computed using H++ server.^31^ Inhibitor structures were taken from the PubChem database.^32^ The ligand protonation state were assessed at pH = 7.0 by using a webserver, http://www.chemicalize.com, which is a tool of ChemAxon. The ligand structure was first optimized using quantum mechanics (QM) simulation with the B3LYP functional at 6-31G(d) level of basis set.

### Molecular docking simulations

**Fig. 1:**
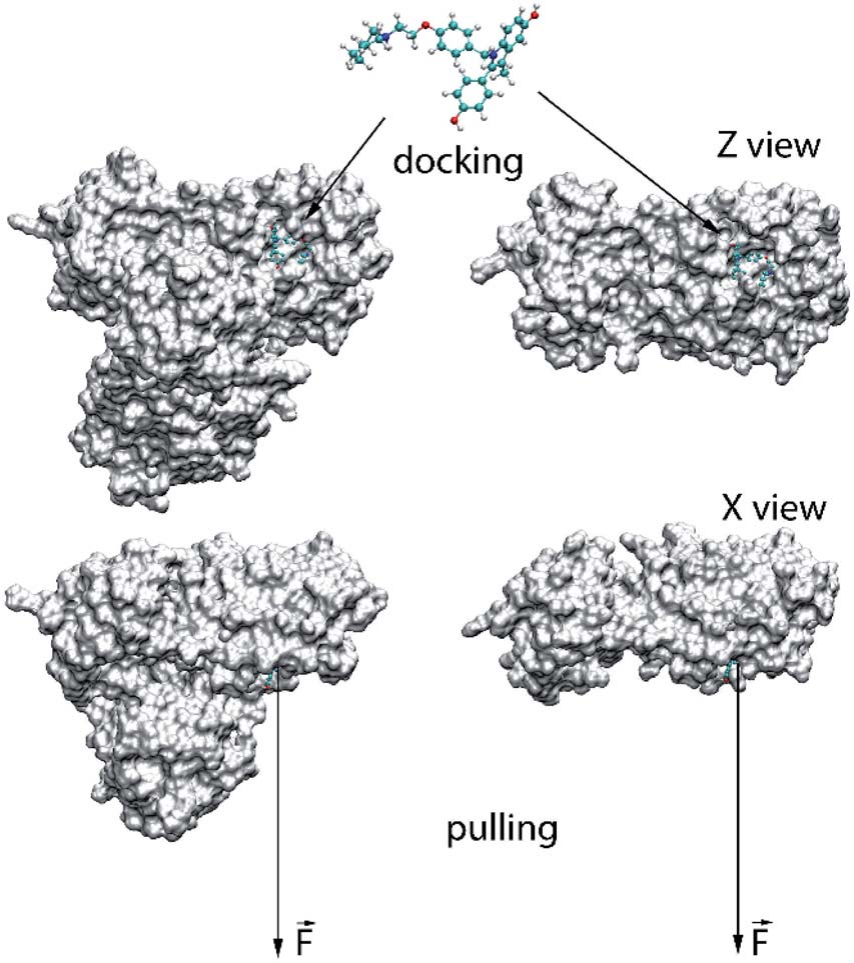
Computational scheme for evaluation of the ligand-binding affinity to the monomeric and dimeric SARS-CoV-2 Mpro.

The binding position and affinity of ligands to the monomeric and dimeric SARS-CoV-2 Mpro were probed *via* the Autodock Vina package (*cf.*Fig. 1).^33^ In particular, the AutodockTool 1.5.6 was manipulated to topologize the ligands and receptors.^34^ The docking parameter was selected referring to the previous study,^35–37^ in which the exhaustiveness is of 8. The obtained-docking result was chosen as the highest binding affinity conformations. The grid center was selected as the geometric center of the α-ketoamide 13b and UAW246 compounds, which correspond to the monomeric and dimeric Mpro, respectively.^14,30^ The grid size was chosen as 24 × 24 × 24 Å, which entirely cover the ligand-binding cleft of the Mpro.^36,37^

### Steered-molecular dynamics simulations

GROMACS version 5.1.3 (ref. 38) was used to simulate the solvated complex involving the ligand and monomeric/dimeric SARS-COV-2 Mpro. The protease and inhibitor were topologized *via* the Amber99SB-ILDN^39^ and general Amber force field (GAFF),^40^ correspondingly. It should be recorded that the ligand parameterization was completed by using AmberTools18 and ACPYPE approaches.^41,42^ In particular, the inhibitor atomic charges were assigned *via* the Restrained Electrostatic Potential (RESP) method^40^ through QM investigation at the level of B3LYP/6-31G(d,p). During QM simulation, the implicit water model, *ε* = 78.4, was involved. The monomeric and dimeric SARS-CoV-2 Mpro + inhibitor were inserted into a rectangular periodic boundary condition (rPBC) box with a dimension of (9.8, 5.9, 8.7) and (9.4, 9.0, 12.1) nm, respectively. The corresponding box volumes of the monomeric and dimeric systems are 506.28 and 1013.82 nm^3^, respectively. Therefore, the total atoms of these systems approximately are 50 000 and 100 000 atoms, respectively.

The atomistic simulation was performed utilizing the parameters referred to the prior works.^36,37^ Particularly, the MD time step is 2 fs. The noncovalent pair was affected within a radius of 0.9 nm. The electrostatics interaction was assessed implementing the fast particle-mesh Ewald electrostatics scheme.^43^ The SARS-CoV-2 + inhibitor was then optimized and equalized throughout the EM, NVT, and NPT imitations. The NVT and NPT imitations were operated during intervals of 0.1 and 2.0 ns, correspondingly. Moreover, the SARS-CoV-2 Mpro Cα atoms were restrained during these imitations *via* a small harmonic force with a value of 1000 kJ mol^−1^ nm^−2^ per proportions. The relaxed conformation of the SARS-CoV-2 Mpro + inhibitor was then employed as initial structure of FPL simulation. During which, the inhibitor was pulled out of the binding cleft under effect of an externally harmonic force with parameters of *k* = 0.005 nm ps^−1^ and *v* = 600 kJ mol^−1^ nm^−2^ for pulling speed and cantilever spring constant (*cf.*Fig. 1), respectively.^37^ Totally, 8 independent trajectories were carried out to assess the ligand-binding affinity.

### Analyzed tools

An intermolecular nonbonded contact was enumerated when the minimum distance of nonhydrogen atoms of a residue to the inhibitor was smaller than 0.45 nm. A hydrogen bond was enumerated when the angle between donor, D, hydrogen, H, acceptor, A, is larger than 135° and the distance between D and A is smaller than 0.35 nm. The error of computations was computed through 1000 rounds of the bootstrapping method.^44^

## Results and discussion

### Docking calculations

**Fig. 2:**
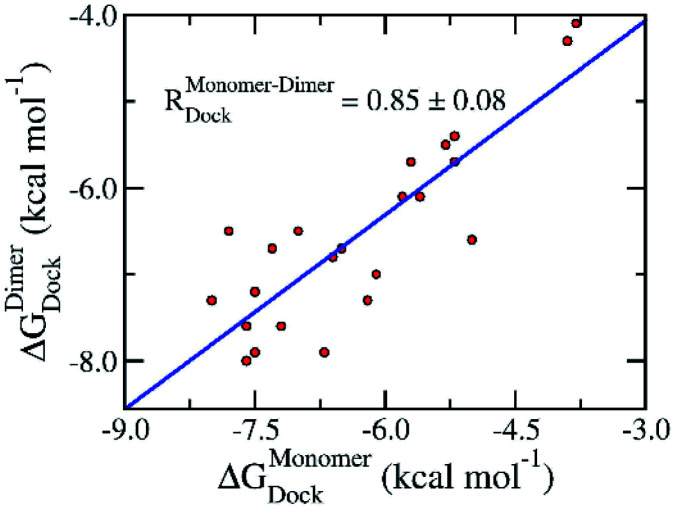
Correlation between docking results of ligands to monomeric and dimeric forms of SARS-CoV-2 Mpro. Computational results were obtained using Autodock Vina. The computed error was attained *via* 1000 rounds of the bootstrapping method.^44^

The preliminary investigations of binding pose and affinity of the trial inhibitors to the monomeric and dimeric SARS-CoV-2 Mpro were initially estimated by a molecular docking method. Autodock Vina,^33^ a very efficient molecular docking approach with a successful-docking rate up to 81%,^35^ would be able to complete this task. We have thus docked 24 available inhibitors to the monomeric and dimeric SARS-CoV-2 Mpro using Autodock Vina referring to the previous study.^36,37^ Moreover, the successful-docking rate of Autodock Vina on the SARS-CoV-2 Mpro is also appropriate due to the recent report with the number is 67%.^45^ By using exhaustiveness 8 as suggested in the previous work,^35^ the results were rapidly obtained in few hours (Tables 1 and S1 of the ESI file†). Interestingly, in good agreement with the previous studies,^45^ Autodock Vina adopts an appropriate results compared with the experimental affinities, in which, the correlation coefficient of the monomeric target, *R*^Monomer^_Dock_ = 0.50 ± 0.15, is similar to that of the dimeric target, *R*^Dimer^_Dock_ = 0.50 ± 0.12. It should be noted that the obtained correlation is in good agreement with the previous benchmark of Autodock Vina over 800 various complexes, *R*^exhaustiveness^_Vina_ = 8 = 0.489 ± 0.027.^35^ Moreover, the root mean square error (RMSE) between calculated and experimental values are small indicating the accurate result, in which, the monomeric system gives a value of RMSE^Monomer^_Dock_ = 1.89 ± 0.15 kcal mol^−1^ and the dimeric system adopts a metric of RMSE^Dimer^_Dock_ = 1.65 ± 0.17 kcal mol^−1^. Furthermore, comparing the docking results of ligands to monomeric and dimeric Mpro would reveal the correlation of using monomeric and dimeric systems as docking target. The obtained results indicate that the docking of ligands to monomeric Mpro well correlates with the corresponding values of the dimeric system, *R*^Monomer–Dimer^_Dock_ = 0.85 ± 0.08 (Fig. 2). The accuracy of docking results in different targets is also high due to the small value of the obtained RMSE, RMSE^Monomer–Dimer^_Dock_ = 0.66 ± 0.10 kcal mol^−1^. It may thus argue that we are able to use the monomeric form of SARS-CoV-2 Mpro instead of the dimeric form for screening potential inhibitors of the Mpro *via* the docking approach. It should be noted that the computed error bars was obtained *via* 1000 rounds of the bootstrapping method.^44^

**Table 1:** Computed values of docking energy in comparison with experiments

No.	Name	Δ*G*^Monomer^_Dock_			Δ*G*^Dimer^_Dock_			Δ*G*_EXP_^a^
		Short	Medium	Long	Short	Medium	Long	
1	7j	−7.2	−7.4	−7.2	−7.6	−7.4	−7.3	−8.69
2	11a	−7.5	−7.6	−7.6	−7.2	−7.1	−7.1	−9.96
3	11b	−8.0	−8.1	−8.0	−7.3	−7.4	−7.4	−10.13
4	11r	−6.7	−6.4	−6.3	−7.9	−8.1	−8.3	−9.23
5	13a	−7.6	−7.6	−7.6	−8.0	−7.8	−7.8	−7.70
6	13b	−7.6	−7.8	−7.8	−7.6	−7.1	−7.8	−8.45
7	Calpain inhibitor I	−5.2	−5.2	−5.2	−5.4	−5.4	−5.6	−6.94
8	Calpain inhibitor II	−5.3	−5.5	−5.5	−5.5	−5.7	−5.6	−8.23
9	Calpain inhibitor XII	−6.2	−6.3	−6.3	−7.3	−7.3	−7.2	−8.69
10	Calpeptin	−5.8	−5.5	−6.1	−6.1	−6.4	−6.3	−6.81
11	Candesartan cilexetil	−7.5	−7.4	−7.9	−7.9	−8.4	−8.4	−7.60
12	Carmofur	−5.2	−5.5	−5.6	−5.7	−5.8	−6.1	−7.86
13	Chloroquine	−5.0	−5.3	−5.1	−6.6	−6.6	−6.6	−7.41
14	Dipyridamole	−6.5	−6.5	−6.6	−6.7	−6.6	−6.6	−8.52
15	Disulfiram	−3.9	−3.8	−3.9	−4.3	−4.1	−4.1	−6.89
16	GC-373	−7.0	−7.0	−7.1	−6.5	−6.8	−7.0	−8.76
17	Hydroxychloroquine	−5.8	−6.3	−6.2	−6.1	−6.2	−6.5	−7.58
18	MG-115	−5.7	−5.7	−5.5	−5.7	−5.7	−6.1	−7.53
19	MG-132	−5.6	−6.2	−6.2	−6.1	−5.8	−6.2	−7.41
20	Narlaprevir	−7.8	−7.5	−7.4	−6.5	−6.9	−6.8	−7.18
21	Omeprazole	−6.6	−6.6	−6.6	−6.8	−6.8	−6.8	−6.40
22	Oxytetracycline	−7.3	−7.3	−7.3	−6.7	−6.7	−6.7	−6.60
23	PX-12	−3.8	−3.8	−3.8	−4.1	−4.2	−4.5	−6.39
24	Shikonin	−6.1	−6.1	−6.1	−7.0	−6.9	−6.9	−6.58

a The experimental binding free energies were gained based on IC_50_ value,^12–18^ approximating that the one equals to the inhibition constant *k*_i_. The unit is of kcal mol^−1^.

**Fig. 3:**
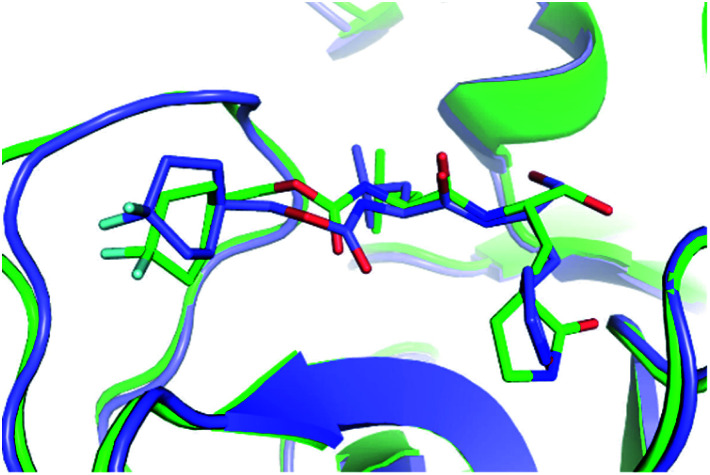
The superposition of the compound 7j in the binding mode with the monomeric and dimeric SARS-CoV-2 Mpro. In particular, the green and slate colors mentioned the monomeric and dimeric complexes, correspondingly.

Furthermore, the binding pose of inhibitors to the monomeric and dimeric SARS-CoV-2 Mpro is in good agreement together since espousing the root-mean-square deviation (RMSD) of 0.21 ± 0.02 nm (*cf.*Fig. 3 and Table S1 of the ESI†). It should be noted that the RMSD of the ligand-binding poses, which is smaller than 0.20 nm, normally counted as the conformations locating in the same cluster.^35,46^ The difference in docking poses probably causes the uncorrelation between docking energies of monomeric and dimeric systems. The structural observation is thus confirmed the obtained docking energy above.

**Fig. 4:**
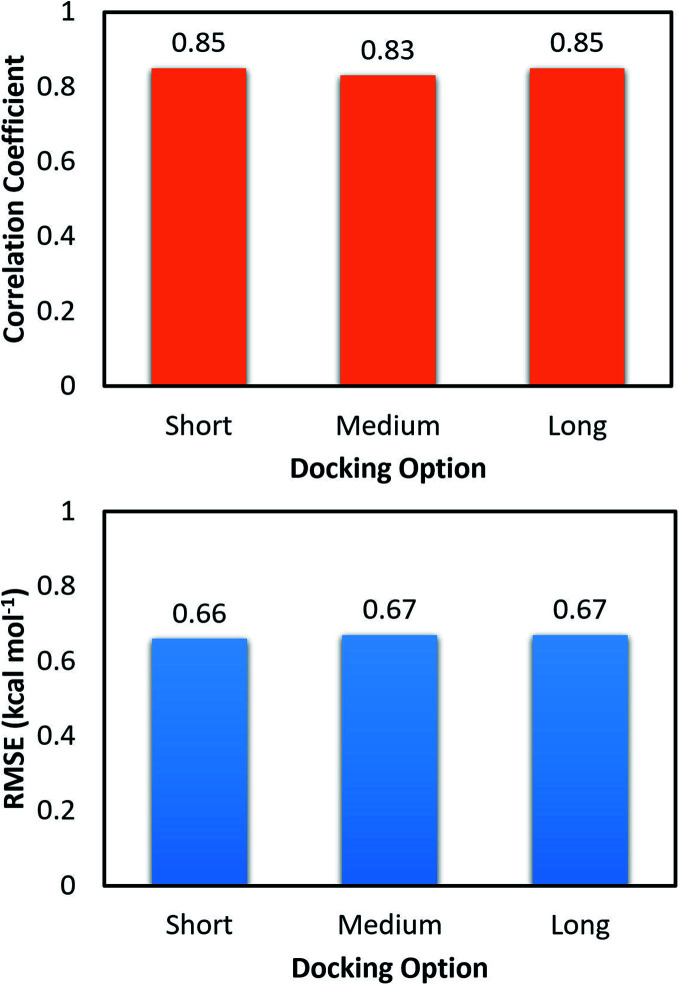
Correlation and RMSE values between docking binding affinity of ligands to monomeric and dimeric forms of SARS-CoV-2 Mpro.

The molecular docking with larger exhaustiveness, which selected as 56 and 400 according to the previous study,^35^ were also performed in order to validate the convergence of the docking scheme. In total we used three different values of exhaustiveness including 400, 56, and 8 which are denoted as *long*, *medium*, and *short* options, respectively. The associations of the docking simulations for monomer and dimer with respect to experiment are shown in Table 1. Interestingly, changing the docking exhaustiveness parameter from *short* to *medium* and/or *long* does not have a significant impact on the correlation coefficient and RMSE, which is consistent with the prior benchmark.^35^ In particular, the correlation coefficients slightly change to *R*^Monomer^_Dock_ = 0.53 ± 0.14 and *R*^Dimer^_Dock_ = 0.49 ± 0.13 matching with the *medium* option. The metrics are of *R*^Monomer^_Dock_ = 0.50 ± 0.15 and *R*^Dimer^_Dock_ = 0.50 ± 0.12 resembling the *long* option. Moreover, the calculated accuracy is also associated with the RMSE value. Absolutely, within computed error, the RMSE was unchanged over the docking options *short*, *medium*, and *long* with amounts of RMSE^Dimer^_Dock_ = 1.65 ± 0.17, RMSE^Dimer^_Dock_ = 1.65 ± 0.17, and RMSE^Dimer^_Dock_ = 1.55 ± 0.18 kcal mol^−1^ for the dimeric system and RMSE^Monomer^_Dock_ = 1.89 ± 0.15, RMSE^Monomer^_Dock_ = 1.81 ± 0.17, and RMSE^Monomer^_Dock_ = 1.81 ± 0.16 kcal mol^−1^ for the monomeric system, respectively. Furthermore, the docking outcomes of ligands to monomeric and dimeric shapes of SARS-CoV-2 Mpro well correlate each other with *R*^Monomer–Dimer^_Dock_ = 0.83 ± 0.08 and *R*^Monomer–Dimer^_Dock_ = 0.85 ± 0.09 corresponding to *medium* and *long* options (Fig. 4), respectively. Consequently, the RMSE between docking results unchange with values of RMSE^Monomer–Dimer^_Dock_ = 0.67 ± 0.10 and RMSE^Monomer–Dimer^_Dock_ = 0.67 ± 0.13 kcal mol^−1^ respecting to the docking option *medium* and *long* (Fig. 4), respectively. Overall, the docking simulations provide similar results when ligands docked to monomeric and dimeric systems and the default option of Autodock Vina is appropriate for performing docking simulations.

### MD-refined investigations

As mentioned above, the binding affinity of 24 available inhibitors to the monomeric and dimeric SARS-CoV-2 Mpro was appropriately probed using molecular docking calculations. However, it should be noted that the dynamics of receptors were not considered in docking simulations, and the number of trial docking poses was restricted. To overcome this limitation we have operated the atomistic simulations which serve as a validation for the docking results.^47–49^ Moreover, FPL is an efficient computational approach to assess ligand-binding affinity with a suitable time-consuming calculation.^50,51^ Furthermore, the scheme was successfully applied to the monomeric SARS-CoV-2 Mpro system recently.^36,37^ The FPL approach is thus used to probe the binding affinity of 24 available inhibitors to the monomeric and dimeric SARS-CoV-2 Mpro. In the simulations, the ligand binding pose was optimized over short canonical and isothermal–isobaric simulations. The equilibrated ligand was then pulled to translocate from *bound* to *unbound* states. The maximum of pulling force, called rupture force, and pulling work are typically assumed to correlate with ligand-binding affinity. It should be noted that the rupture force corresponds to the point that the non-covalent bond contact between a ligand and a receptor was terminated.

The computed values of the rupture force and pulling work were shown in Table 2. The denoted pulling force and work profiles were described in Tables S2 and S3 of the ESI file.† The shape of both pulling force and work appear reliable when compared to the previous exertion.^50,51^ In particular, starting at zero, the pulling force quickly increases to the maximum value, then suddenly drops to zero due to the loss the non-covalent bond contact to the receptor. During this process, recorded-pulling work speedily rises from zero value to a stable value, corresponding to the distance at which the contact between protein and inhibitor is vanished. Moreover, the rupture force *F*^Monomer^_Max_ of monomeric Mpros diffuses in the range from 295.0 to 977.6 pN corresponding with the spreading of pulling work *W*^Monomer^ from 13.7 to 106.1 kcal mol^−1^. Besides that, the matching metrics of dimeric Mpros forms in the range from 307.7 to 860.2 pN and 22.4 to 85.7 kcal mol^−1^, correspondingly. It should be noted that the computed works are significantly larger than the magnitude of experimental binding affinity, which diffuses in the range from 6.39 to 10.13 kcal mol^−1^, since applied large cantilever and high pulling velocity.^50^ Although the discrepancy can be reduced to zero by using a small cantilever and an extremely low pulling velocity, it is not appropriate since it requires to perform several trajectories with hundred nanoseconds each.^52^ Furthermore, previous investigations revealed that although reducing the magnitude of cantilever spring constant and pulling velocity was able to enlarge the accuracy of the estimations, the observed results are approximately the equivalent as those at high pulling velocity.^50^

**Table 2:** Computed values of rupture force and pulling work in comparison with experimental data of ligands to SARS-CoV-2 Mpro

No.	Name	*F* ^Monomer^_Max_	*W* ^Monomer^	*F* ^Dimer^_Max_	*W* ^Dimer^	Δ*G*_EXP_^a^
1	7j	575.3 ± 32.3	57.9 ± 3.9	583.6 ± 33.8	60.5 ± 4.2	−8.69
2	11a	761.0 ± 27.0	73.8 ± 3.3	860.2 ± 31.3	95.7 ± 3.9	−9.96
3	11b	735.4 ± 40.5	74.2 ± 3.9	814.0 ± 52.5	80.7 ± 4.9	−10.13
4	11r	724.8 ± 57.7	77.6 ± 7.1	636.6 ± 28.2	71.5 ± 2.9	−9.23
5	13a	526.9 ± 56.4	54.4 ± 7.3	769.6 ± 16.3	84.7 ± 3.2	−7.70
6	13b	977.6 ± 33.7	106.1 ± 4.6	739.1 ± 28.4	81.6 ± 3.0	−8.45
7	Calpain inhibitor I	625.0 ± 26.3	57.9 ± 2.7	683.2 ± 34.1	63.4 ± 2.5	−6.94
8	Calpain inhibitor II	592.5 ± 31.5	54.4 ± 3.5	497.4 ± 29.1	44.6 ± 4.3	−8.23
9	Calpain inhibitor XII	491.6 ± 20.5	46.0 ± 2.3	693.6 ± 50.7	63.5 ± 4.8	−8.69
10	Calpeptin	446.8 ± 16.9	33.4 ± 2.2	662.7 ± 32.5	62.5 ± 3.6	−6.81
11	Candesartan cilexetil	547.2 ± 38.0	51.4 ± 5.3	510.7 ± 39.3	49.7 ± 3.4	−7.60
12	Carmofur	485.5 ± 34.2	36.2 ± 2.7	436.9 ± 16.3	33.6 ± 1.8	−7.86
13	Chloroquine	363.4 ± 32.1	28.5 ± 2.8	410.9 ± 12.5	36.0 ± 1.6	−7.41
14	Dipyridamole	547.2 ± 38.0	51.4 ± 5.3	507.5 ± 18.7	51.0 ± 2.4	−8.52
15	Disulfiram	364.7 ± 24.7	22.7 ± 1.9	526.2 ± 30.3	40.1 ± 1.9	−6.89
16	GC-373	616.9 ± 34.0	58.2 ± 4.4	557.3 ± 39.9	52.0 ± 5.2	−8.76
17	Hydroxychloroquine	392.0 ± 27.2	30.2 ± 3.1	307.7 ± 24.9	22.4 ± 2.9	−7.58
18	MG-115	564.8 ± 26.4	56.6 ± 2.5	708.8 ± 31.1	70.6 ± 3.5	−7.53
19	MG-132	543.2 ± 22.2	49.8 ± 2.1	505.7 ± 41.1	47.5 ± 6.0	−7.41
20	Narlaprevir	601.8 ± 31.9	64.8 ± 2.8	522.0 ± 38.3	54.7 ± 4.3	−7.18
21	Omeprazole	478.6 ± 24.0	38.1 ± 2.2	413.3 ± 33.1	31.7 ± 3.2	−6.40
22	Oxytetracycline	447.2 ± 21.6	37.0 ± 2.9	432.4 ± 49.6	37.7 ± 4.8	−6.60
23	PX-12	295.0 ± 17.4	13.7 ± 1.2	382.0 ± 25.5	27.2 ± 2.0	−6.39
24	Shikonin	321.8 ± 29.7	19.7 ± 3.0	504.5 ± 22.8	39.1 ± 1.2	−6.58

a The experimental binding free energies were gained based on IC_50_ value,^12–18^ approximating that the one equals to the inhibition constant *k*_i_. The unit of force and energy/work are in pN and kcal mol^−1^, respectively.

**Fig. 5:**
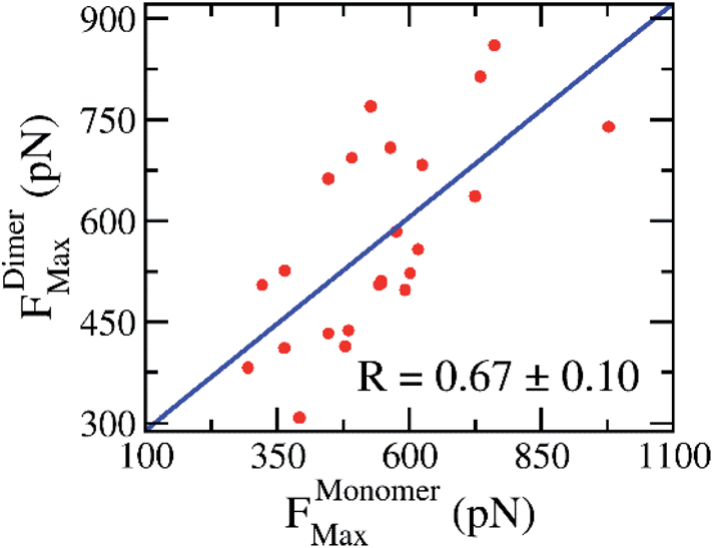
Relationship between rupture forces of the SARS-CoV-2 Mpro monomer and dimer. Rupture forces were obtained *via* FPL calculations. The computed error was attained *via* 1000 rounds of the bootstrapping method.^44^

In practice, the rupture force has been used as a predictor of ligand-binding affinity based on the assumption that a ligand binds with a higher affinity requires a stronger pulling force to dissociate it from binding cleft.^53^ Using the rupture force as a proxy to ligand-binding affinity, numerous investigations were successful in predicting the ligand-binding affinity to various targets.^53,54^ Here, the average of rupture forces were estimated over 8 independent FPL trajectories (*cf.*Table 2). The correlation coefficient to experiments, obtained results of monomeric systems, is *R*^Monomer^_Force_ = −0.69 ± 0.10; while the analogous value of dimeric forms is *R*^Dimer^_Force_ = −0.60 ± 0.15. Because the correlation coefficients appear to be the same within the error range, we may conclude that there is no difference when using monomer or dimer as a CADD target. Moreover, the correlation between rupture forces obtained over SARS-CoV-2 Mpro monomer and dimer is appropriate with a value of *R*^Monomer–Dimer^_Force_ = 0.67 ± 0.10 (*cf.*Fig. 5). It may be argued that the recorded rupture forces of the SARS-CoV-2 Mpro monomer is quite similar to the dimeric one, although the correlation is smaller than obtained values *via* docking approach.

The work of pulling force was assessed *via* formula 
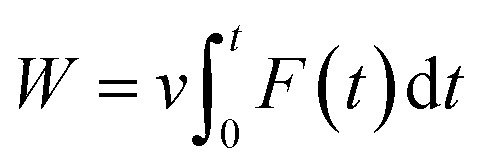
, where *v* is pulling velocity and *F*(*t*) is pulling force. In isothermal–isobaric simulations, *W* is related to the experimental binding affinity *via* Jarzynski equality.^55^ Therefore, utilizing W to estimate the ligand-binding affinity commonly acquires a better accurate result in comparison to rupture force.^47,50,54^ The obtained results reaffirmed this statement. The correlation coefficients of the monomeric and dimeric SARS-CoV-2 Mpro are *R*^Monomer^_Work_ = −0.69 ± 0.09 and *R*^Dimer^_Work_ = −0.65 ± 0.13, respectively. Although, the computational accuracy targeting the SARS-CoV-2 Mpro monomer is slightly larger than that of the dimeric system, the difference in correlation coefficients is insignificant implying that the monomeric form of SARS-CoV-2 Mpro can be used as CADD target instead of the dimeric one. Moreover, it should be noted that FPL outcomes based on the short NPT simulation of only 2.0 ns. The results possibly limited since the complex may not gain the equilibrium states as reported in the recent work.^45^

**Fig. 6:**
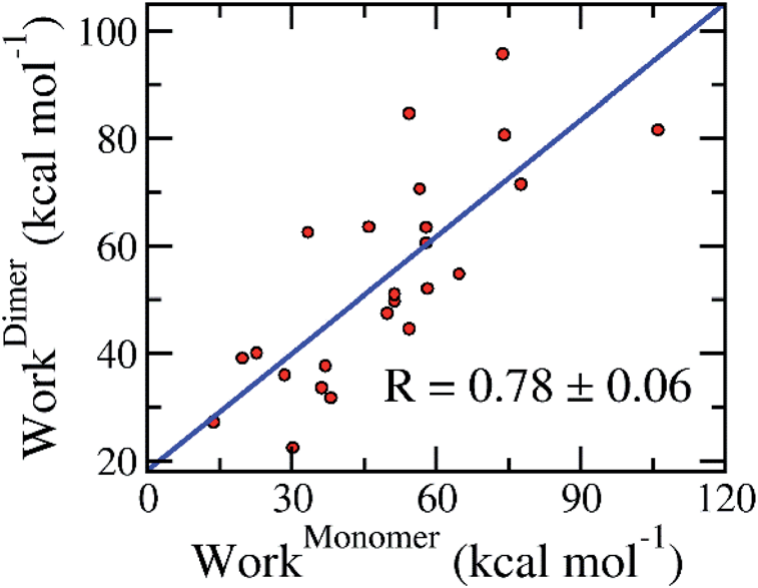
Association between calculated pulling work of the monomeric and dimeric SARS-CoV-2 Mpro. The computed error was attained *via* 1000 rounds of the bootstrapping method.^44^

The association of computed pulling works of the monomeric and dimeric SARS-CoV-2 Mpro was probed and shown in Fig. 6. Over the bootstrapping examination, the correlation coefficient is *R*^Monomer–Dimer^_Work_ = 0.78 ± 0.06 confirming the observation above. The non-enhancement of FPL outcomes compared with docking results possibly occurs due to the increase of the different binding poses to various targets, which was described below. Overall, it may be concluded that we can perform the inhibitor screening for SARS-CoV-2 Mpro with smaller computing resources since targeting the monomeric form.

In addition, the MD-refined ligand-binding affinity results are confirmed since the RMSD between ligand-binding poses to the monomeric and dimeric forms is of 0.23 ± 0.02 nm only. It should be noted that the RMSD metrics were calculated based on the last snapshot of NPT simulations, which were utilized for the binding free energy prediction *via* the FPL scheme. Moreover, the RMSD of MD-refined structure was slightly larger than docking results due to the effects of the conformational entropy. Moreover, the intermolecular hydrogen bond analyses suggests that three residues including Thr25, Asn142, Gly143, and Glu166 are critical residues controlling the binding mechanism of the inhibitors to both monomeric and dimeric SARS-CoV-2 Mpro (*cf.* Table S4 of the ESI†).

### Evaluation of some inhibitors of SARS-CoV-2

The binding affinity of 19 available SARS-CoV-2 inhibitors to the protease was also computed. The obtained results were reported in Tables 3 and S5–S7 of the ESI file.† Although IC_50_ of these inhibitors was taken from cell-culture experiments^21^ implying that drug targets possibly differ from the protease as well as RNA polymerase, appropriate correlations between computed outcomes and respective experiments were obtained. Therefore, it may be concluded that many compounds inhibit SARS-CoV-2 Mpro. In particular, Autodock Vina adopts a larger correlation coefficient on the monomer, *R*^Monomer^_Vina_ ≈ 0.84, than dimer, *R*^Dimer^_Vina_ ≈ 0.52, whereas the *R*^Monomer–Dimer^_Dock_ ≈ 0.70 implies the consistency of our observation. The SARS-CoV-2 Mpro + inhibitor structure provided by the docking approach was then used as the starting shape for FPL calculation. The association between computed values of the monomeric and dimeric Mpro were obtained. The disparity attained due to some compounds possibly aiming on the polymerase rather than the protease.^19^

**Table 3:** Computed values of docking, rupture force and pulling work in comparison with experimental data of ligands to SARS-CoV-2

No.	Name	Δ*G*^Monomer^_Dock_			Δ*G*^Dimer^_Dock_			*F* ^Monomer^_Max_	*W* ^Monomer^	*F* ^Dimer^_Max_	*W* ^Dimer^	Δ*G*_EXP_^a^
		Short	Medium	Long	Short	Medium	Long					
1	Bazedoxifene	−7.4	−7.5	−7.4	−7.4	−7.4	−7.5	460.3 ± 26.0	41.2 ± 3.1	471.1 ± 20.0	47.5 ± 3.6	−7.48
2	Cyclosporine	−5.8	−5.7	−5.7	−5.4	−5.4	−5.4	638.8 ± 33.4	67.7 ± 5.4	426.5 ± 41.6	44.1 ± 4.7	−7.17
3	Digitoxin	−8.1	−8.1	−8.2	−7.0	−7.0	−7.2	667.4 ± 17.7	70.9 ± 2.1	502.6 ± 65	55.3 ± 8.3	−9.09
4	Digoxin	−8.1	−8.1	−8.1	−7.1	−7.2	−7.2	637.0 ± 30.3	75.0 ± 2.5	573.1 ± 42.3	59.4 ± 4.9	−9.20
5	Dihydrogambogic acid	−7.0	−7.0	−7.0	−7.2	−7.2	−7.2	542.8 ± 37.7	59.6 ± 3.2	487.5 ± 29.9	44.0 ± 3.3	−6.67
6	Ebastine	−5.7	−6.5	−6.1	−6.5	−6.3	−6.4	447.5 ± 40.1	40.2 ± 3.5	389.8 ± 25.0	32.8 ± 2.8	−7.06
7	Favipiravir	−4.5	−4.8	−4.8	−5.0	−5.0	−5.0	364.9 ± 26.2	21.3 ± 2.9	336.1 ± 19.1	20.5 ± 2.5	−4.52
8	Fluspirilene	−6.9	−7.2	−7.3	−8.0	−7.7	−7.6	490.1 ± 23.6	43.8 ± 2.0	544.6 ± 36.3	58.0 ± 3.2	−7.53
9	Isoosajin	−7.7	−7.7	−7.7	−8.0	−8.0	−8.0	393.1 ± 32.8	28.9 ± 3.2	454.4 ± 19.7	40.4 ± 2.5	−7.52
10	Ivacaftor	−6.7	−6.7	−6.7	−7.2	−7.6	−7.5	347.9 ± 34.8	22.3 ± 4.4	477.5 ± 22.1	41.0 ± 2.1	−7.10
11	Lusutrombopag	−6.2	−6.1	−6.8	−6.4	−6.5	−6.3	540.6 ± 37.5	59.1 ± 3.7	396.8 ± 24.3	41.8 ± 2.2	−7.42
12	Mefloquine	−6.5	−6.5	−6.5	−7.6	−7.7	−7.6	523.7 ± 23.5	41.5 ± 2.3	509.6 ± 43.3	46.3 ± 3.3	−7.34
13	Mequitazine	−6.6	−6.6	−6.6	−6.3	−6.3	−6.3	392.5 ± 51.3	29.5 ± 4.0	384.9 ± 24.4	29.0 ± 2.2	−7.03
14	Osajin	−6.8	−6.9	−6.8	−7.6	−8.0	−8.0	367.9 ± 20.4	30.8 ± 2.9	471.4 ± 23.9	39.8 ± 1.8	−7.41
15	Oxyclozanide	−6.4	−6.4	−6.4	−6.7	−6.7	−6.7	463.7 ± 31.7	33.6 ± 3.2	468.1 ± 13.3	39.2 ± 3.5	−7.44
16	Penfluridol	−7.0	−6.9	−6.9	−8.0	−8.2	−8.2	542.3 ± 33.1	53.3 ± 2.7	444.5 ± 25.0	48.0 ± 3.9	−7.26
17	Phenazopyridine	−6.0	−6.0	−6.0	−6.0	−6.0	−6.0	391.7 ± 36.2	25.6 ± 2.8	384.8 ± 22.7	32.4 ± 1.4	−6.23
18	Proscillaridin	−7.7	−7.7	−7.7	−6.8	−7.3	−7.3	485.6 ± 37.2	45.8 ± 3.3	512.8 ± 18.9	58.0 ± 1.6	−7.79
19	Tetrandrine	−6.6	−6.6	−6.6	−6.8	−6.8	−6.8	485.6 ± 37.2	45.8 ± 3.3	401.5 ± 18.5	31.6 ± 1.8	−7.56

a The experimental binding free energies were gained based on IC_50_ value,^20,21^ approximating that the one equals to the inhibition constant *k*_i_. The unit of force and energy/work are in pN and kcal mol^−1^, respectively.

## Conclusions

Both of Autodock Vina and FPL simulations were confirmed to be able to appropriately estimate the ligand-binding affinity of the SARS-CoV-2 Mpro in both monomeric and dimeric forms. The assessed results suggested that the monomeric form of SARS-CoV-2 Mpro can be used as a CADD target instead of the dimeric form. In particular, the high correlation coefficients between computational ligand-binding affinities of the monomeric and dimeric SARS-CoV-2 Mpro *via* docking and FPL simulations are obtained, which are *R*^Monomer–Dimer^_Dock_ = 0.85 ± 0.09 and *R*^Monomer–Dimer^_Work_ = 0.78 ± 0.06, respectively. Moreover, the correlation coefficient between the rupture forces of two targets is roughly appropriate with a value of *R*^Monomer–Dimer^_Force_ = 067 ± 0.10. It should be noted that the observation is in good agreement with structure analyses with the RMSD between ligand-binding pose to the monomeric and dimeric forms of 0.21 ± 0.02 and 0.23 ± 0.02 nm for docking and MD-refined structures, respectively. Furthermore, the appearance of different binding poses possibly results in the non-perfect correlation between the computational values of two targets.

The obtained computational values of the monomeric and dimeric SARS-CoV-2 Mpro correlate to experiments in similar amounts. The docking approach formed a Pearson correlation of *ca. R*_Dock_ = 0.50 to both targets. FPL approach enhanced the accuracy of the calculated ligand-binding affinity since a correlation is *ca. R*_Work_ ≈ −0.65 over both receptors. The accuracy of FPL simulations probably increases due to extending the NPT simulation time as reported in the recent work.^45^

In addition, in good agreement with the previous observation,^35^ the molecular docking by Vina package rapidly converged since the correlation coefficient between computed and experimental values did not change when the docking option was altered. The RMSE of docking results also unchanged upon these alterations. Finally, it may be concluded that for SARS-CoV-2 Mpro system the pulling work is better than rupture force in predicting the ligand-binding affinity. It is well compatible with earlier probe various protein–ligand complexes.^47,50,54^

## Conflicts of interest

There are no conflicts to declare.

## Supplementary Material

RA-011-D0RA09858B-s001
