# Supplementary material for: Binding of inhibitors to the monomeric and dimeric SARS-CoV-2 Mpro
Source: RSC Adv. 2021 Jan 13;11(5):2926–34. doi: 10.1039/d0ra09858b (PMC8694027; doi:10.1039/d0ra09858b)
Supplement: RA-011-D0RA09858B-s001 [file RA-011-D0RA09858B-s001.pdf]

Electronic Supplementary Material (ESI) for RSC Advances.
This journal is © The Royal Society of Chemistry 2021

Supporting information

Binding of Inhibitors to the Monomeric and Dimeric SARS-CoV-2 Mpro

Nguyen Minh Tam,[ab] Pham Cam Nam,[c] Duong Tuan Quang,[d] Nguyen Thanh Tung,[ef] and Van V. Vu,[g] and Son Tung Ngo[bh*]

[a]*Computational Chemistry Research Group, Ton Duc Thang University, Ho Chi Minh City, Vietnam*

[b]*Faculty of Applied Sciences, Ton Duc Thang University, Ho Chi Minh City, Vietnam*

[c]Department of Chemistry, The University of Danang, University of Science and Technology, Danang, Vietnam

[d]*University of Education, Hue University, Vietnam*

[e]Institute of Materials Science, Vietnam Academy of Science and Technology, Hanoi, Vietnam

[f]*Graduate University of Science and Technology, Vietnam Academy of Science and Technology, Hanoi, Vietnam*

[g]*NTT Hi-Tech Institute, Nguyen Tat Thanh University, Ho Chi Minh City, Vietnam*

[h]*Laboratory of Theoretical and Computational Biophysics, Ton Duc Thang University, Ho Chi Minh City, Vietnam*

Email: ngosontung@tdtu.edu.vn

Table S1. The binding pose of ligands to SARS-CoV-2 Mpro via PyMOL.

Name	Monomer	Dimer
7J	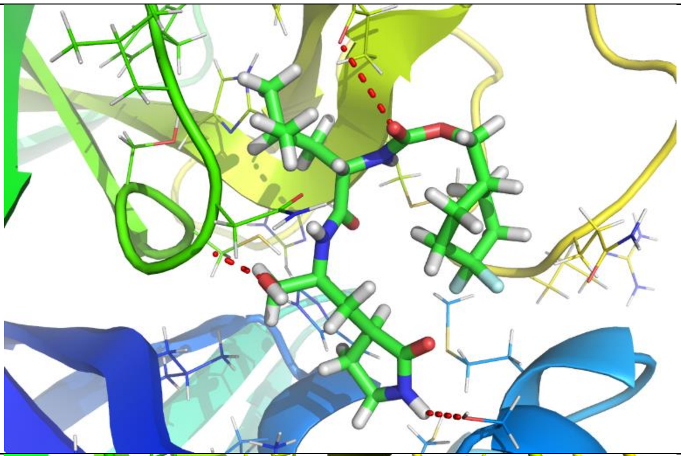	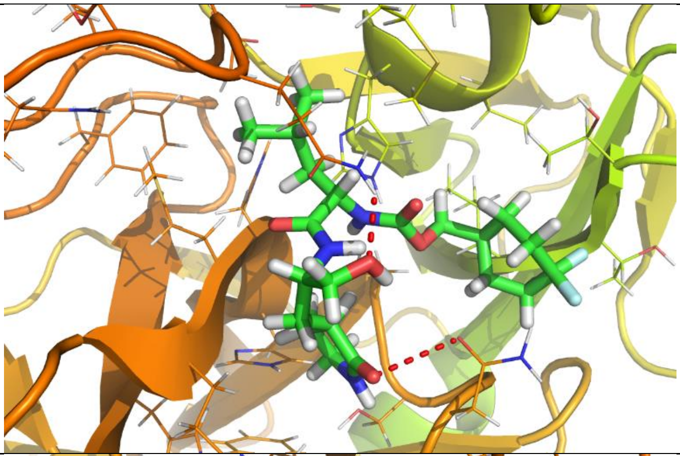
11a	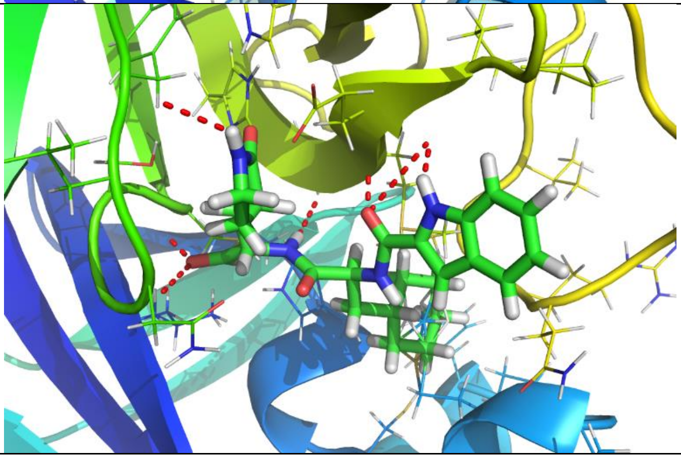	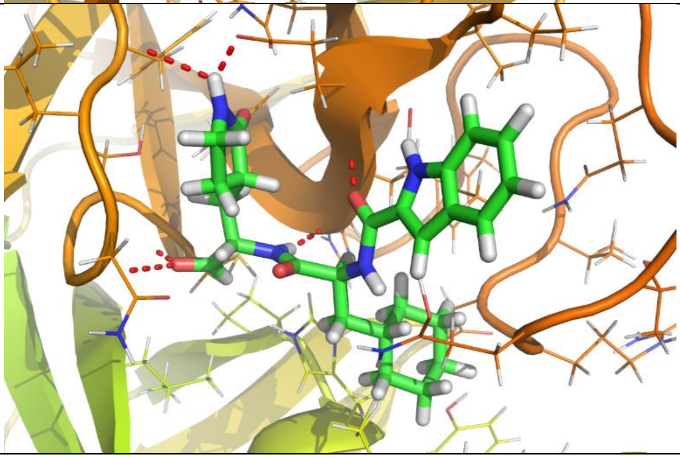

11b	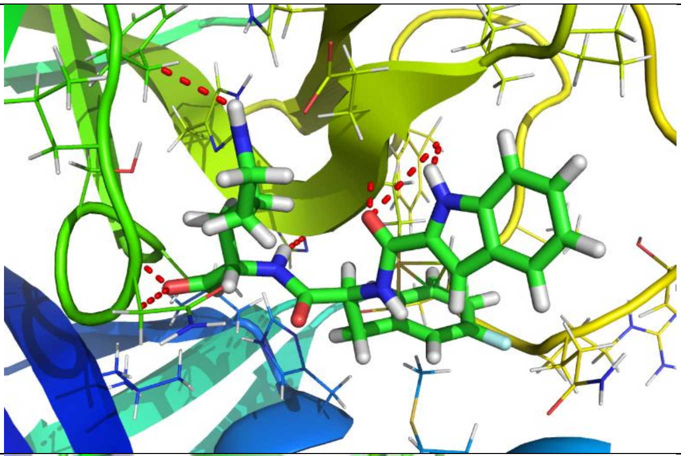	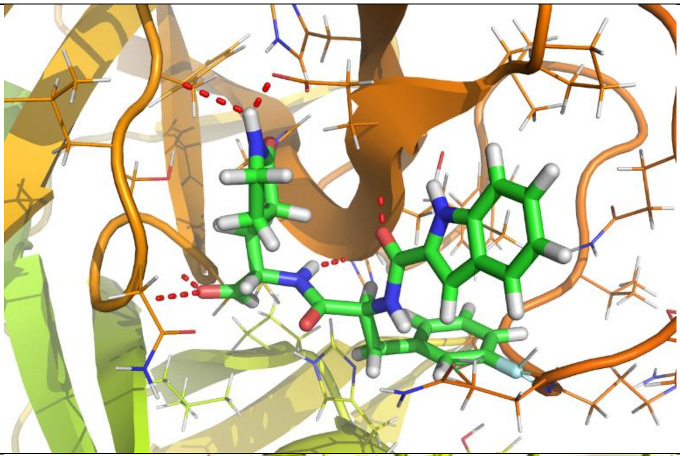
11r	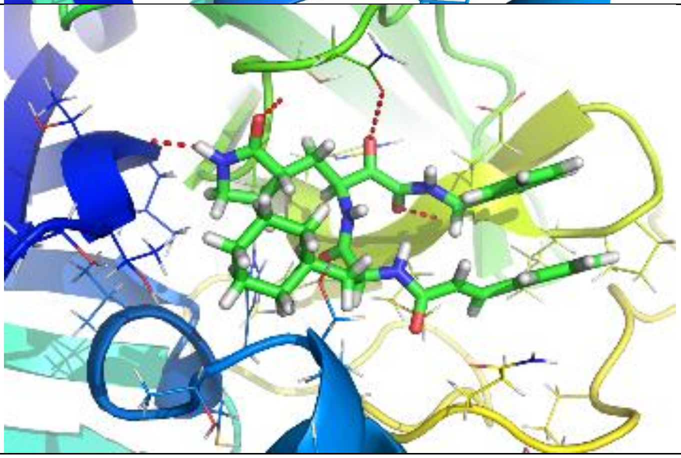	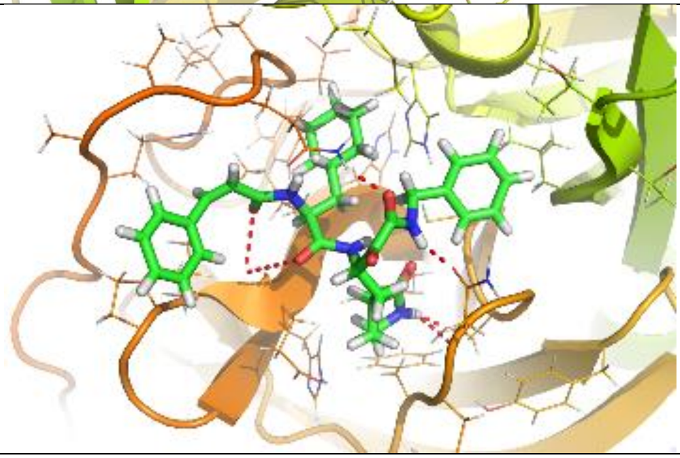

13a	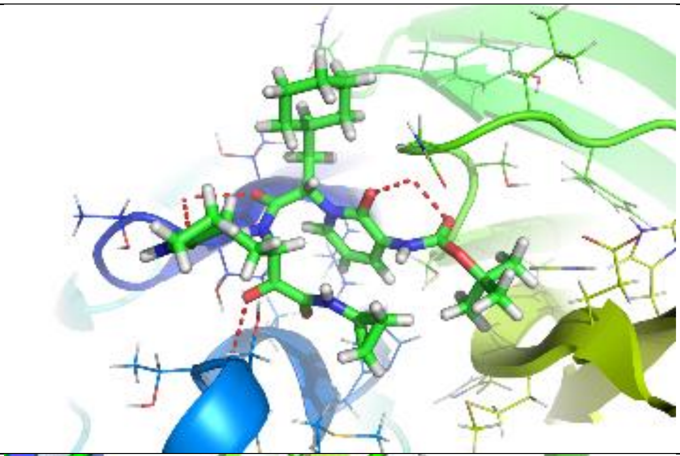	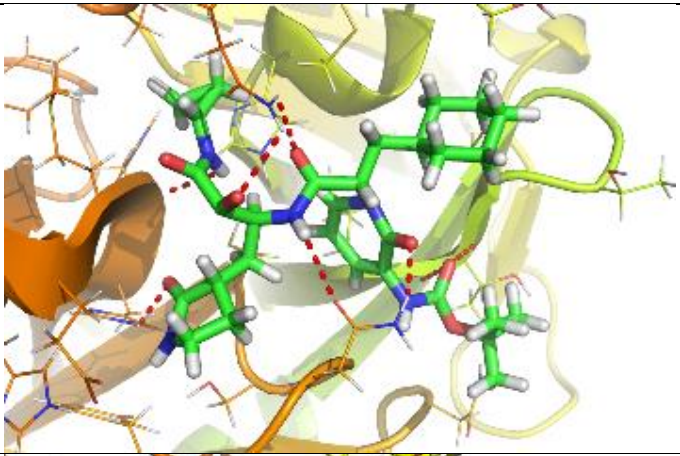
13b	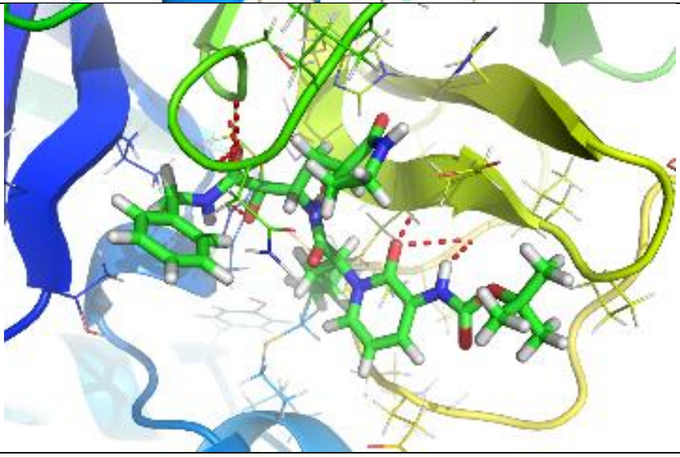	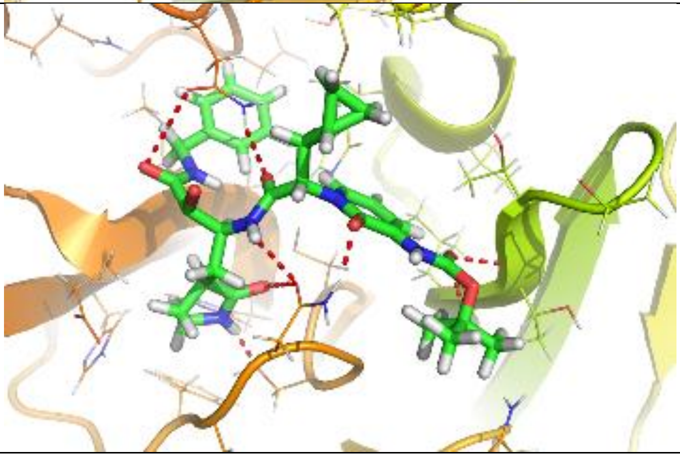

Calpain inhibitor I	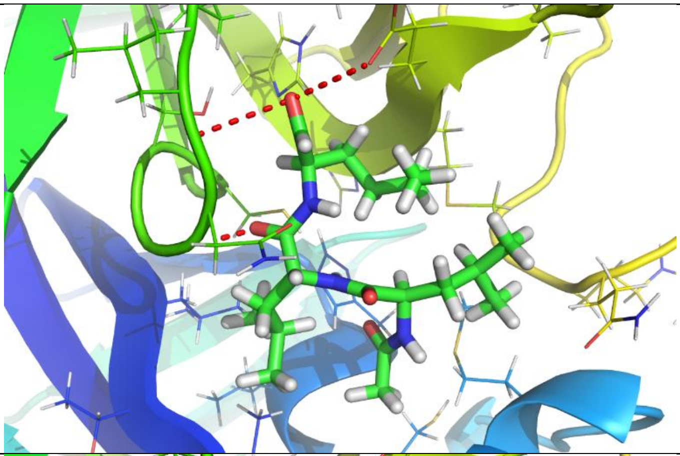	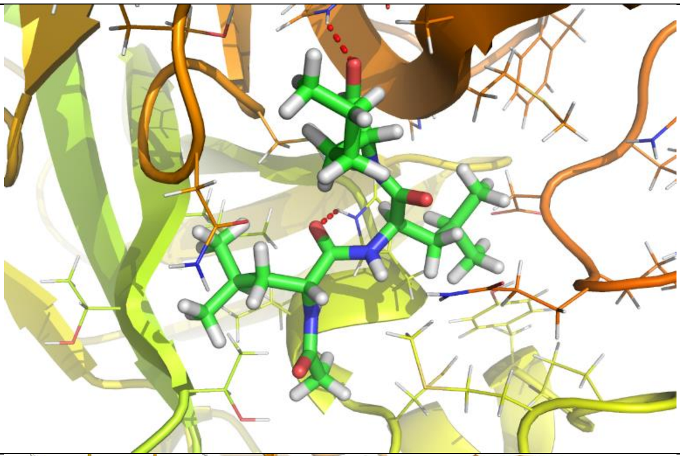
Calpain inhibitor II	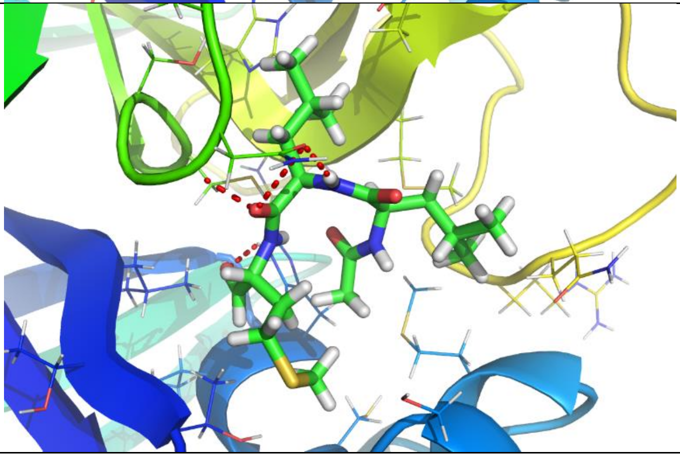	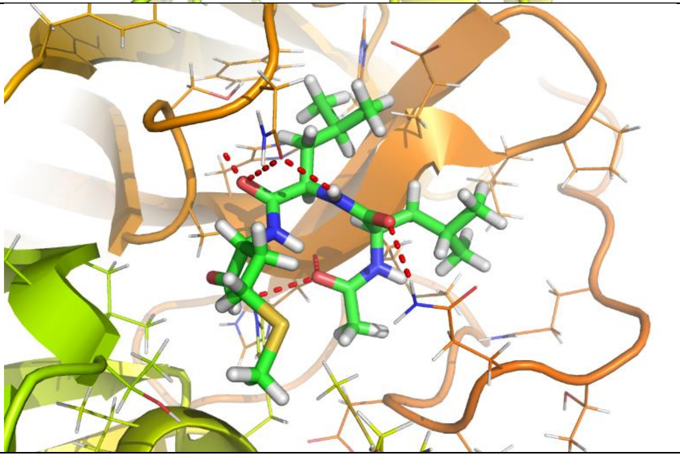

Calpain inhibitor XII	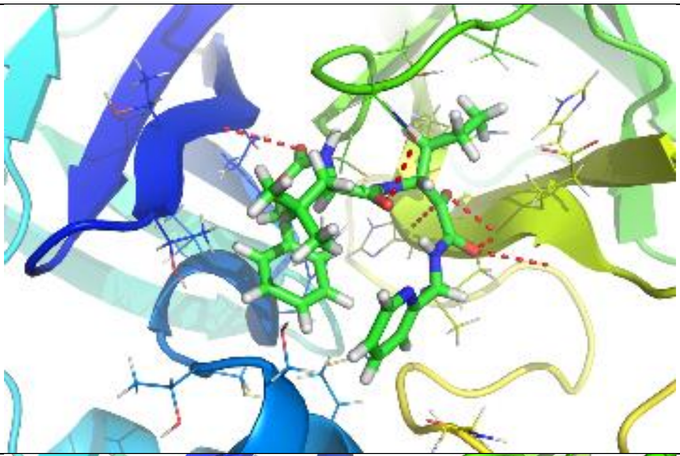	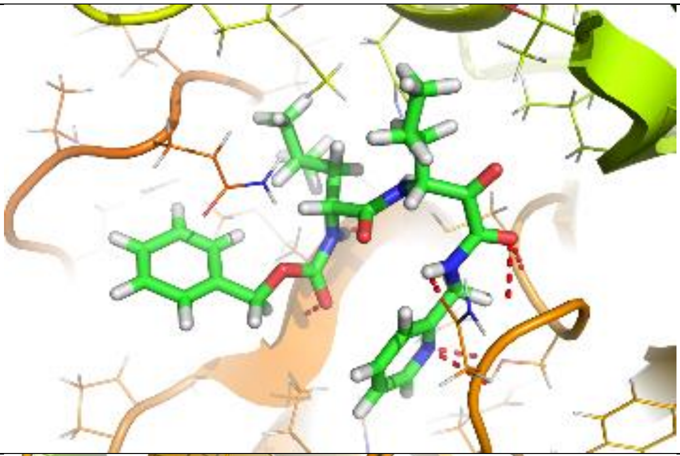
Calpeptin	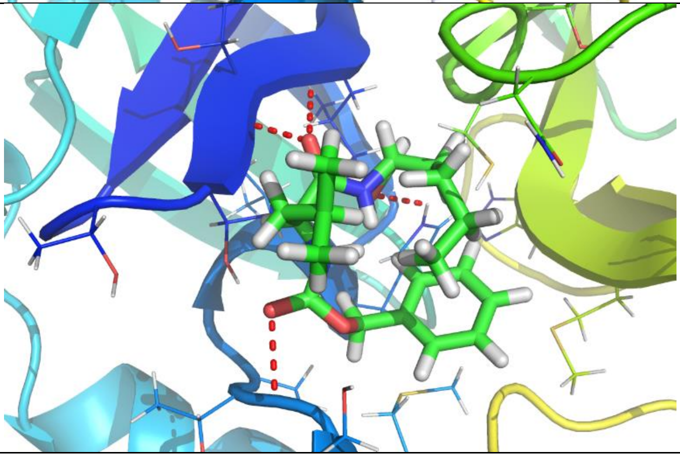	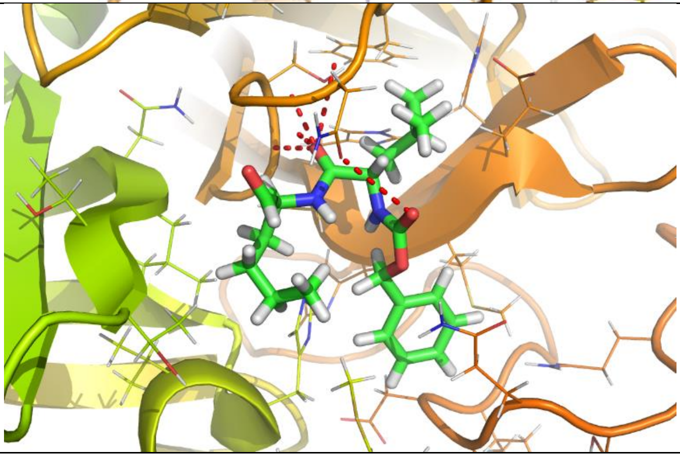

Candesartan cilexetil	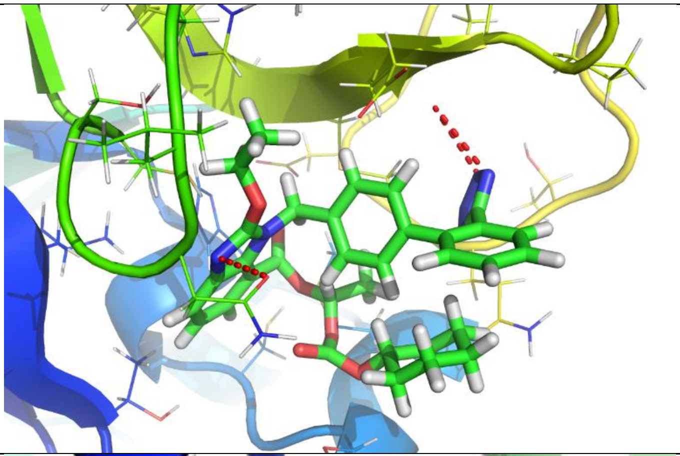	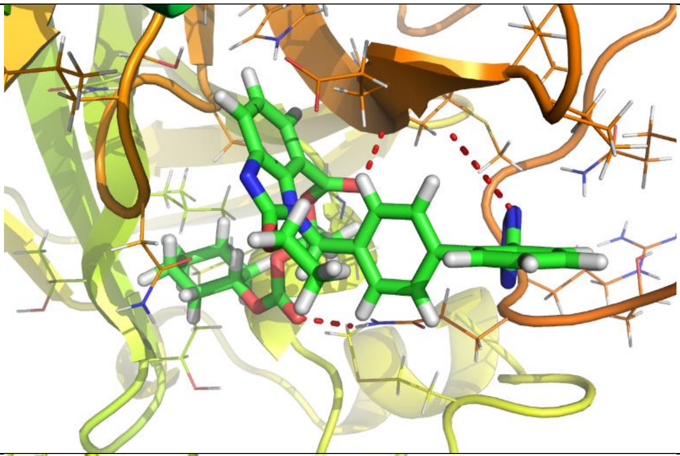
Carmofur	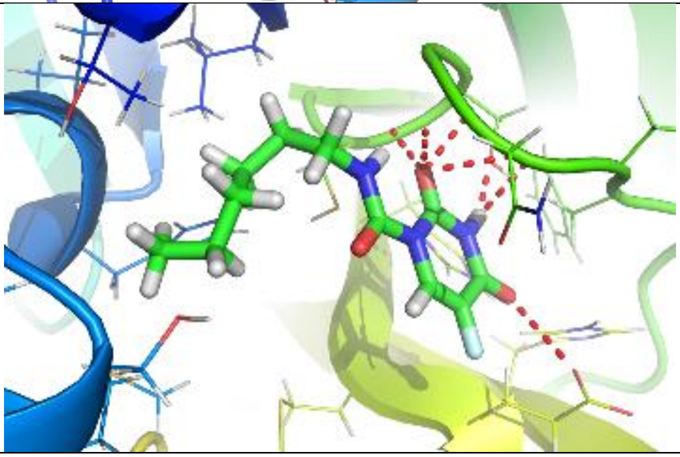	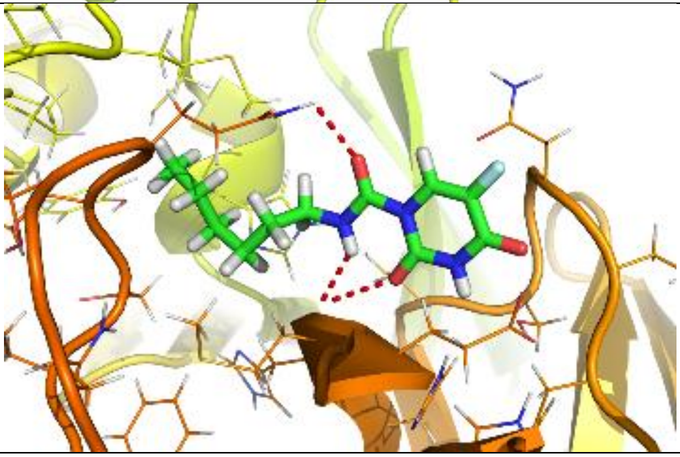

Chloroquine	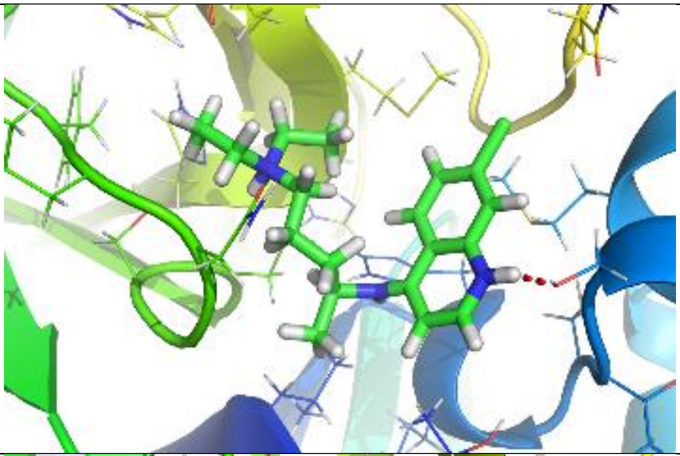	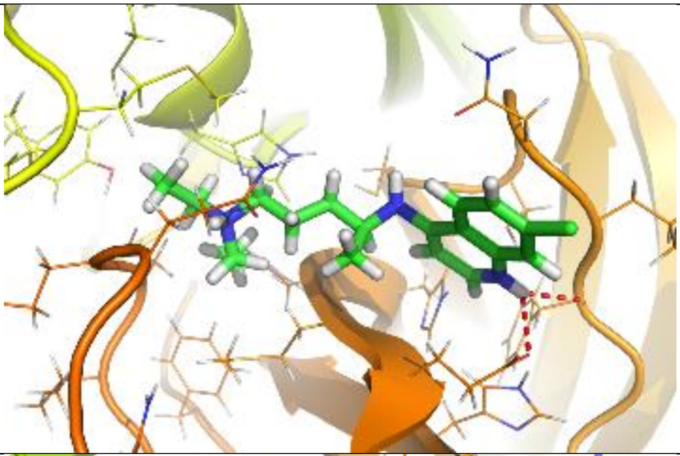
Dipyridamole	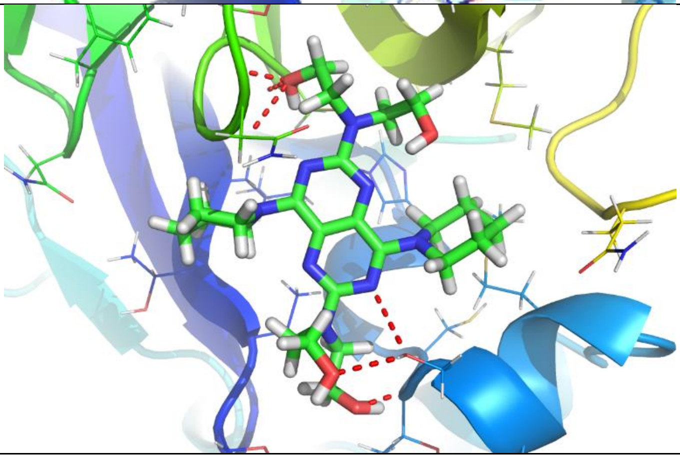	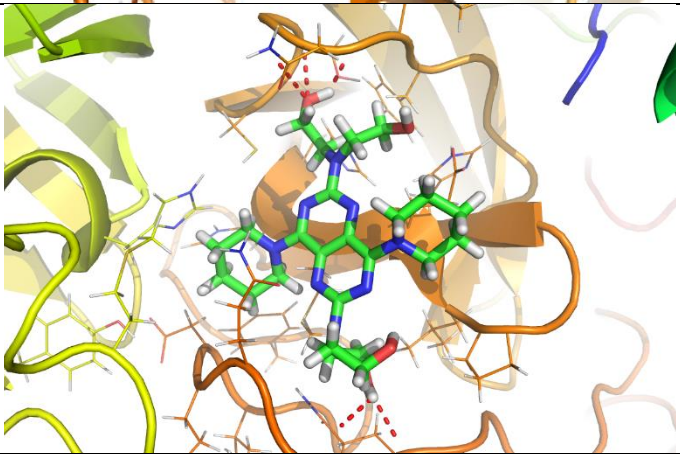

Disulfiram	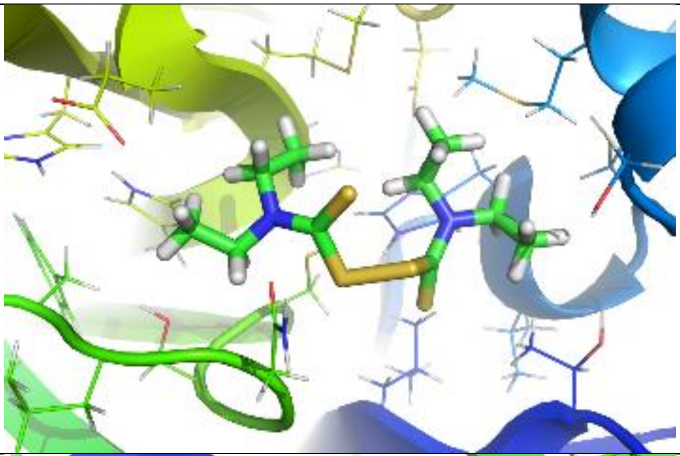	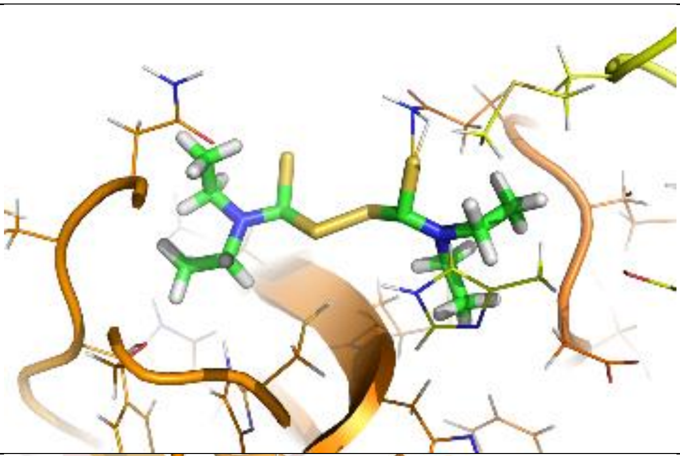
GC-373	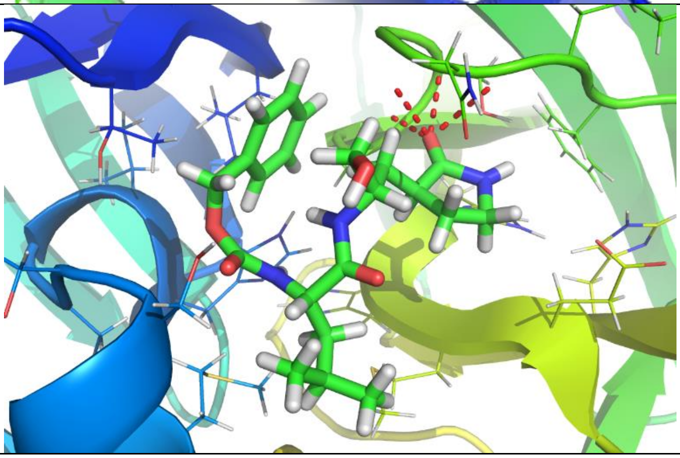	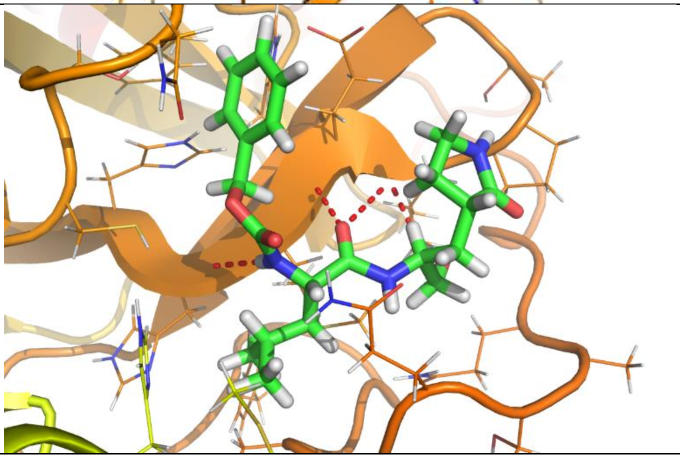

Hydroxychloroquine	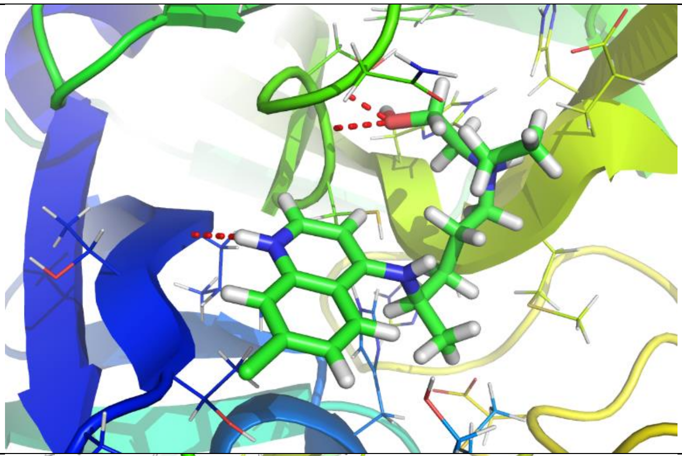	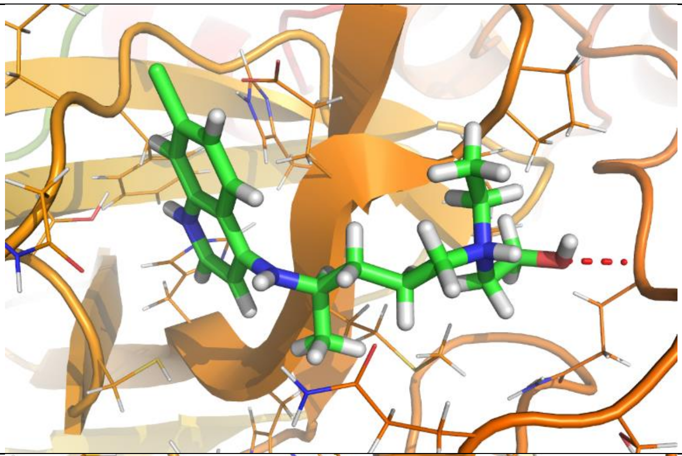
MG-115	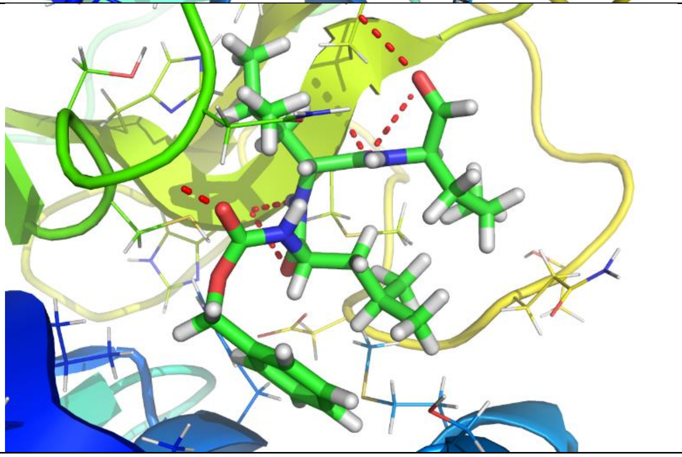	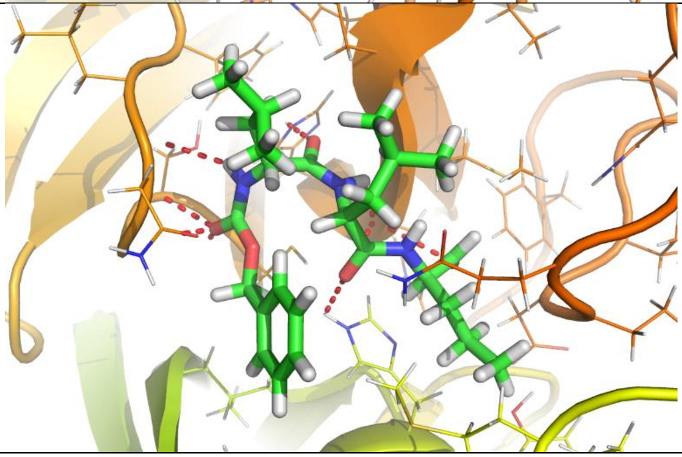

MG-132	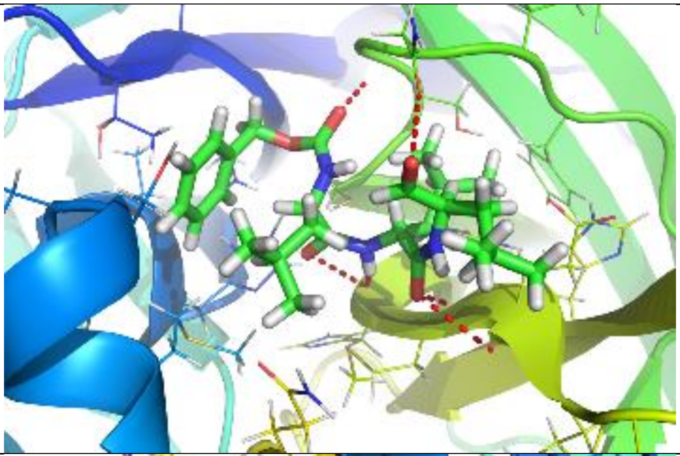	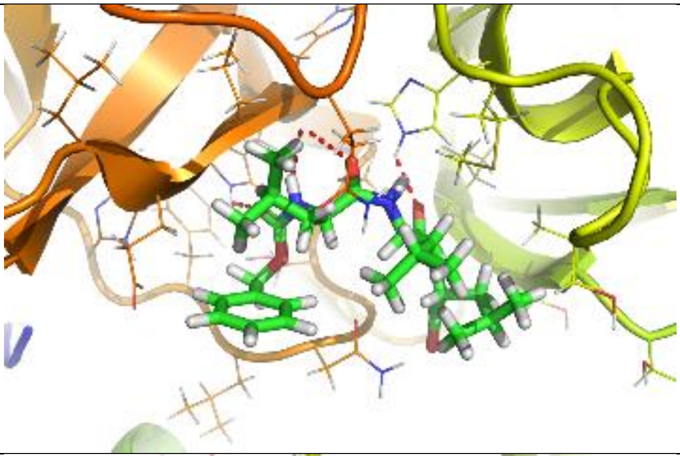
Narlaprevir	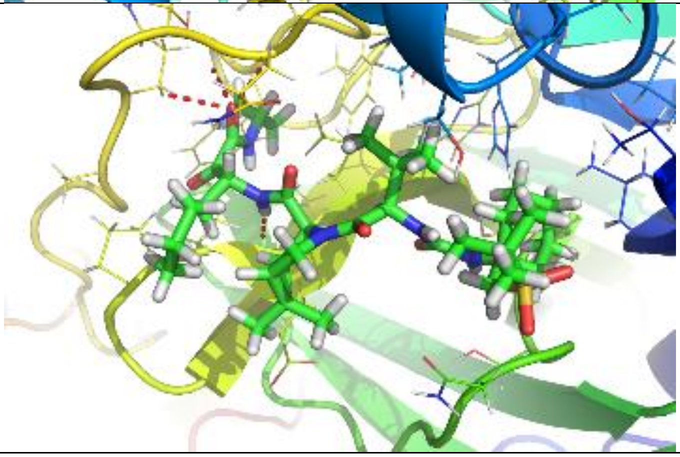	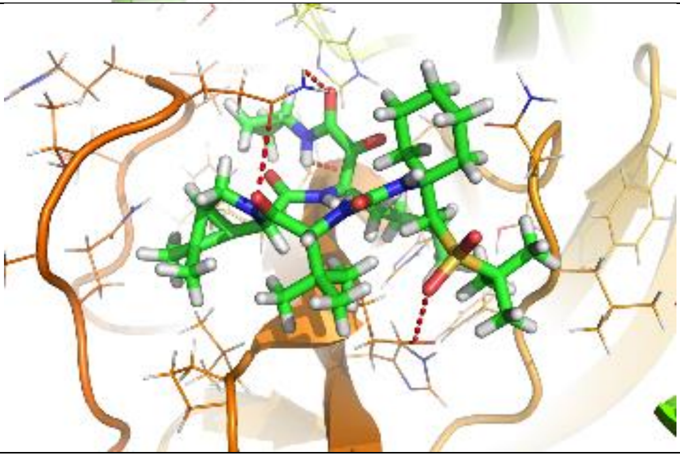

Omeprazole	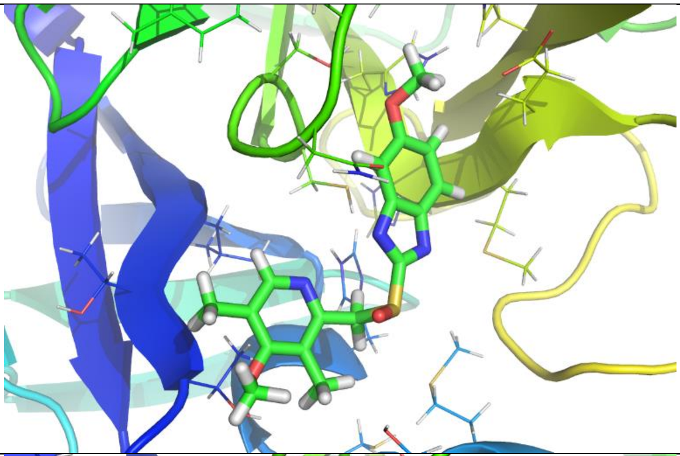	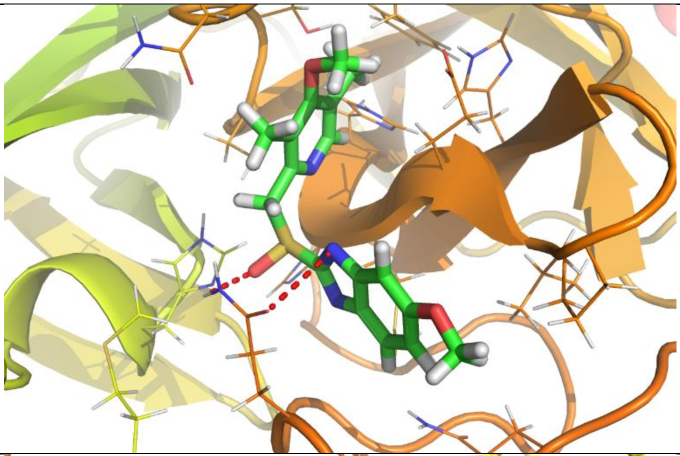
Oxytetracycline	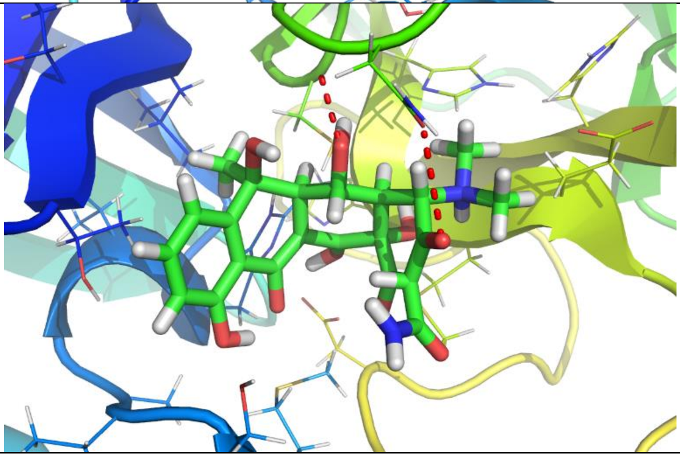	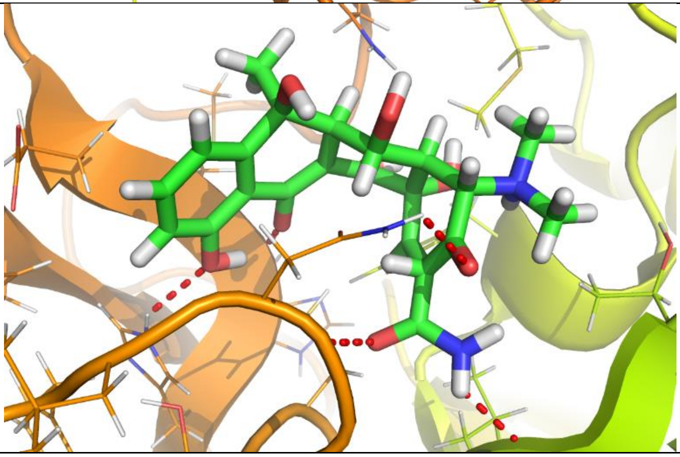

PX-12	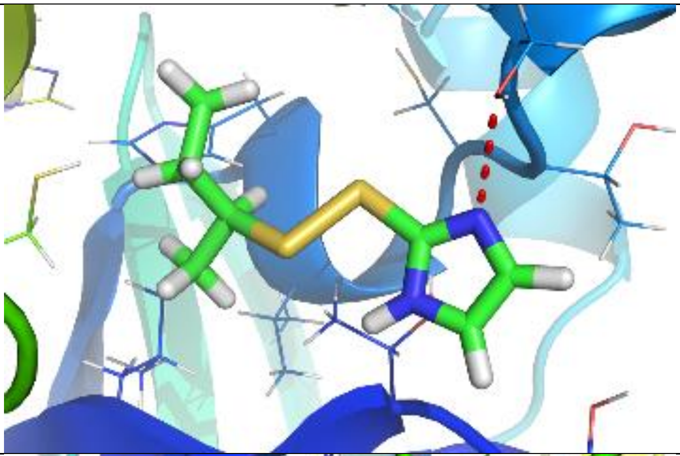	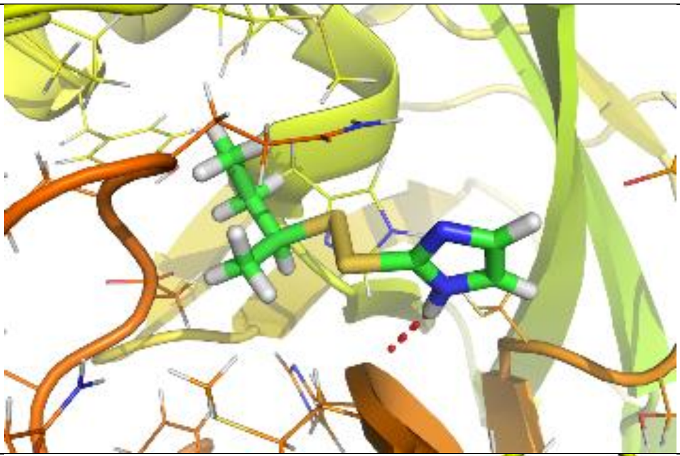
Shikonin	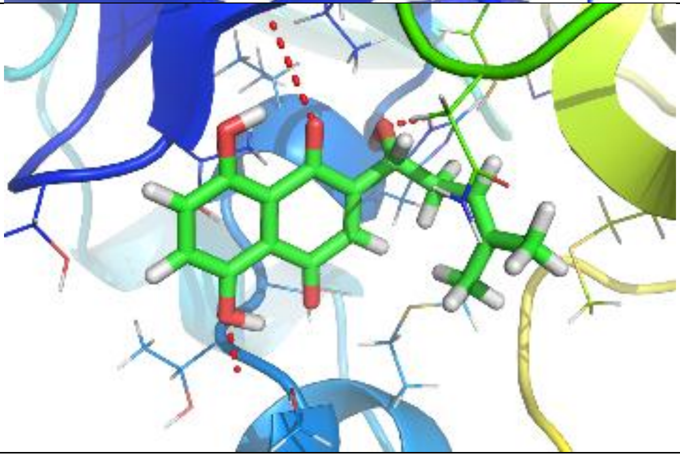	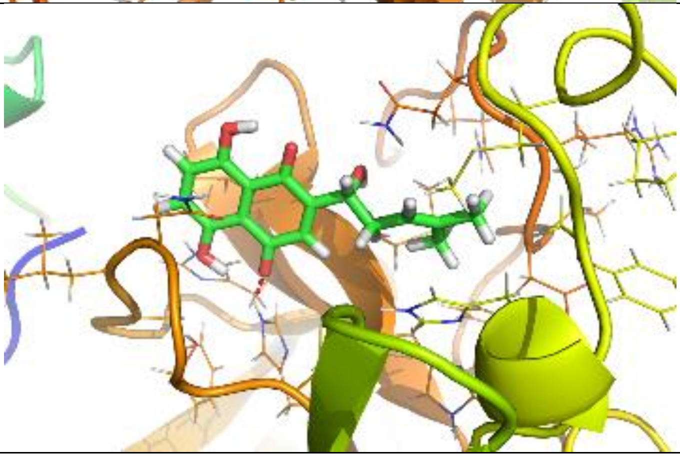

Table S2. The Pulling Force in Time Evolution.

Name	Monomer	Dimer
7J	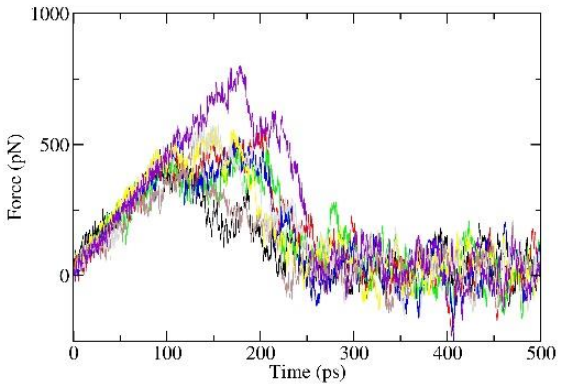	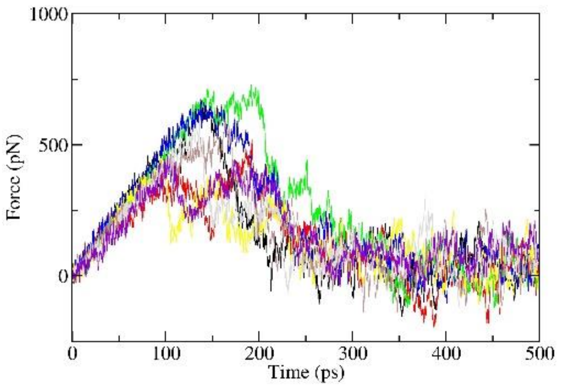
11a	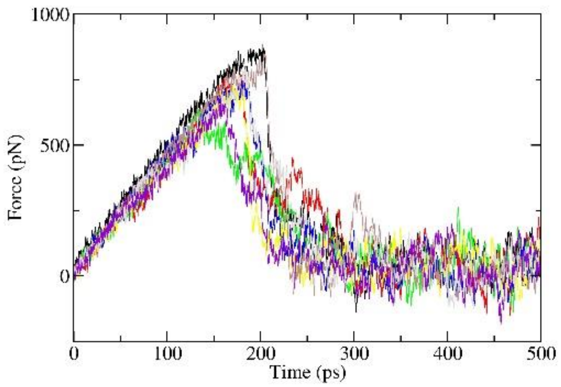	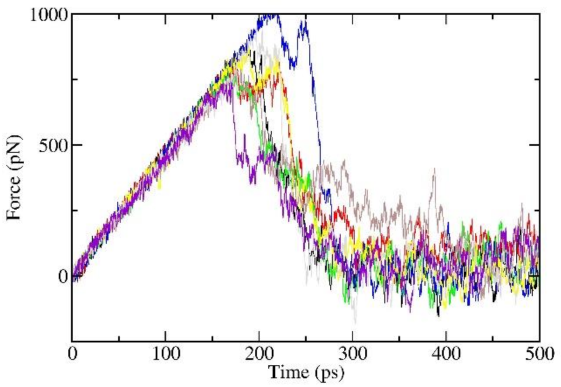

**11b**	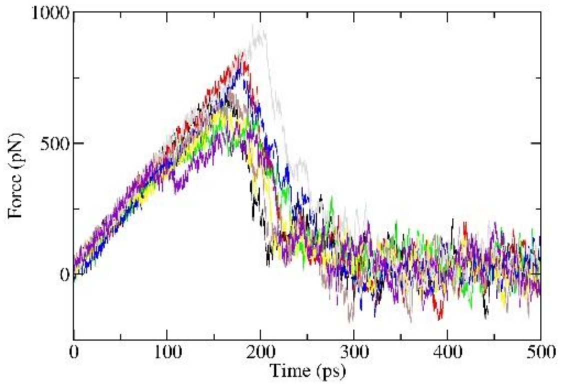Force (pN)<br>Time (ps)<br>1000 500 0<br>0 100 200 300 400 500	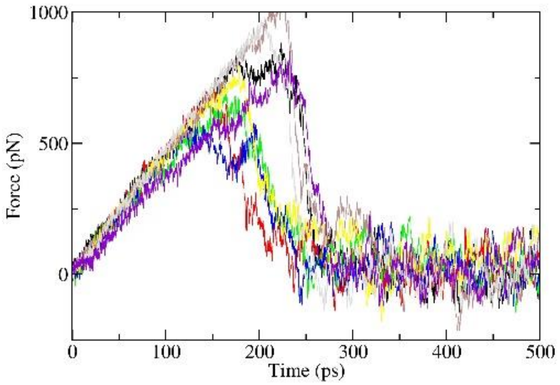Force (pN)<br>Time (ps)<br>1000 500 0<br>0 100 200 300 400 500
**11r**	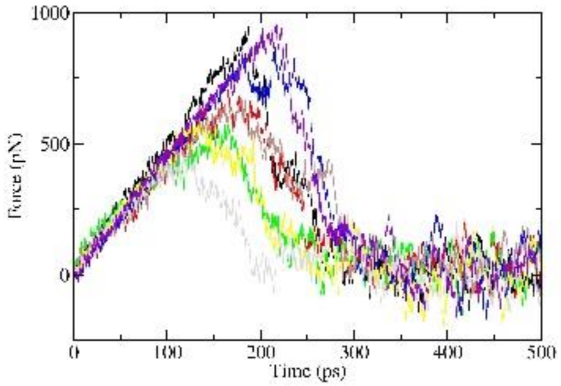Force (pN)<br>Time (ps)<br>1000 500 0<br>0 100 200 300 400 500	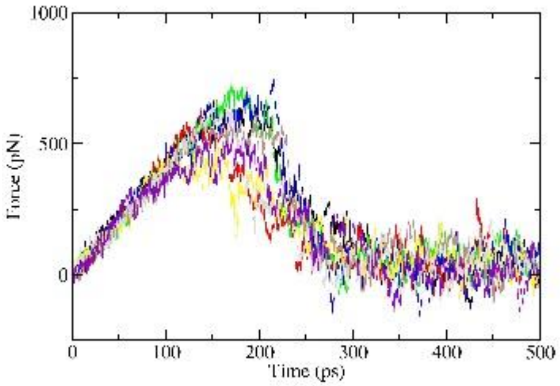Force (pN)<br>Time (ps)<br>1000 500 0<br>0 100 200 300 400 500

13a

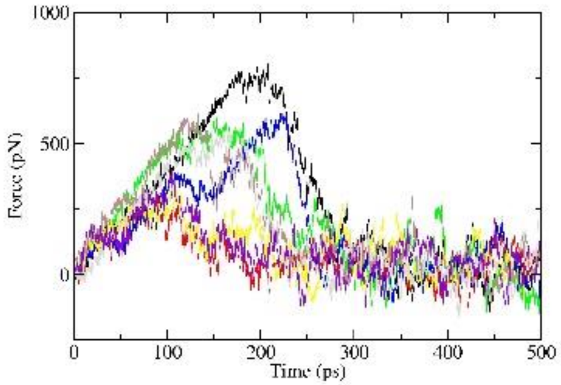
Force (pN)
Time (ps)
1000
500
0
0
100
200
300
400
500

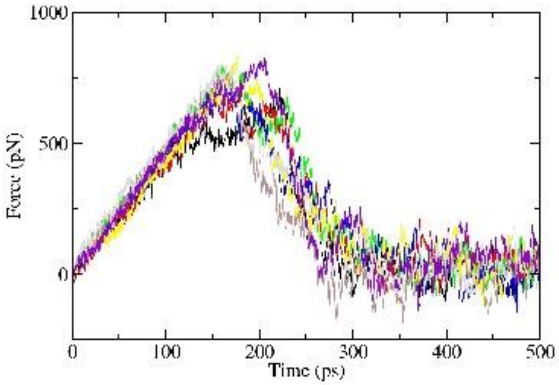
Force (pN)
Time (ps)
1000
500
0
0
100
200
300
400
500

13b

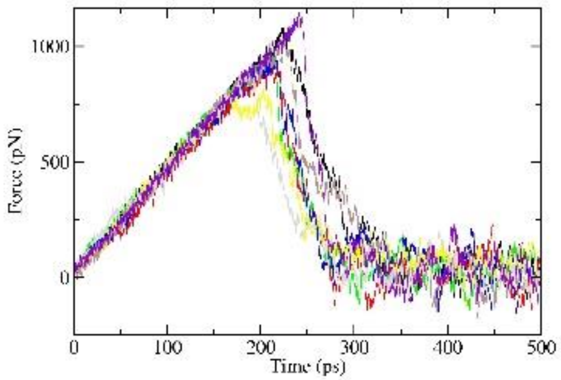
Force (pN)
Time (ps)
1000
500
0
0
100
200
300
400
500

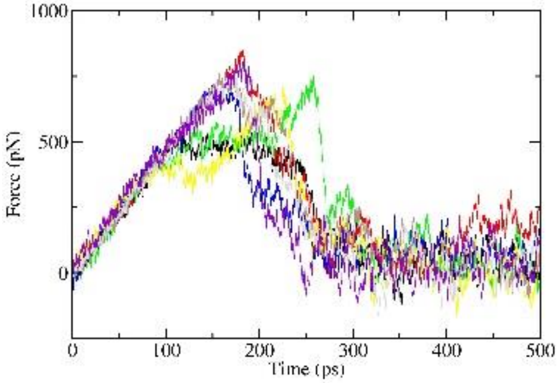
Force (pN)
Time (ps)
1000
500
0
0
100
200
300
400
500

Calpain inhibitor I	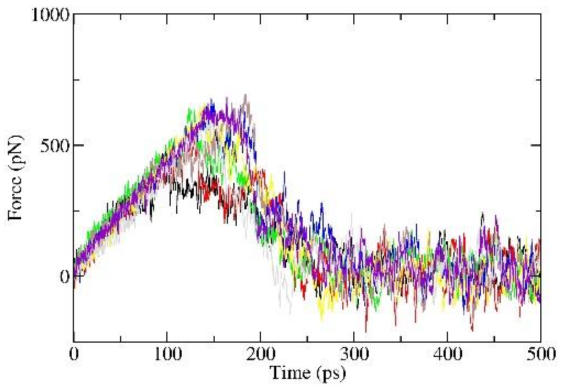	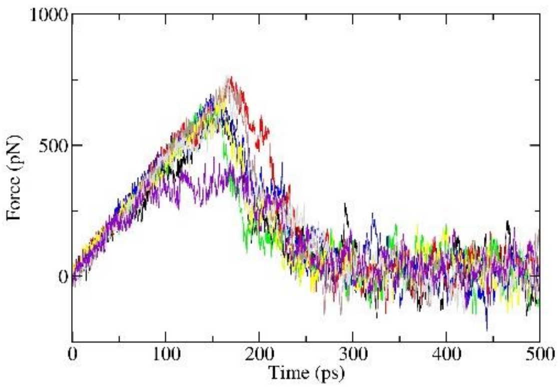
Calpain inhibitor II	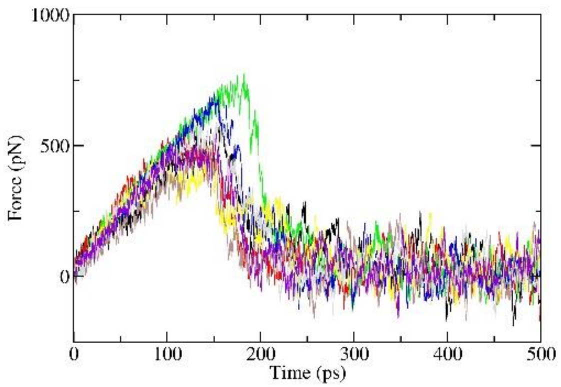	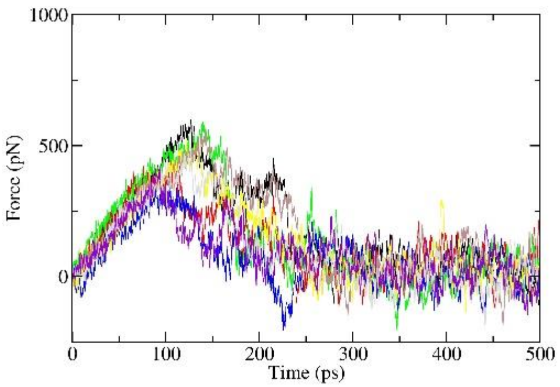

Calpain inhibitor XII	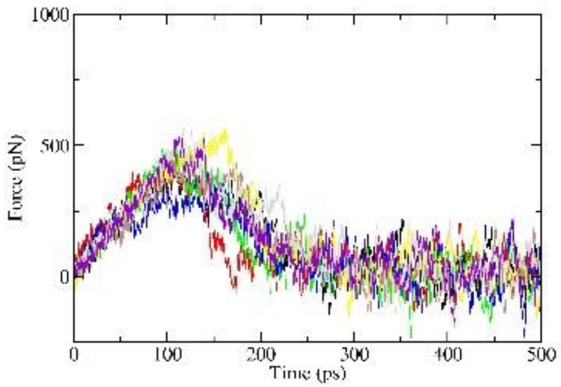	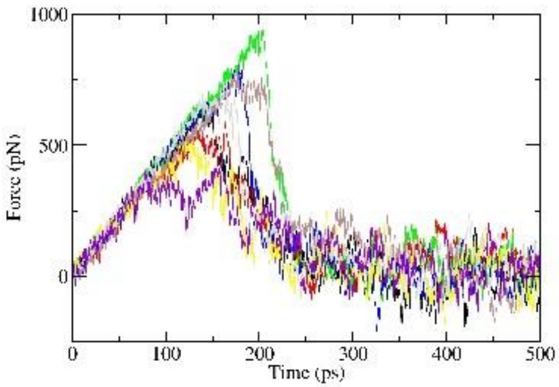
Calpeptin	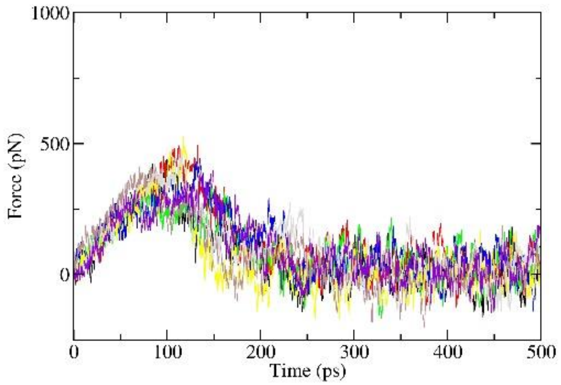	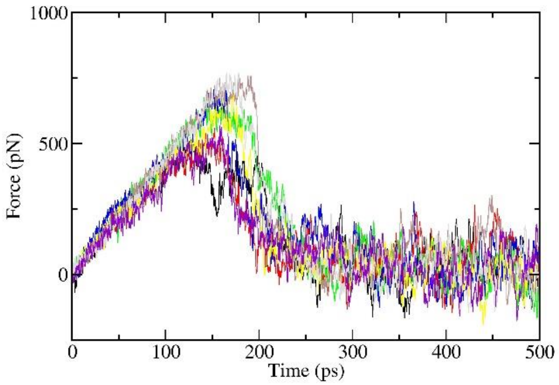

Candesartan cilexetil	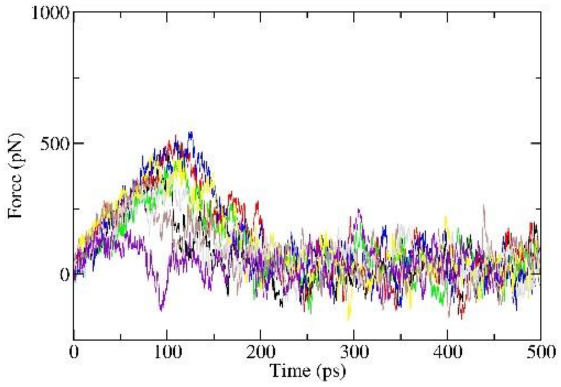	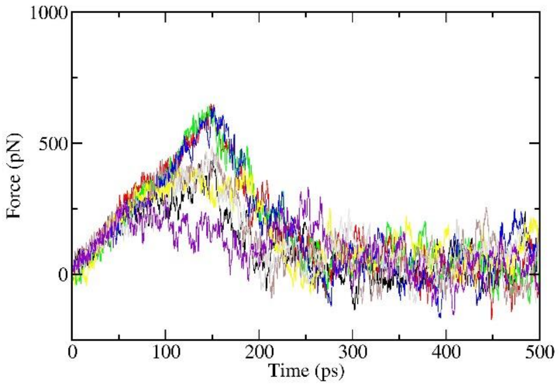
Carmofur	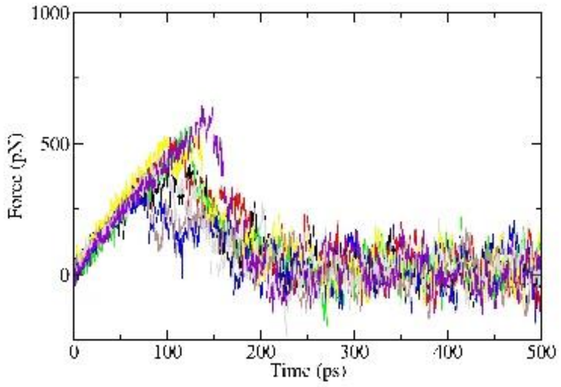	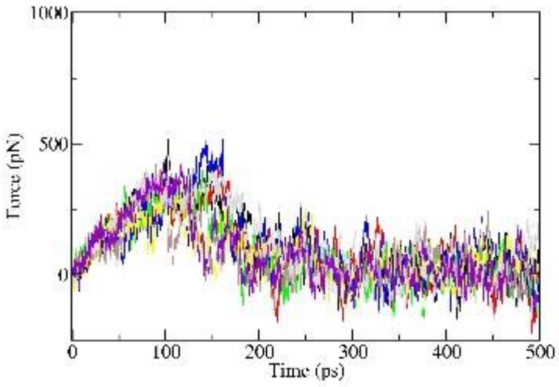

Chloroquine	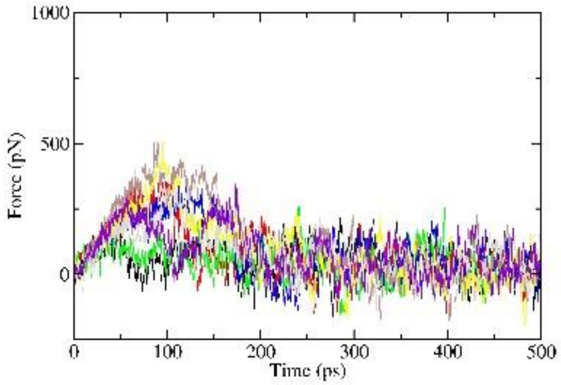	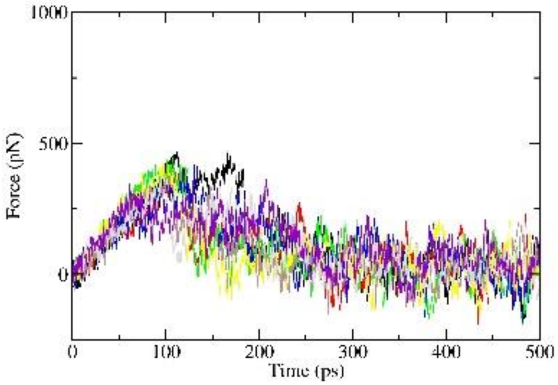
Dypyradimole	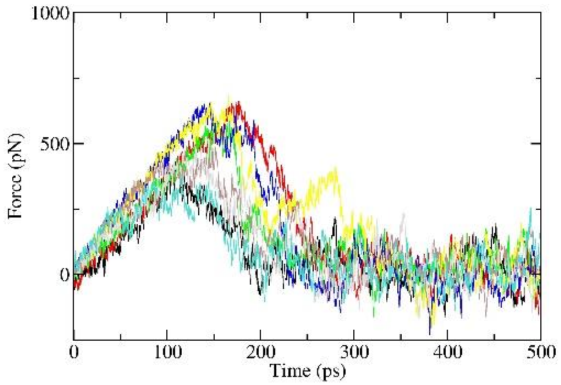	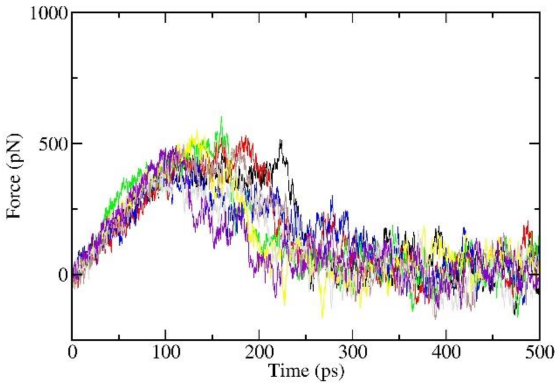

Disulfiram	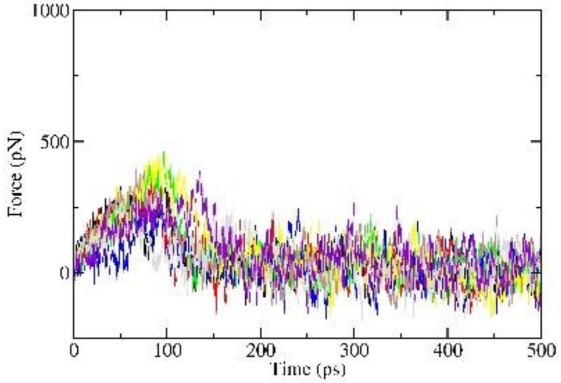	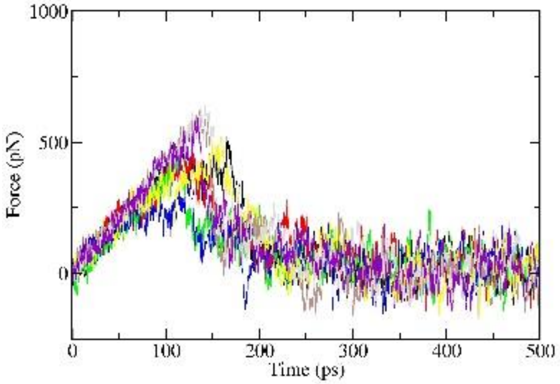
GC-373	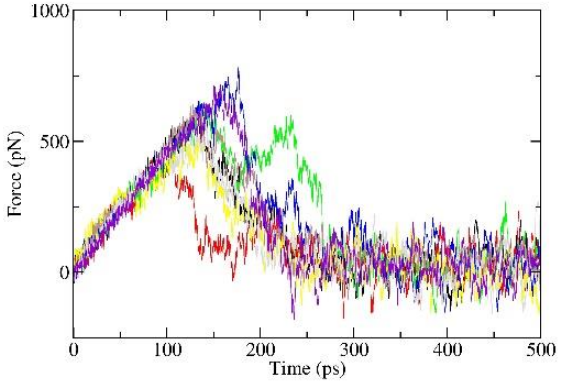	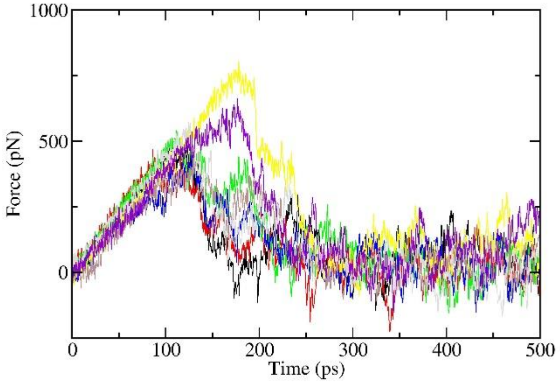

Hydroxychloroquine	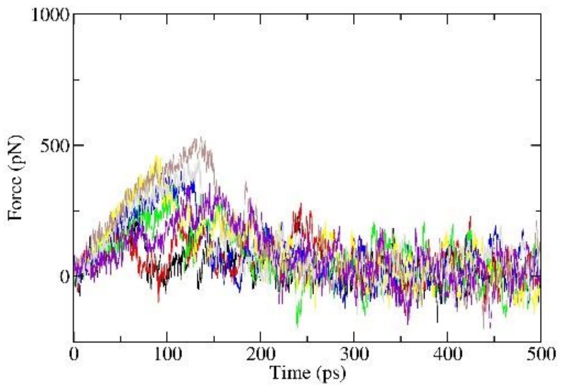	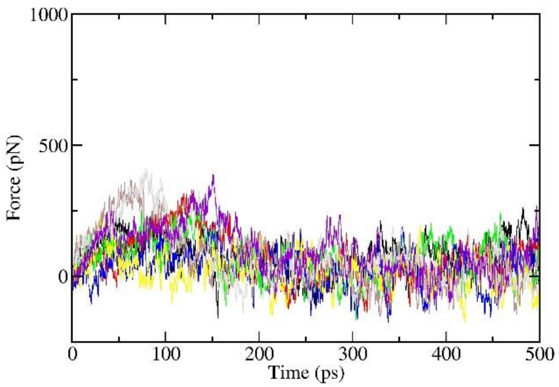
MG-115	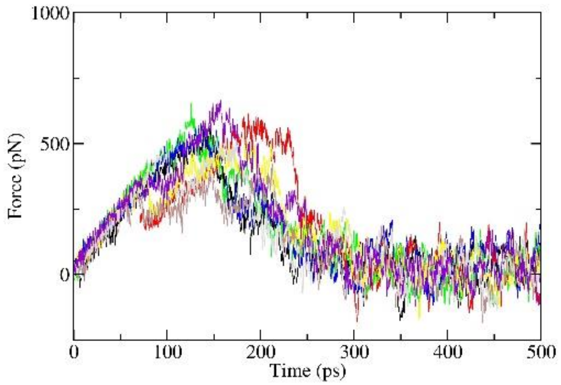	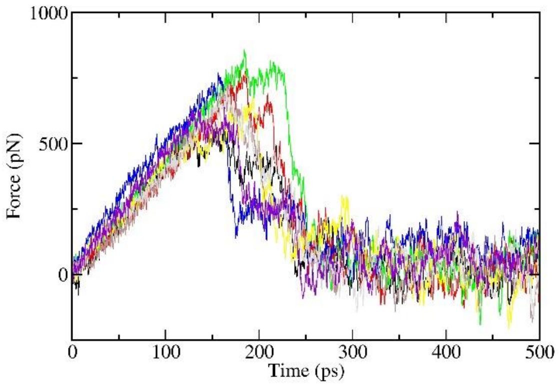

<table>
<tr>
<td>MG-132</td>
<td>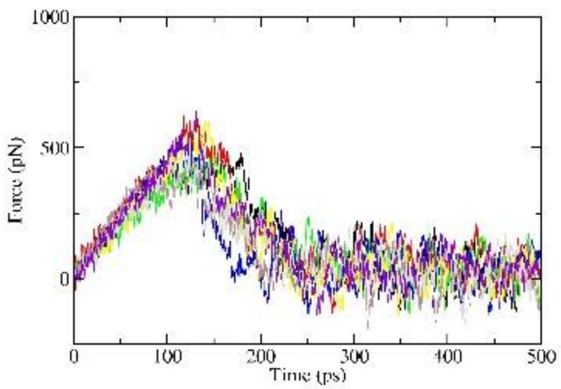
</td>
<td>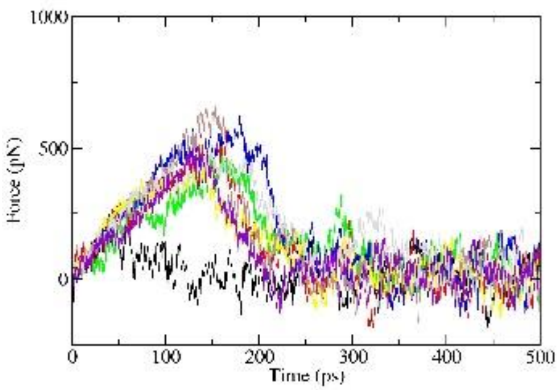
</td>
</tr>
<tr>
<td>Narlaprevir</td>
<td>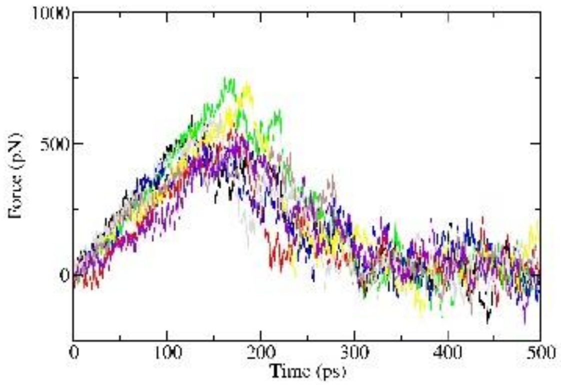
</td>
<td>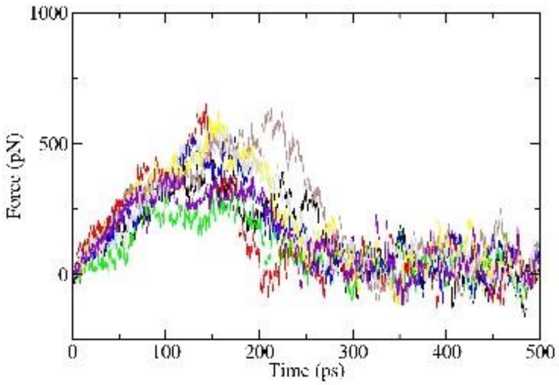
</td>
</tr>
</table>

Omeprazole	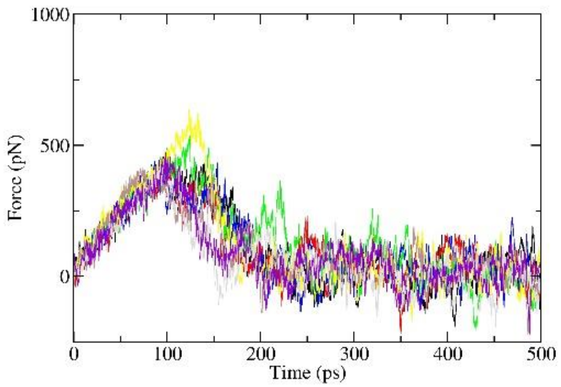	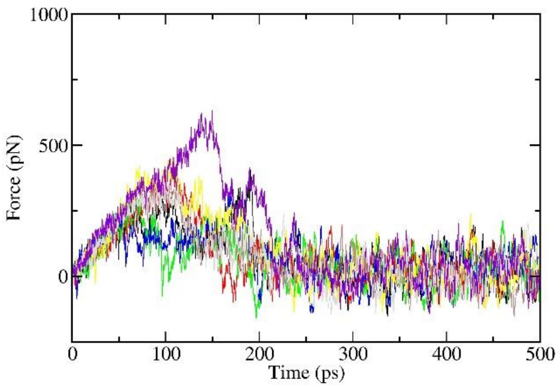
Oxytetracycline	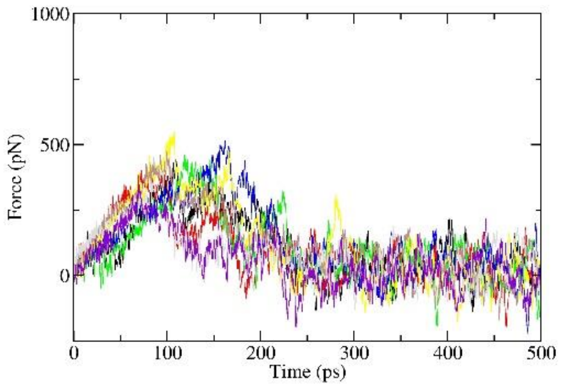	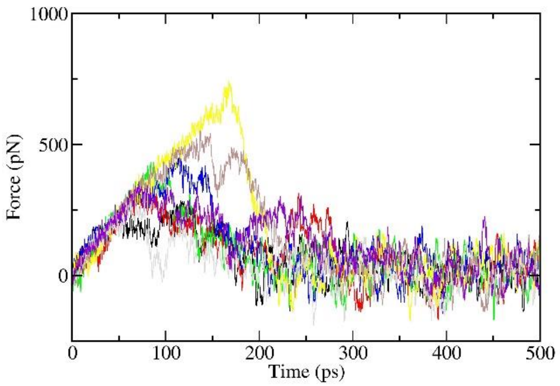

PX-12	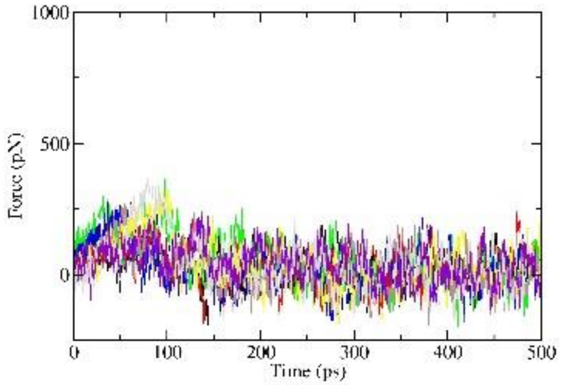	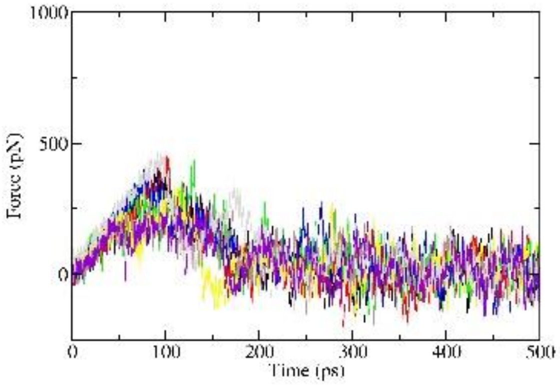
Shikonin	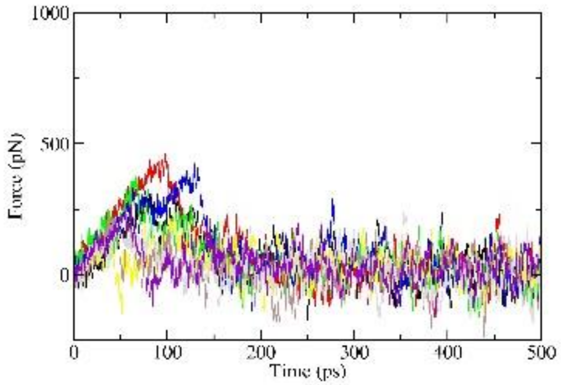	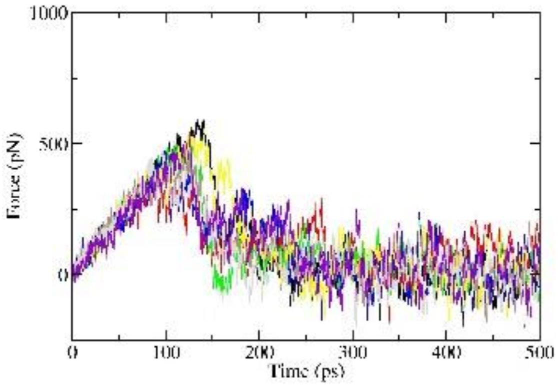

Table S3. The Pulling Work in Time Dependence over 8 Independent SMD Trajectories.

Name	Monomer	Dimer
7J	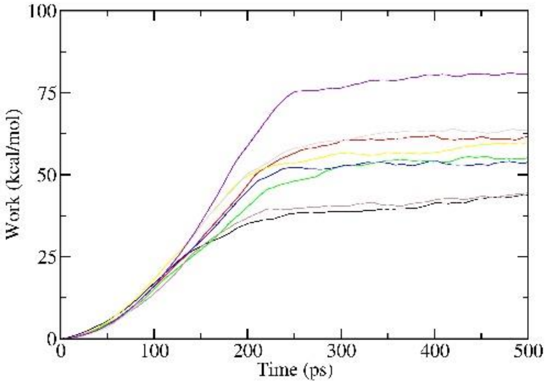	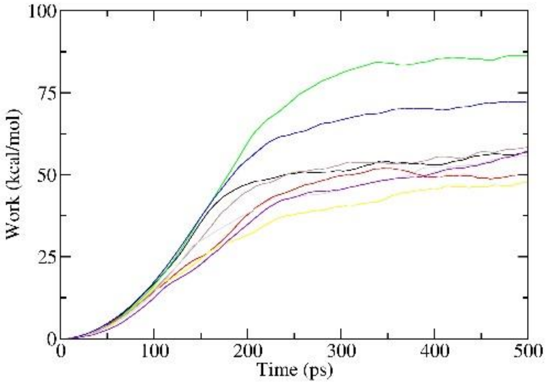
11a	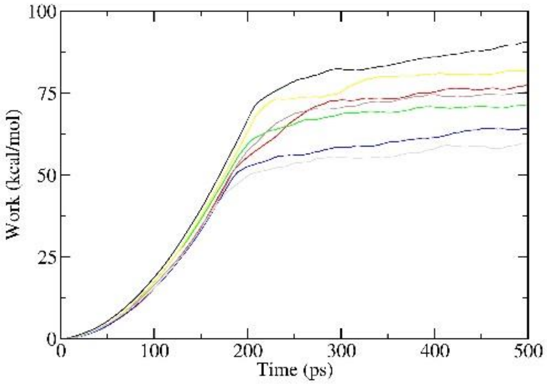	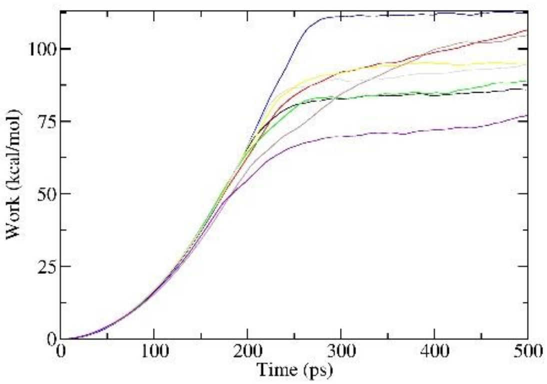

<table>
<tr>
<td>11b</td>
<td>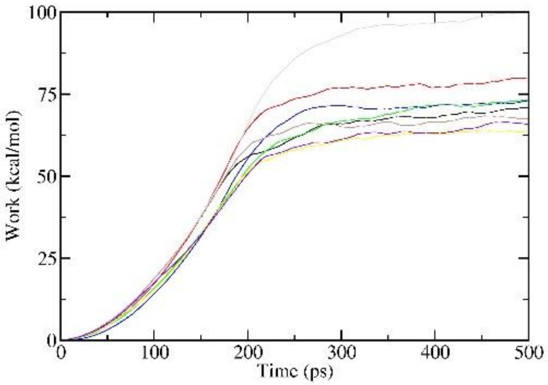
</td>
<td>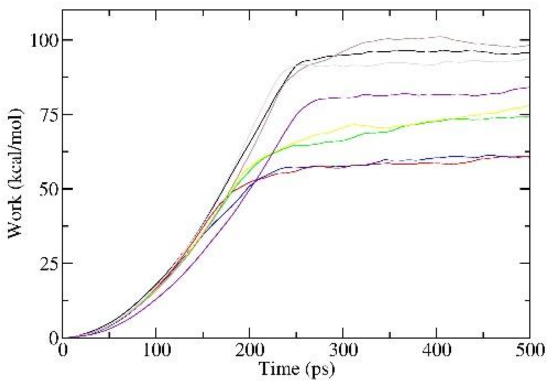
</td>
</tr>
<tr>
<td>11r</td>
<td>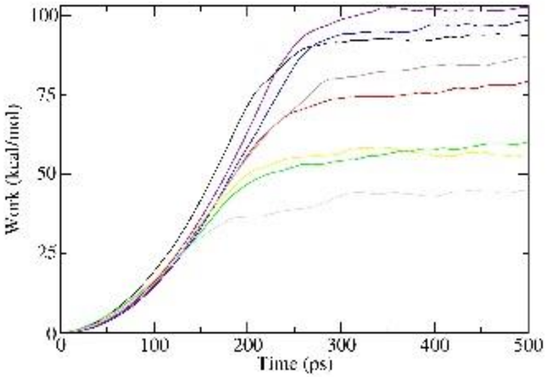
</td>
<td>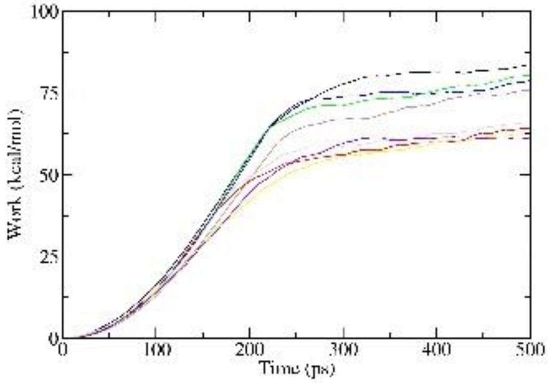
</td>
</tr>
</table>

13a	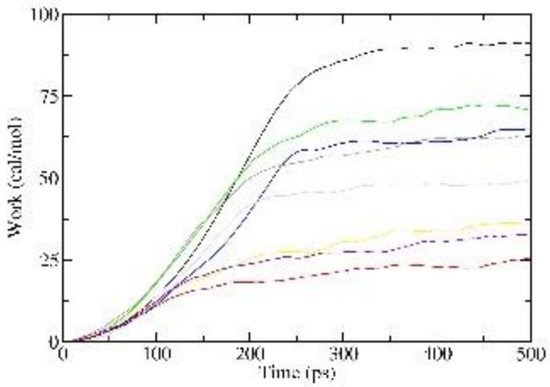Work (cal/mol) 0 25 50 75 100 Time (ps) 0 100 200 300 400 500	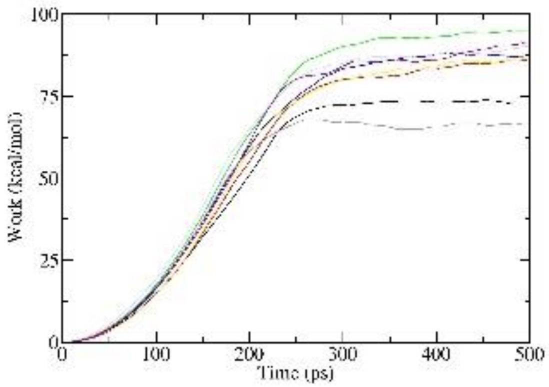Work (kcal/mol) 0 25 50 75 100 Time (ps) 0 100 200 300 400 500
13b	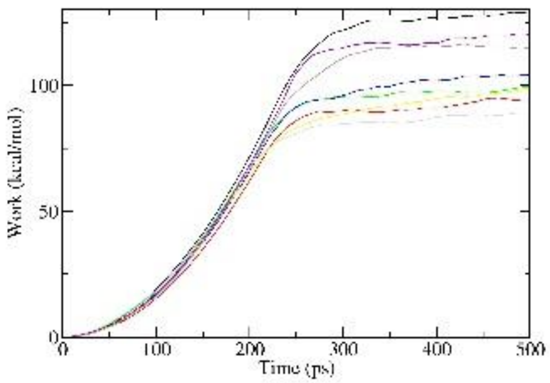Work (kcal/mol) 0 50 100 Time (ps) 0 100 200 300 400 500	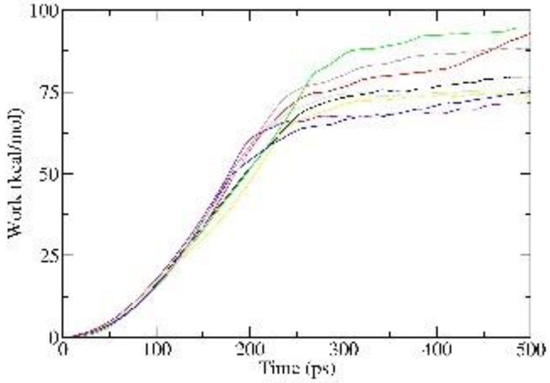Work (kcal/mol) 0 25 50 75 100 Time (ps) 0 100 200 300 400 500

Calpain inhibitor I	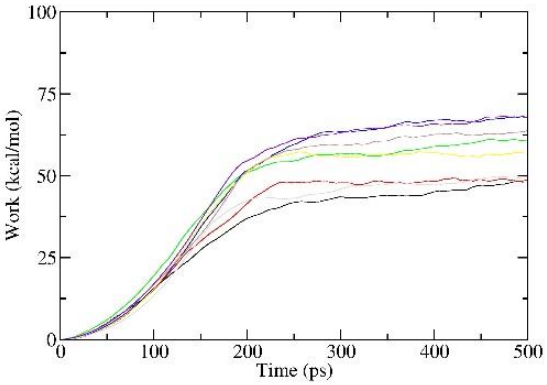	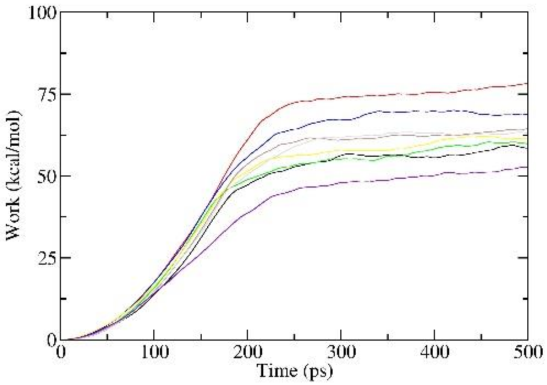
Calpain inhibitor II	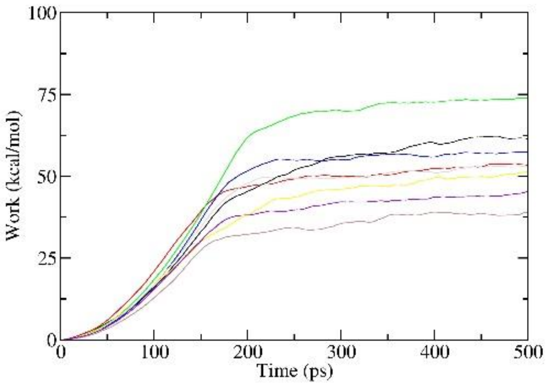	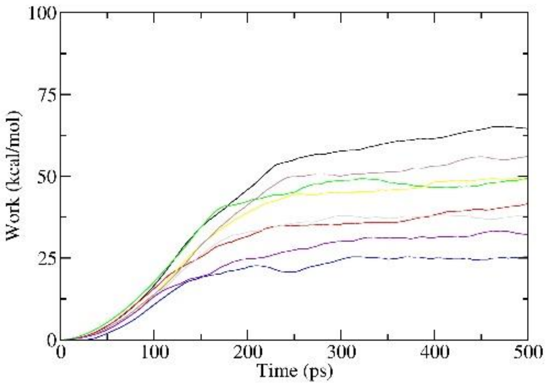

Calpain inhibitor XII	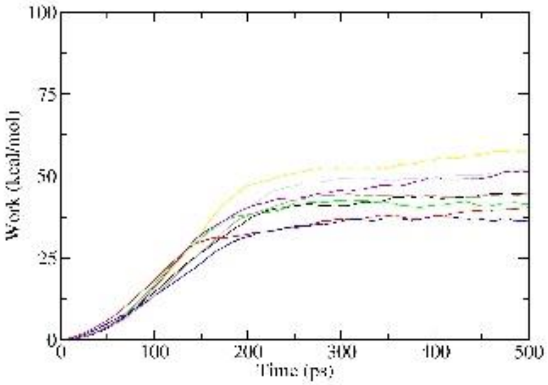	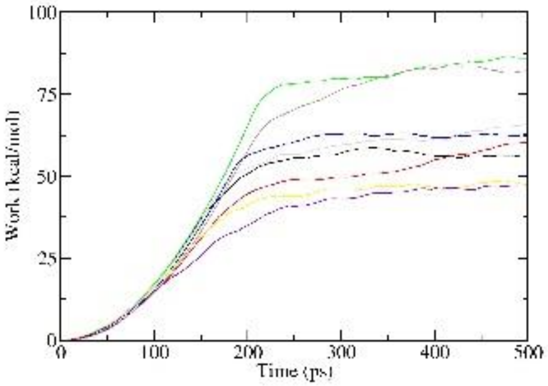
Calpeptin	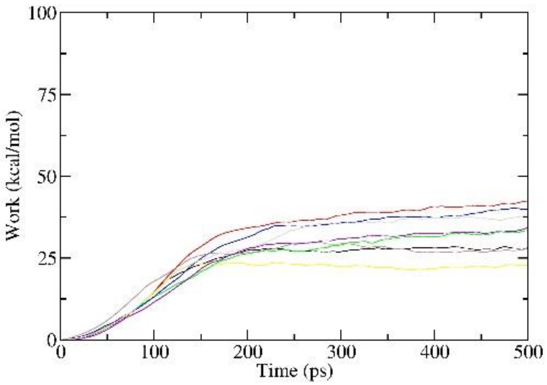	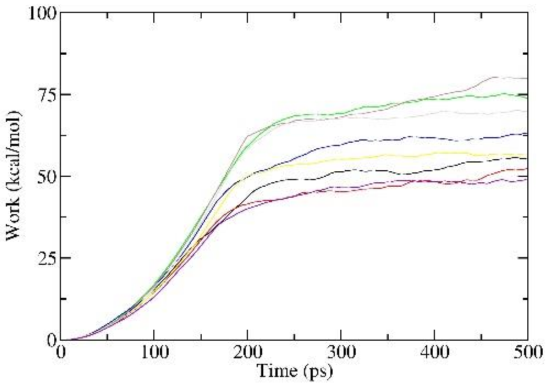

Candesartan cilexetil	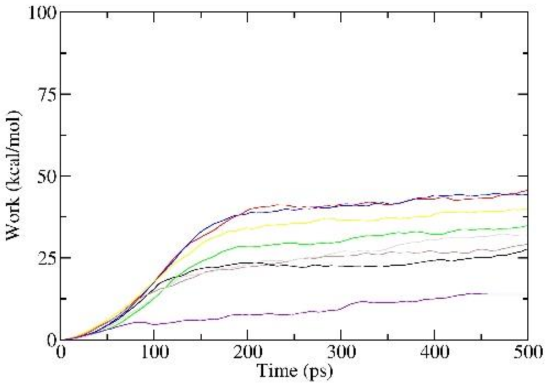	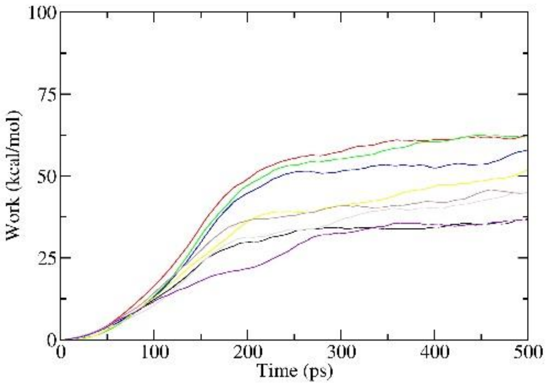
Carmofur	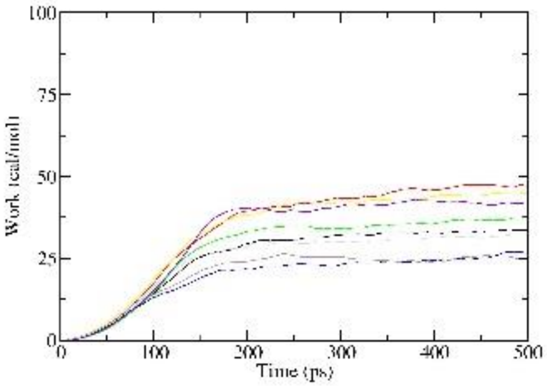	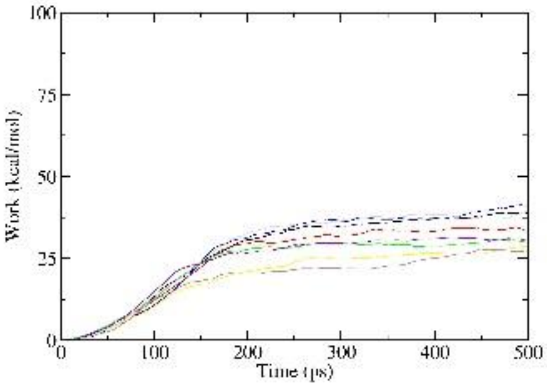

Chloroquine	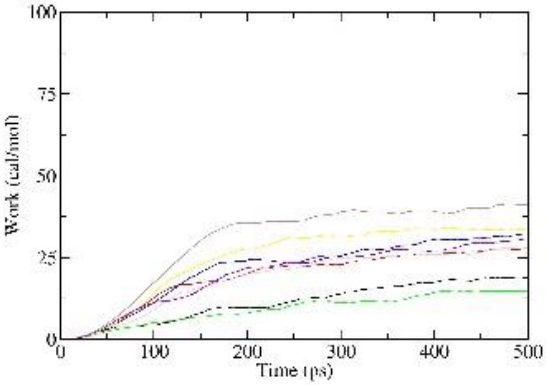	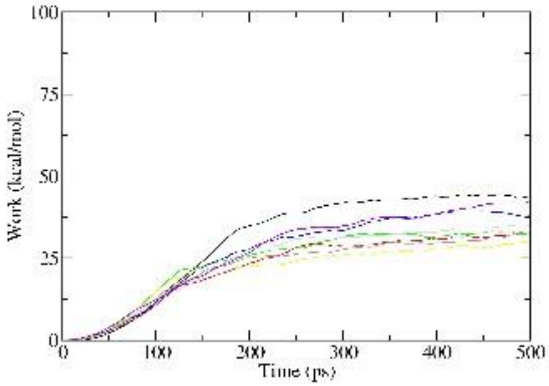
Dipyradimole	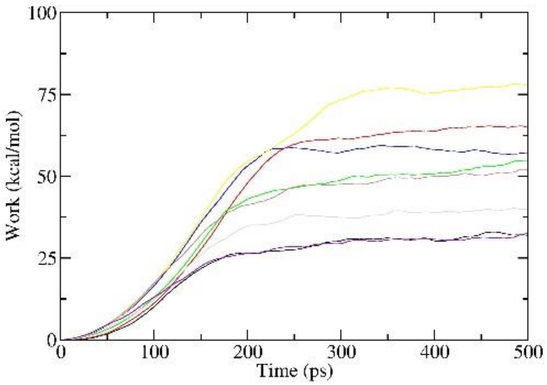	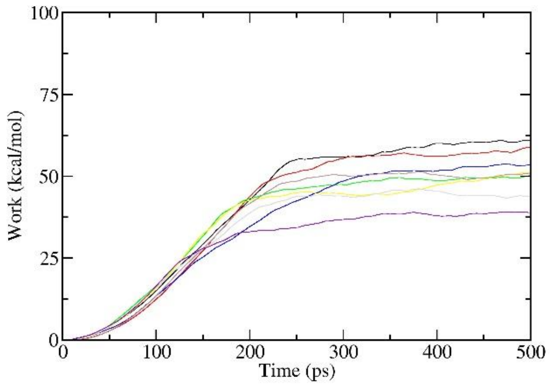

Disulfiram	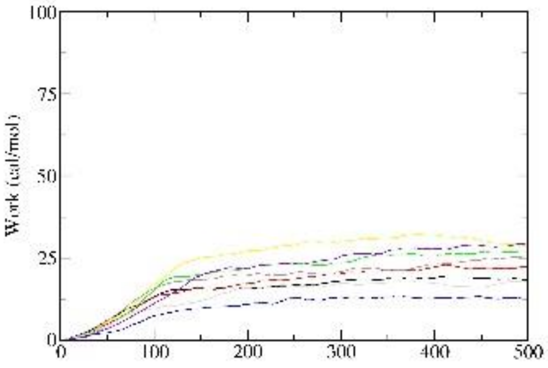	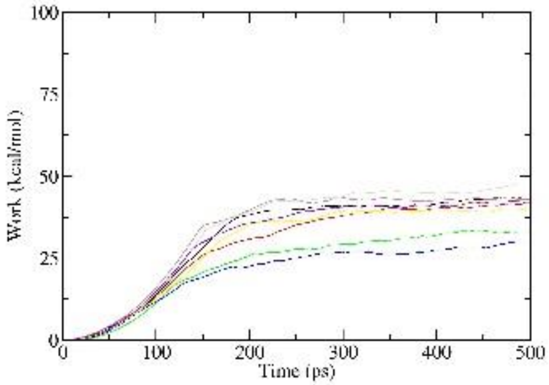
GC-373	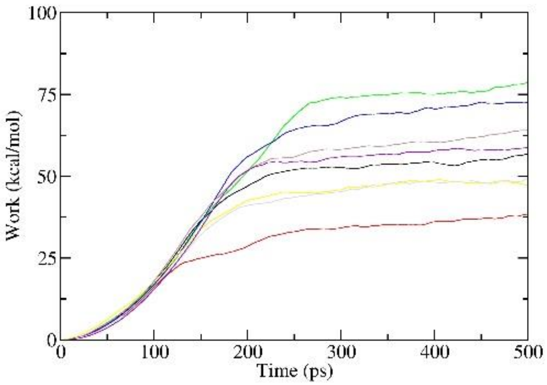	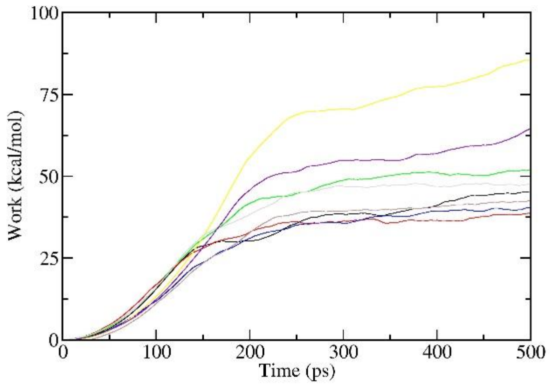

Hydroxychloroquine	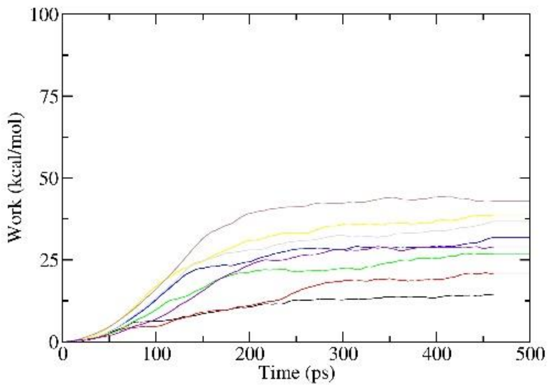	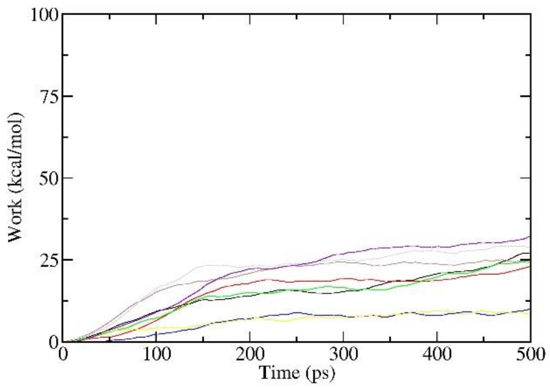
MG-115	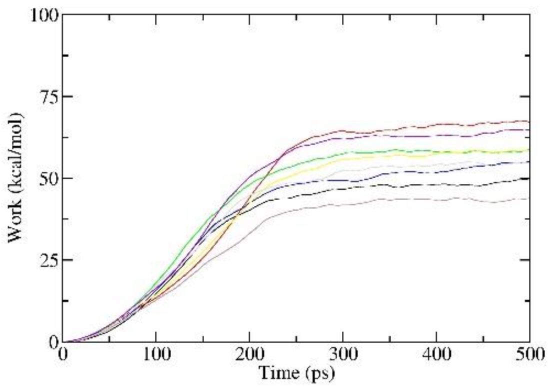	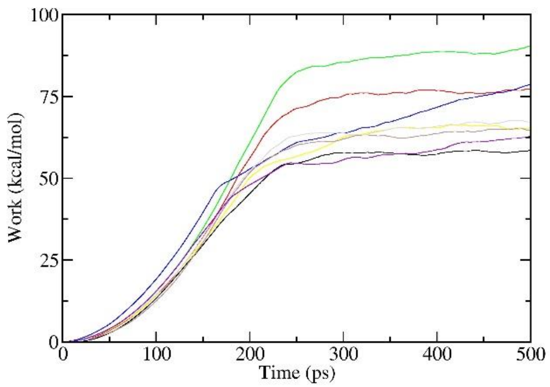

MG-132	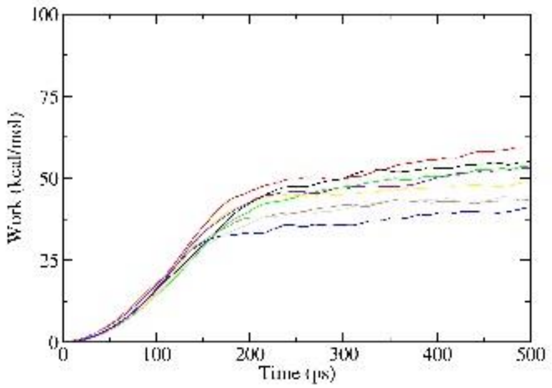	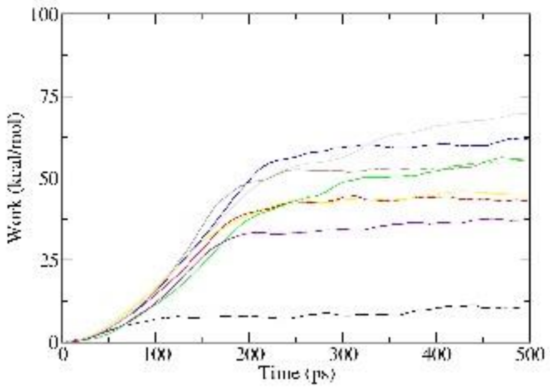
Narlaprevir	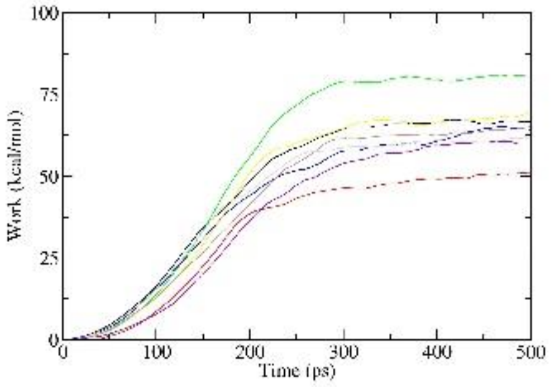	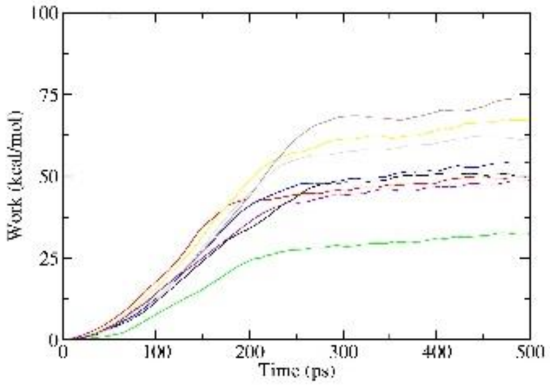

Omeprazole

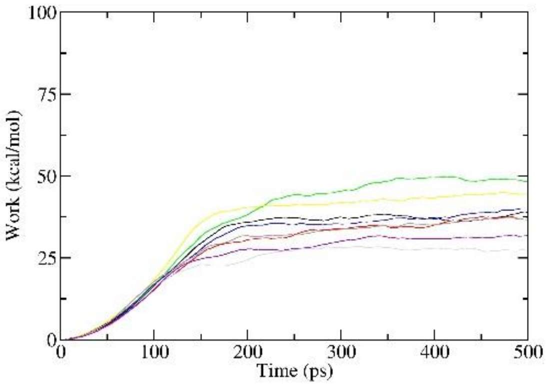

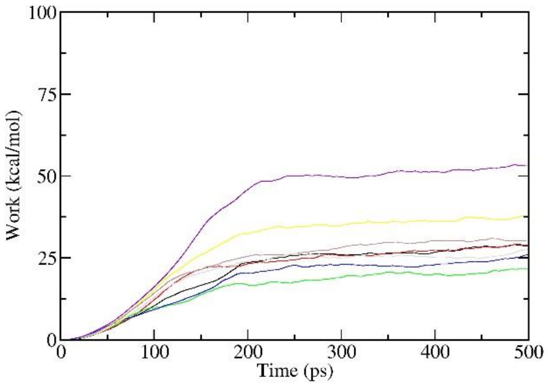

Oxytetracycline

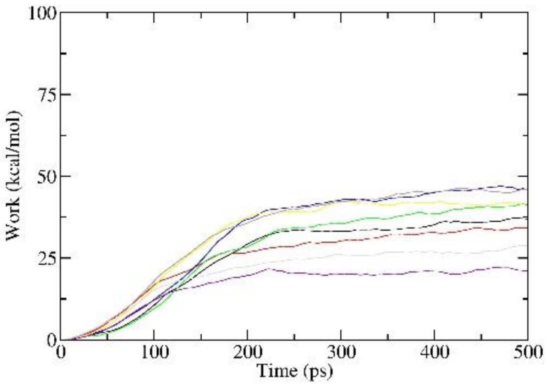

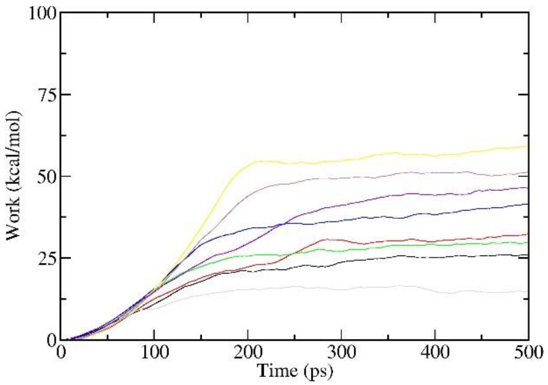

PX-12	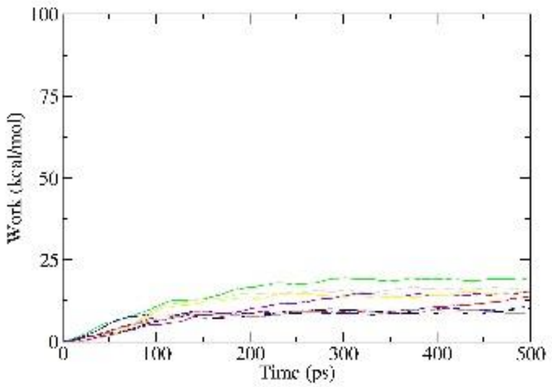	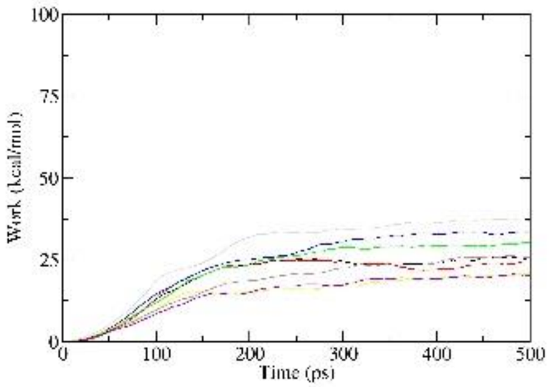
Shikonin	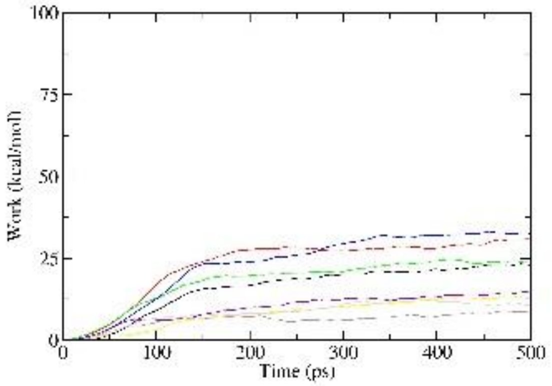	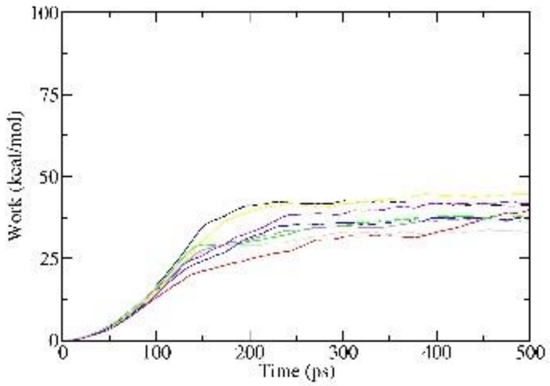

Table S4. Interaction diagram between inhibitors and SARS-CoV-2 Mpro via PLIP protocol.[1]

Name	Monomer	Dimer
7J	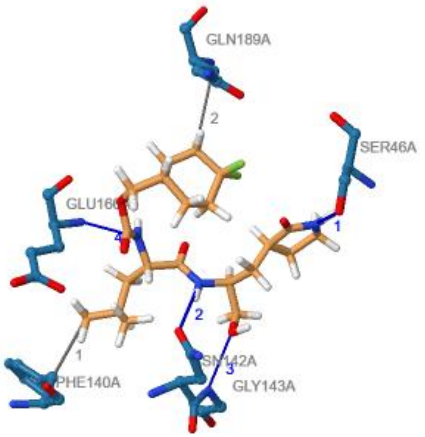	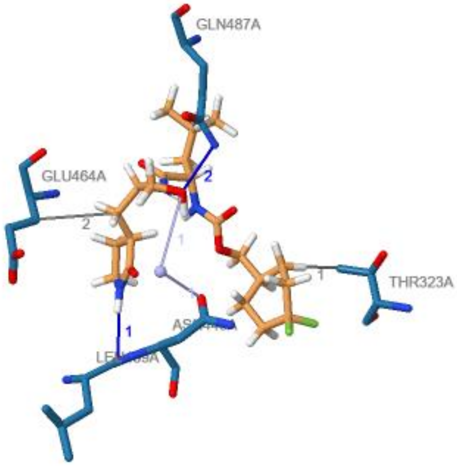
11a	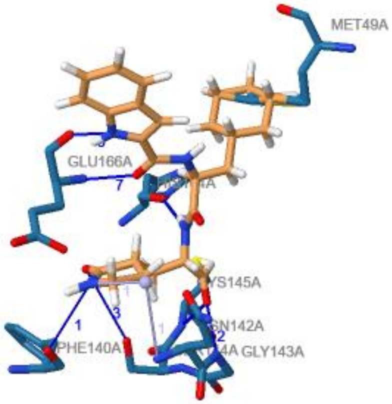	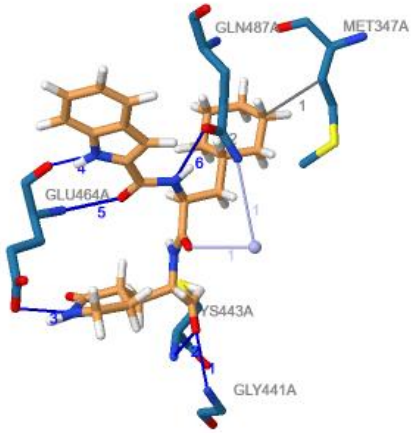

<table>
<tr>
<td>11b</td>
<td>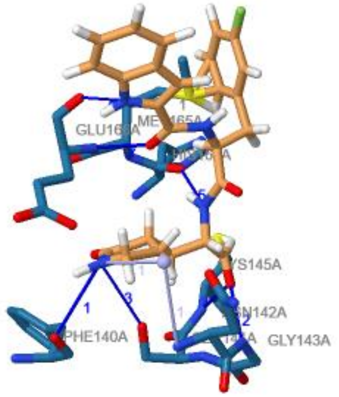
</td>
<td>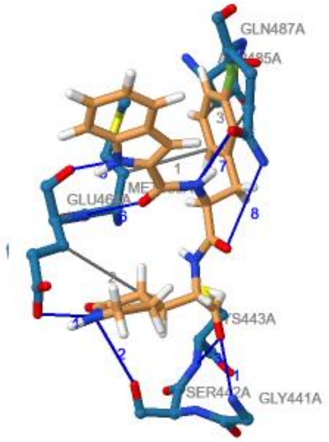
</td>
</tr>
<tr>
<td>11r</td>
<td>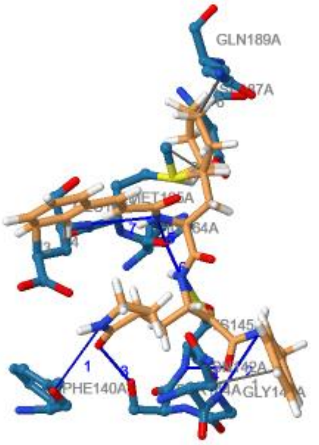
</td>
<td>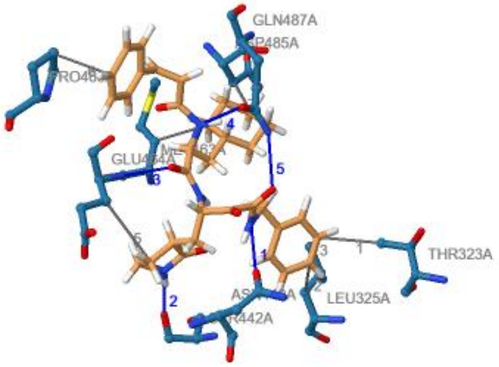
</td>
</tr>
</table>

<table>
<tr><td>13a</td><td>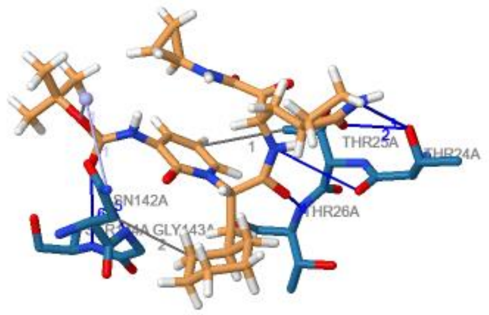
</td><td>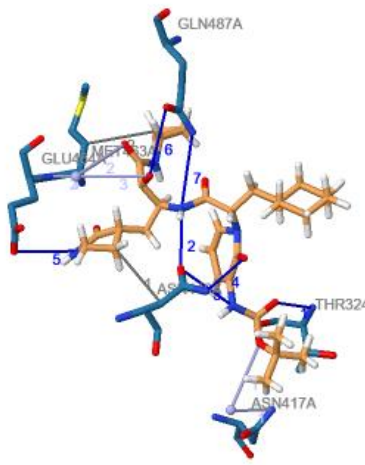
</td></tr>
<tr><td>13b</td><td>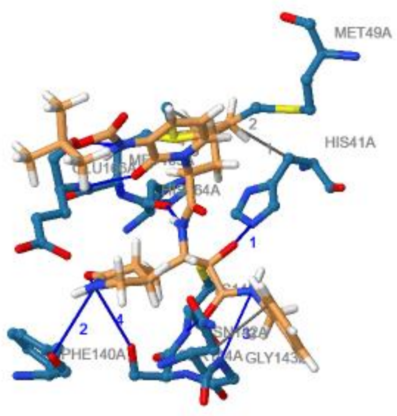
</td><td>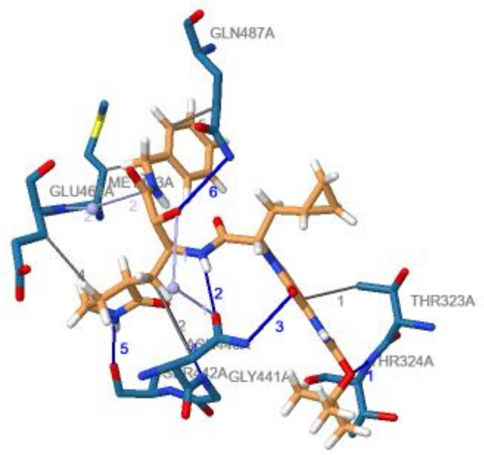
</td></tr>
</table>

<table>
<tr><td>Calpain inhibitor I</td><td>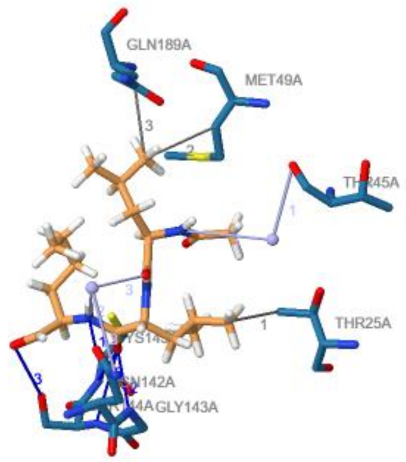
</td><td>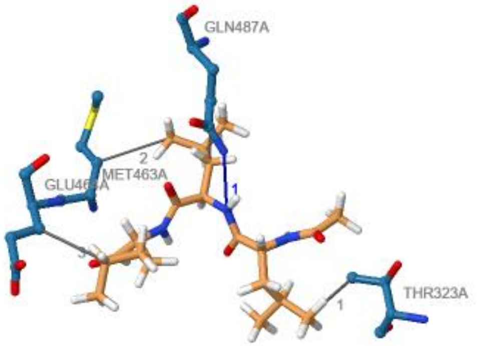
</td></tr>
<tr><td>Calpain inhibitor II</td><td>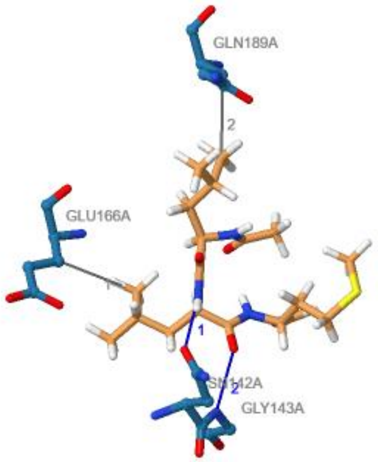
</td><td>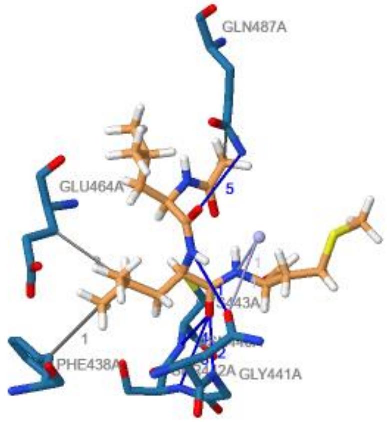
</td></tr>
</table>

<table>
<tr><td>Calpain inhibitor XII</td><td>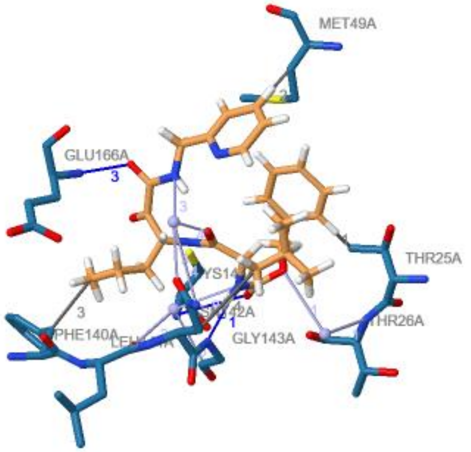
</td><td>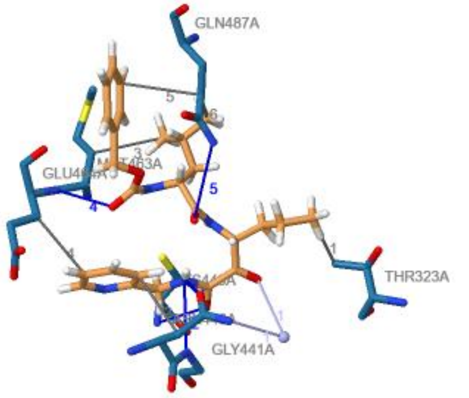
</td></tr>
<tr><td>Calpeptin</td><td>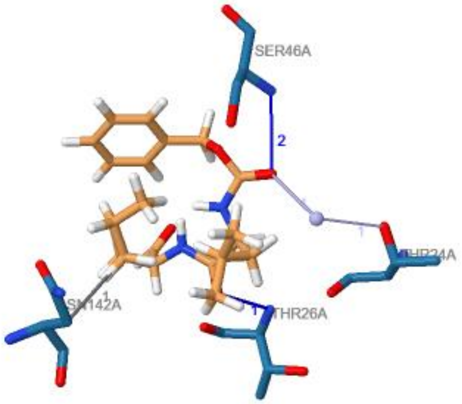
</td><td>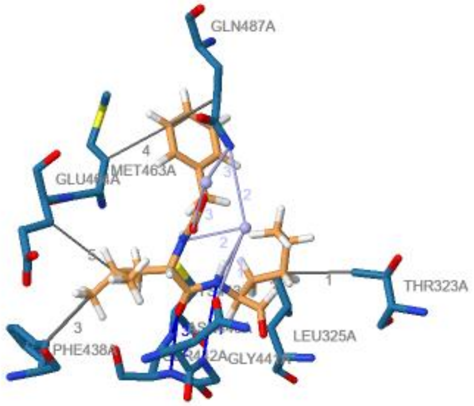
</td></tr>
</table>

<table>
<tr>
<td>Candesartan cilexetil</td>
<td>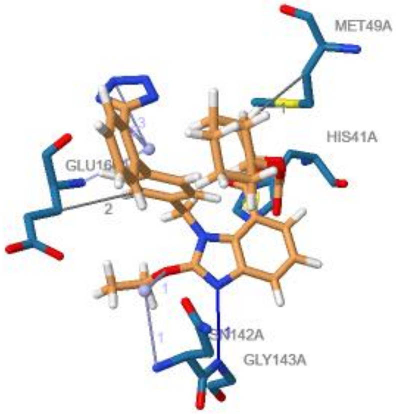
</td>
<td>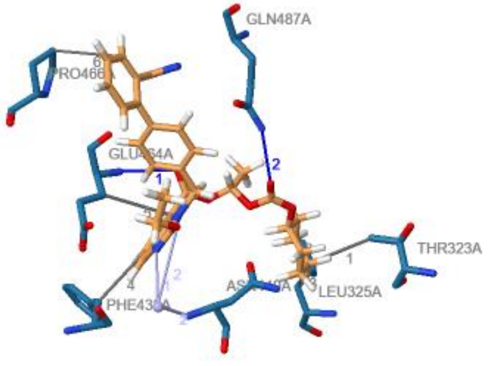
</td>
</tr>
<tr>
<td>Carmofur</td>
<td>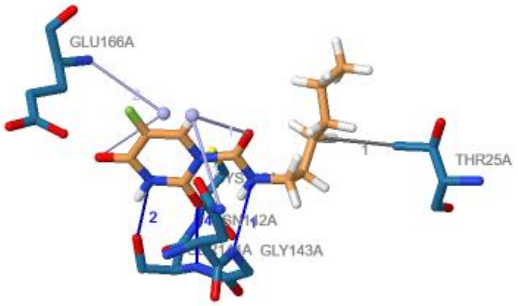
</td>
<td>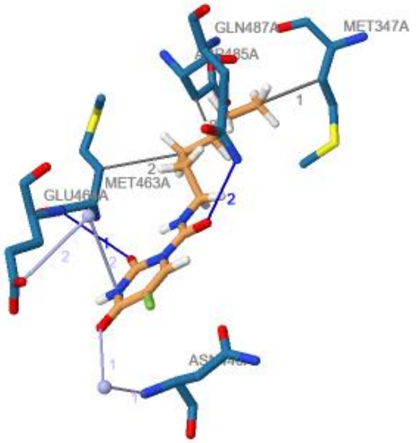
</td>
</tr>
</table>

Chloroquine	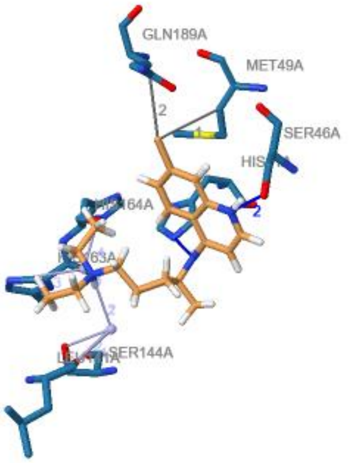	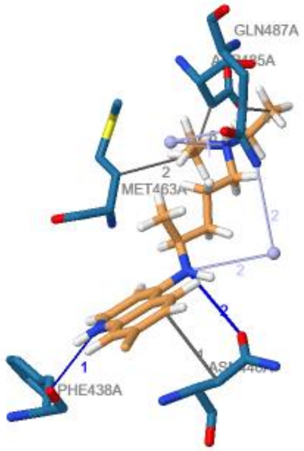
Dipyridamole	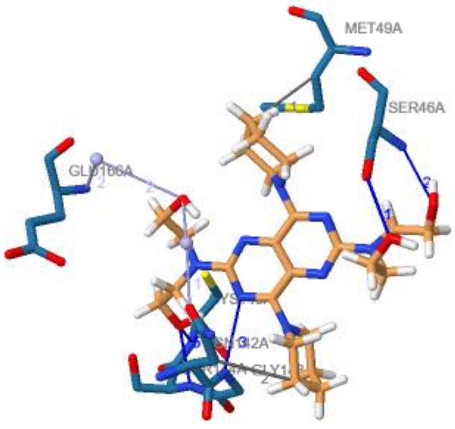	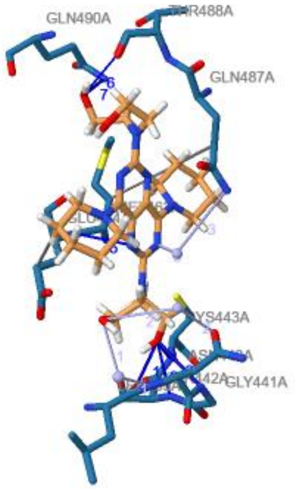
Disulfiram	N/A	N/A

GC-373	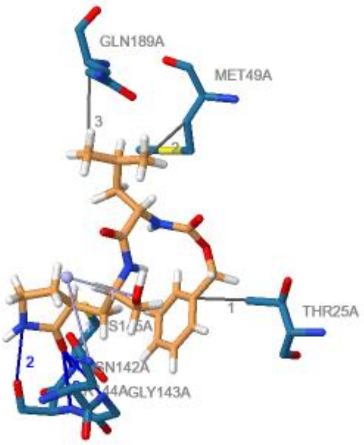	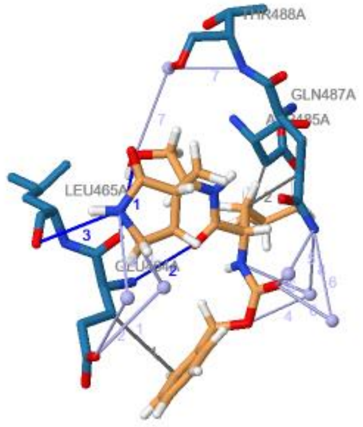
Hydroxychloroquine	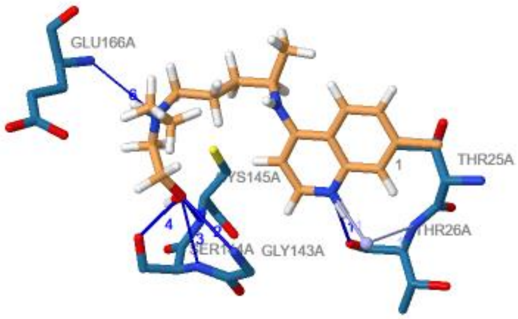	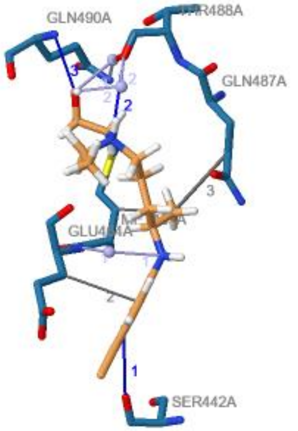

<table>
<tr>
<td>MG-115</td>
<td>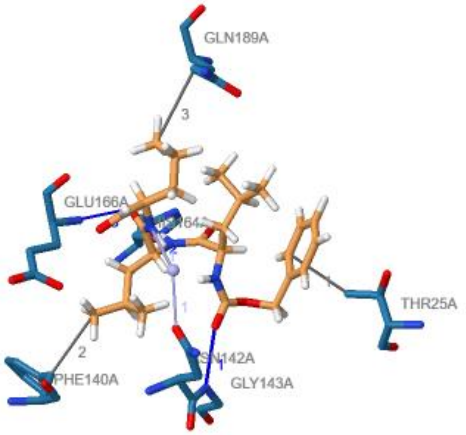
</td>
<td>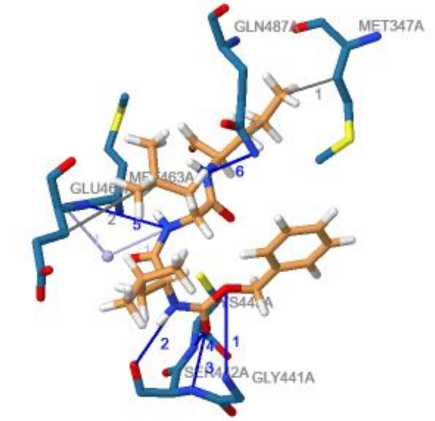
</td>
</tr>
<tr>
<td>MG-132</td>
<td>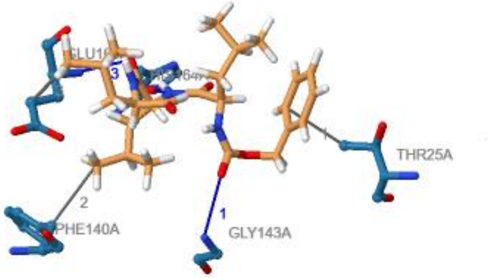
</td>
<td>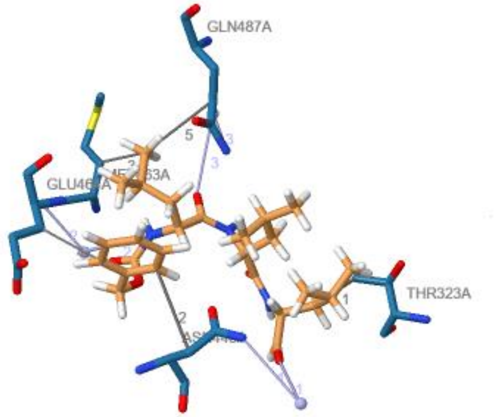
</td>
</tr>
</table>

Narlaprevir	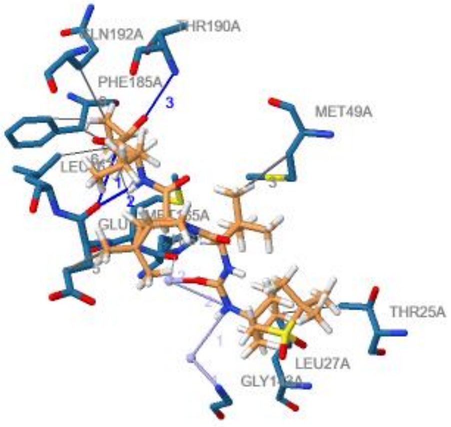	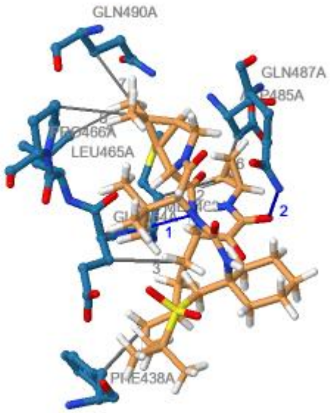
Omeprazole	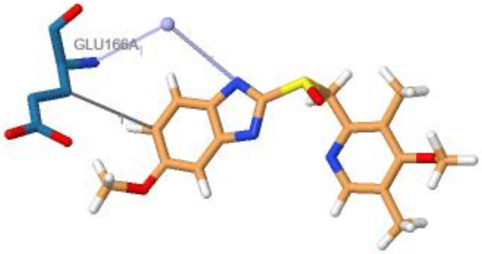	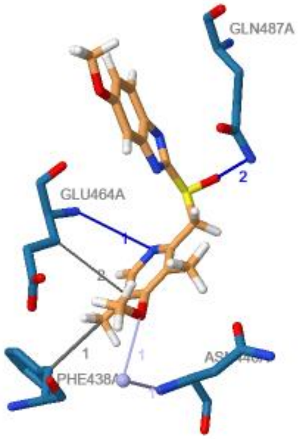

Oxytetracycline	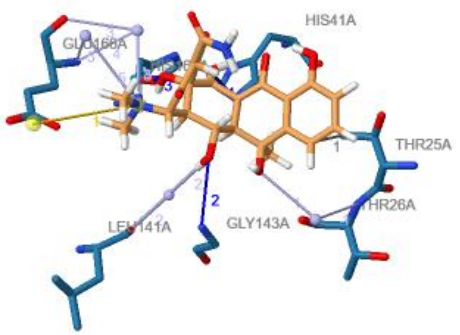	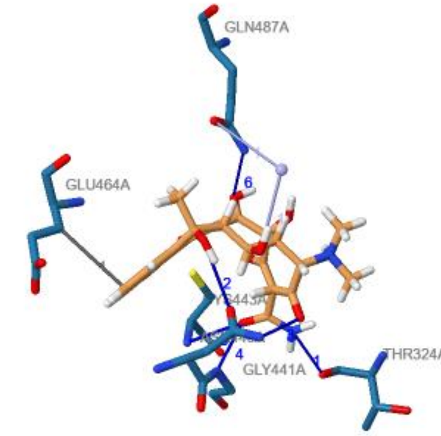
PX-12	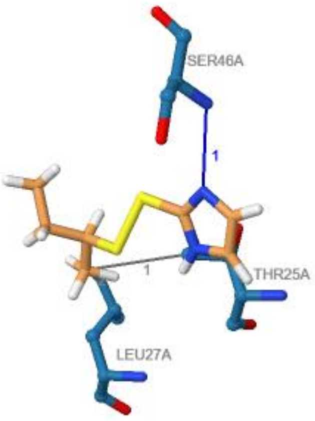	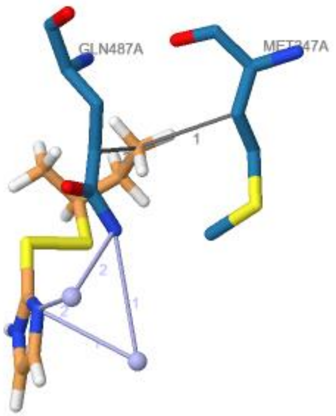

Shikonin	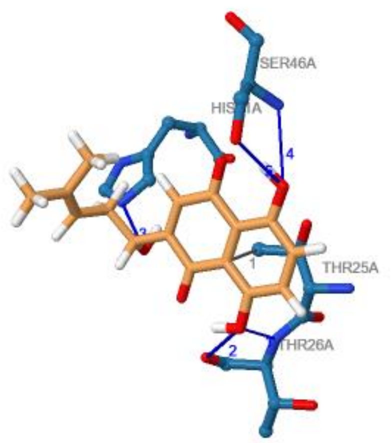	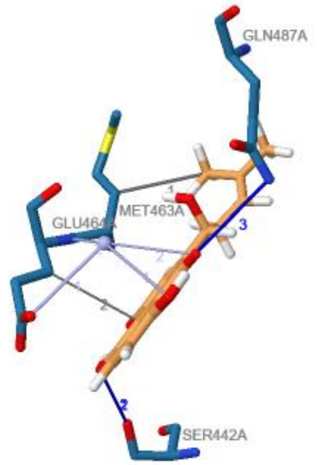

Table S5. The binding pose of other inhibitors to SARS-CoV-2 Mpro.

Name	Monomer	Dimer

Bazedoxifene	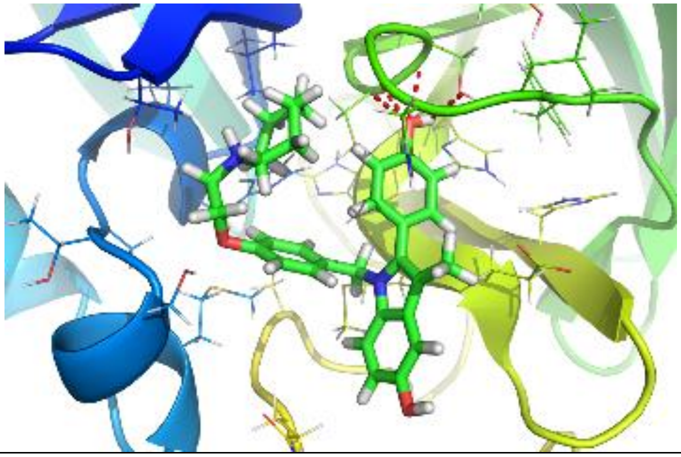	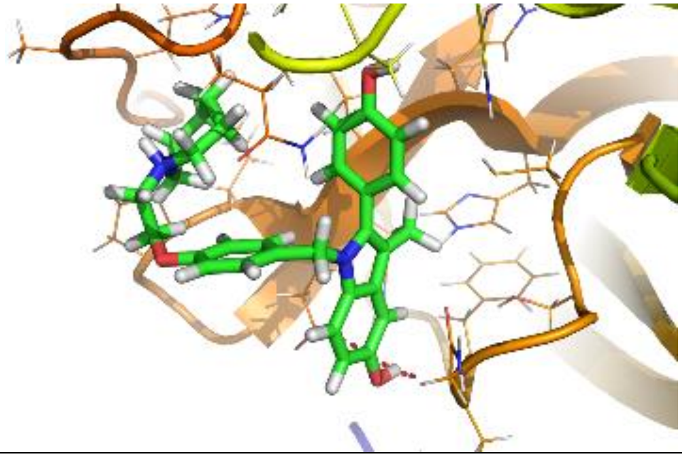
Cyclosporine	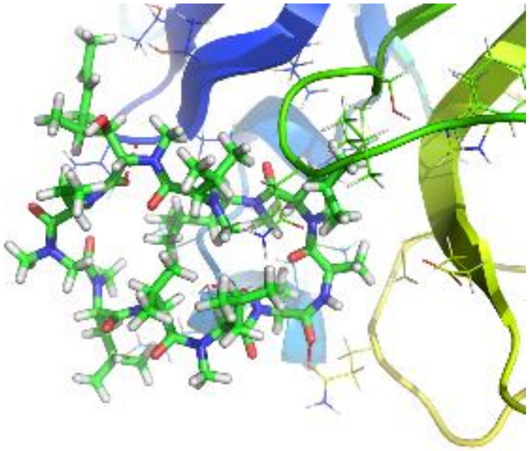	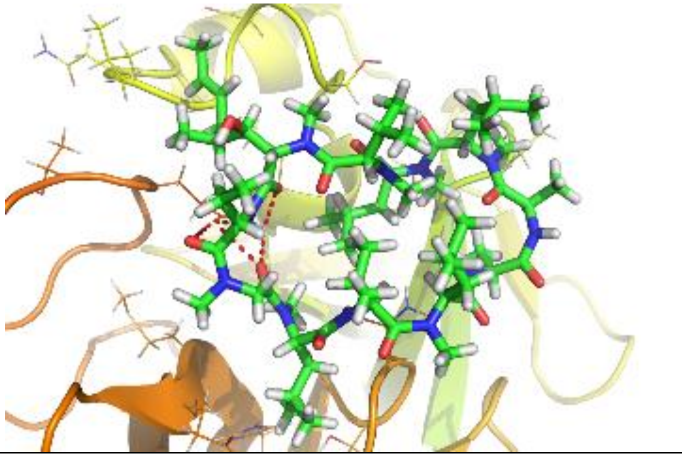

Digitoxin	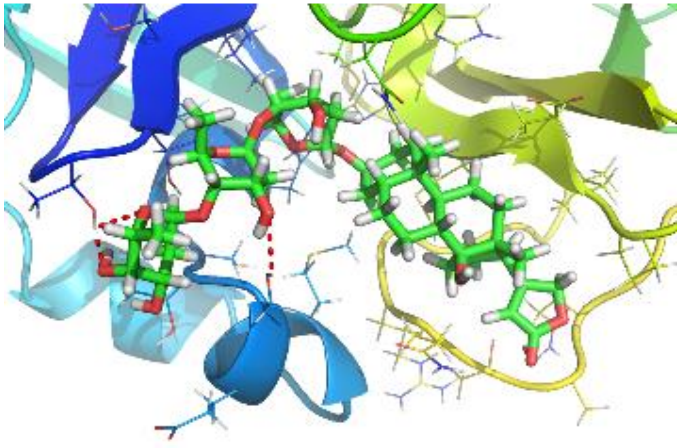	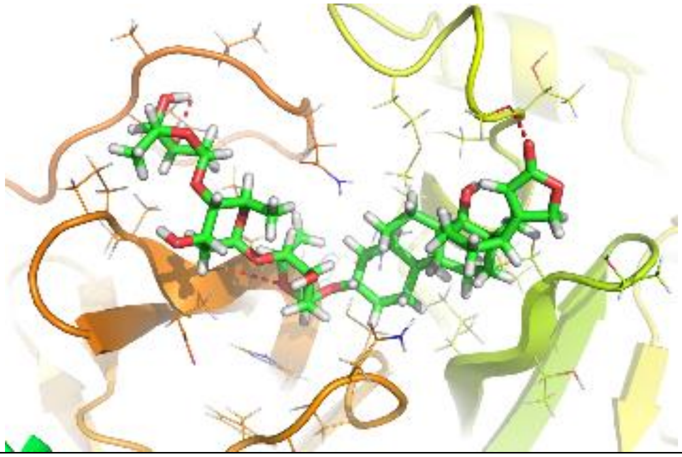
Digoxin	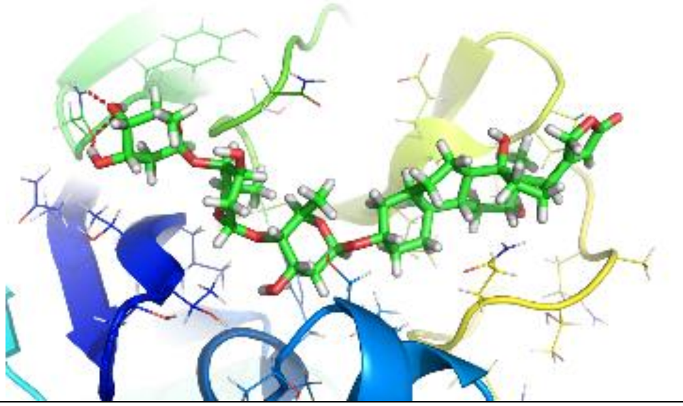	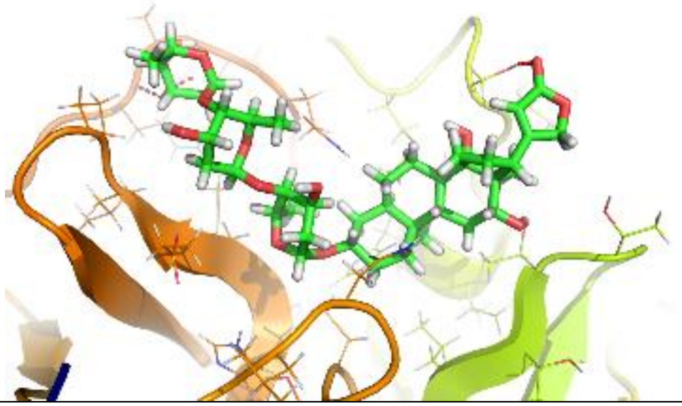

Dihydrogambogic Acid	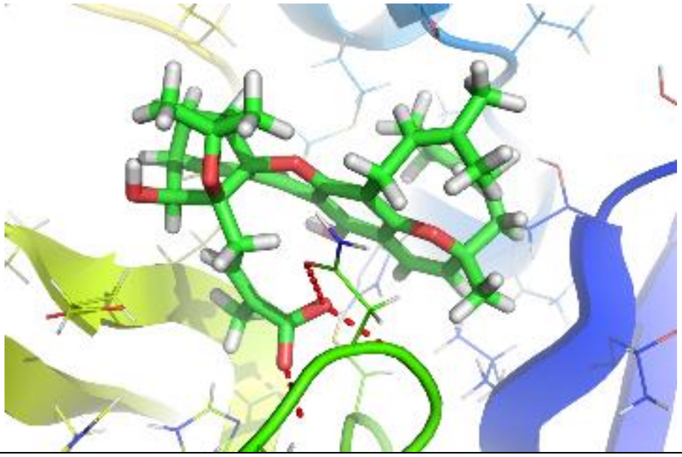	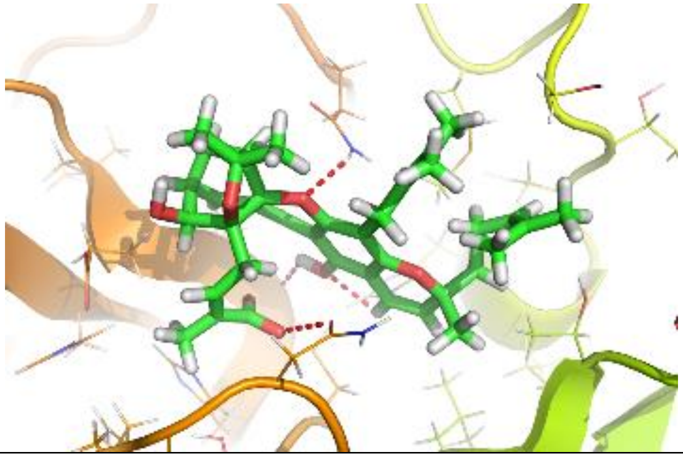
Ebastine	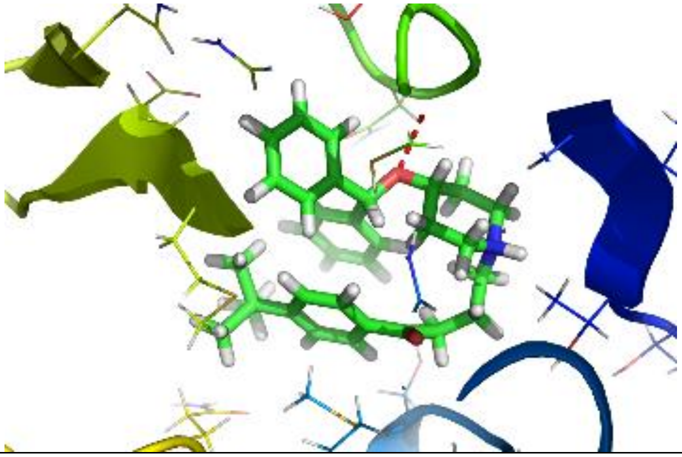	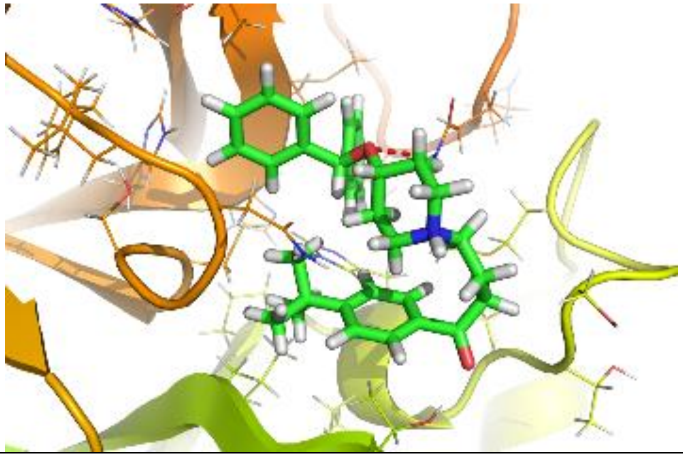

Favipiravir	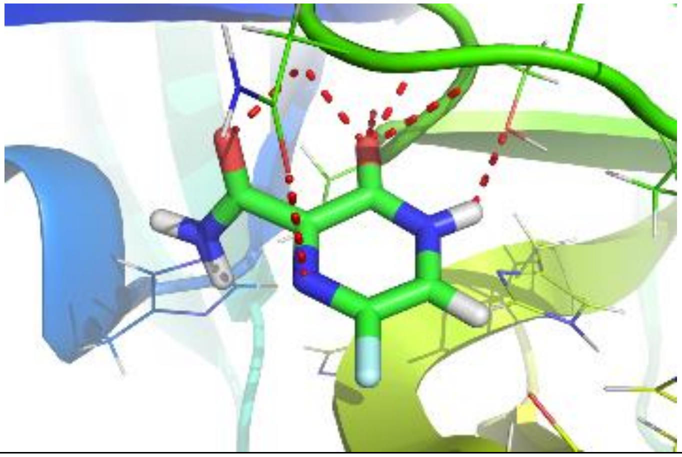	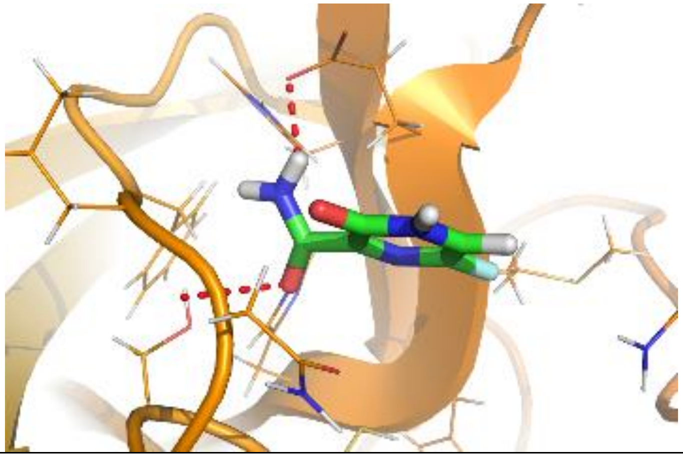
Fluspirilene	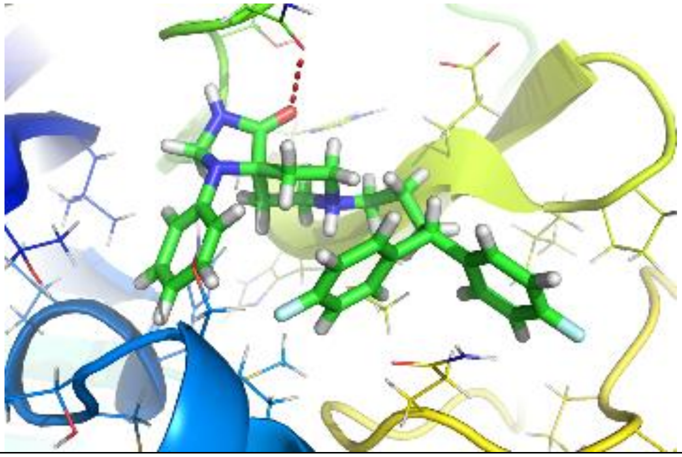	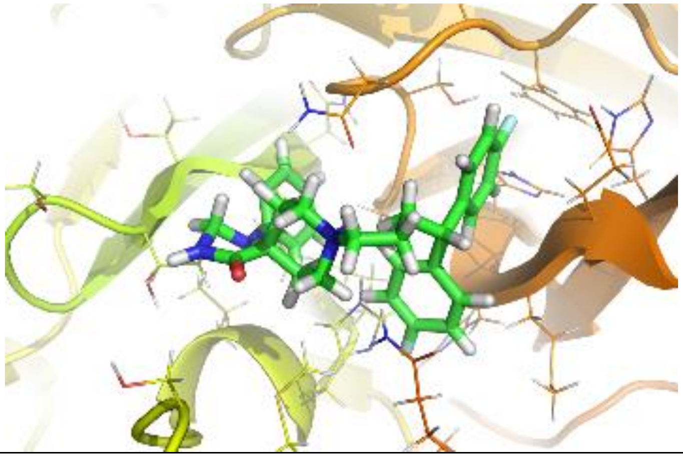

Isoosajin	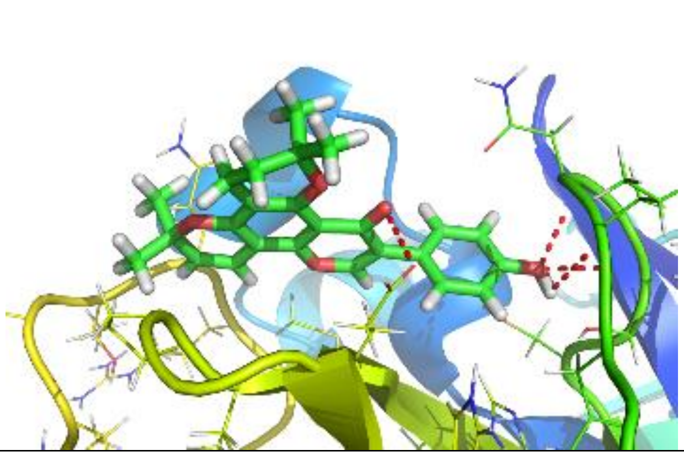	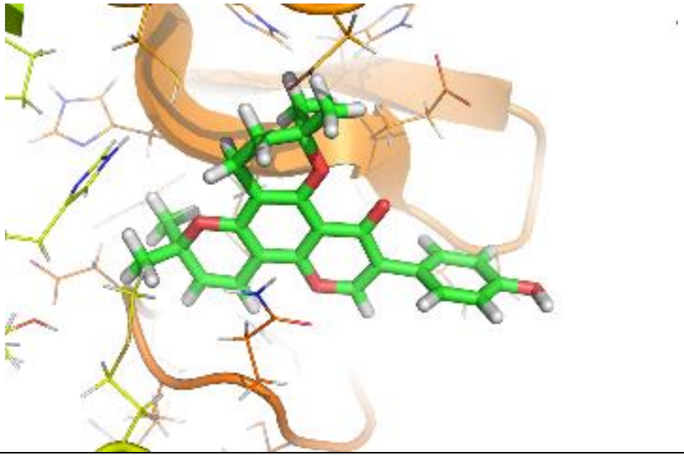
Ivacaftor	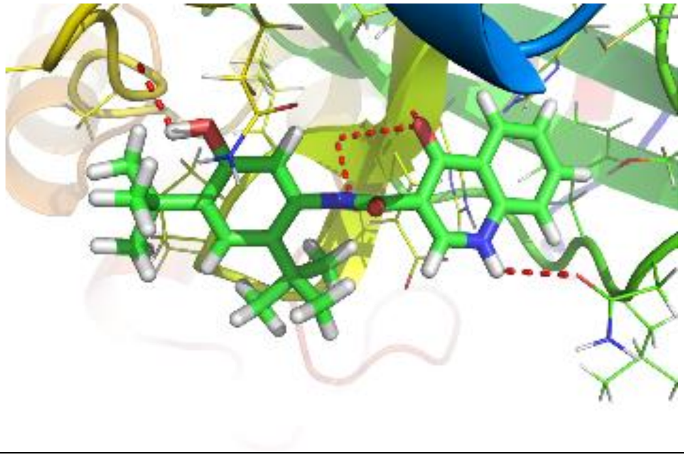	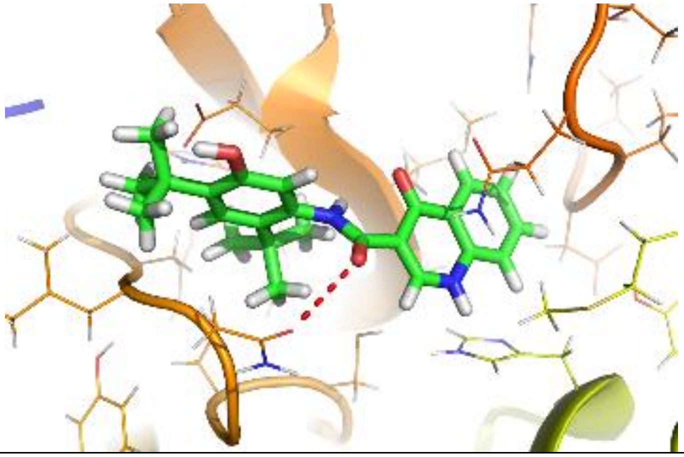

Lusutrombopag	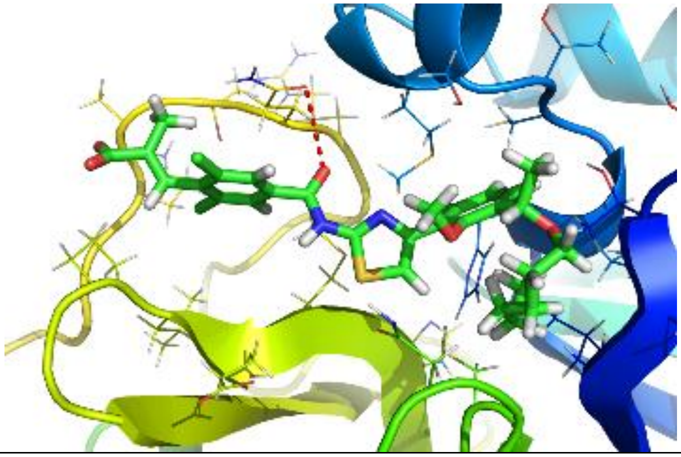	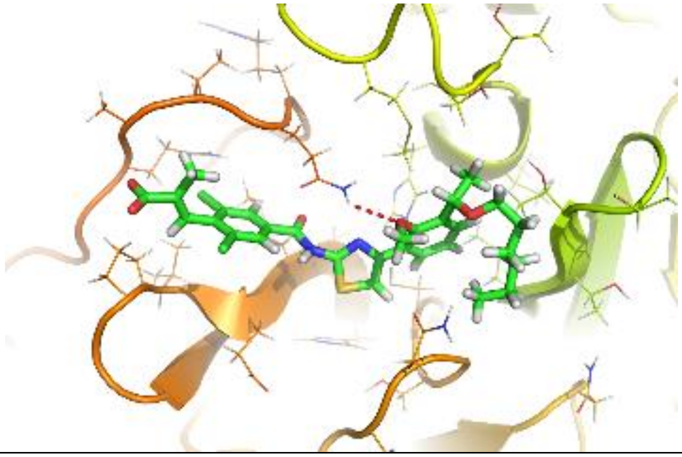
Mefloquine	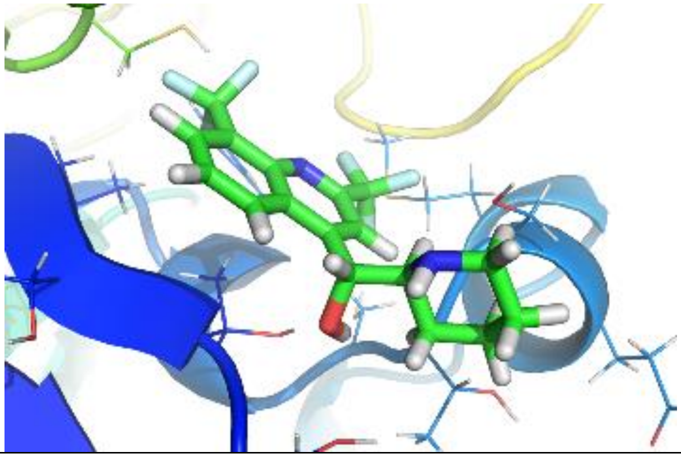	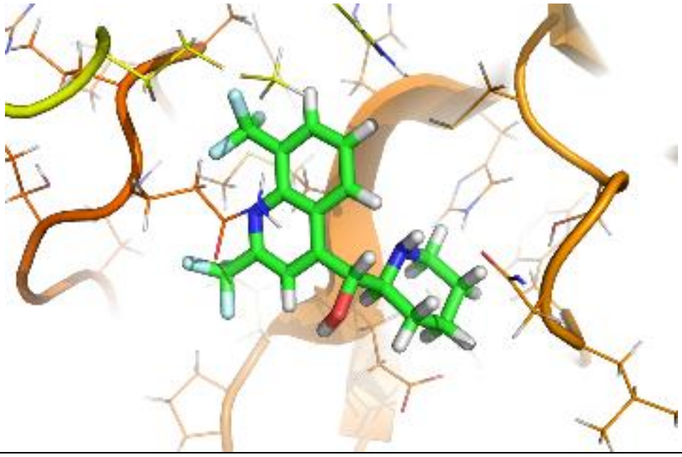

Mequitazine	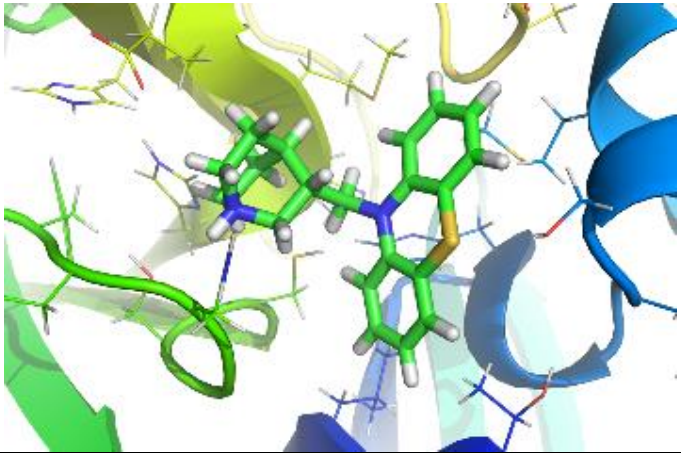	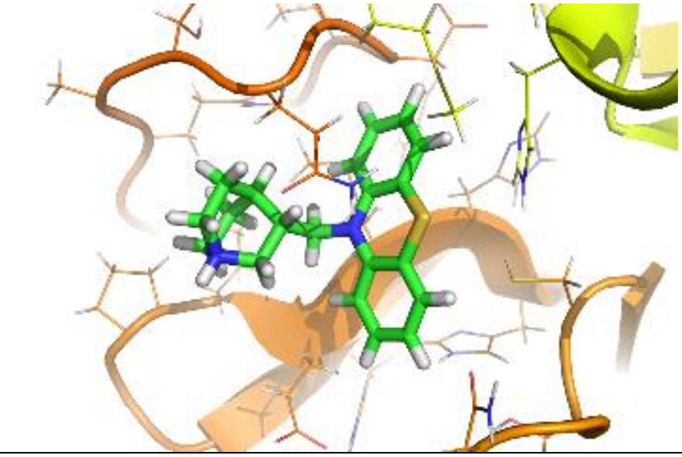
Osajin	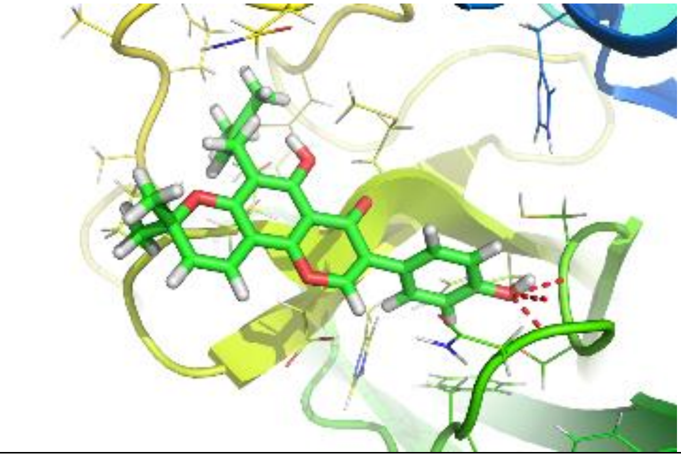	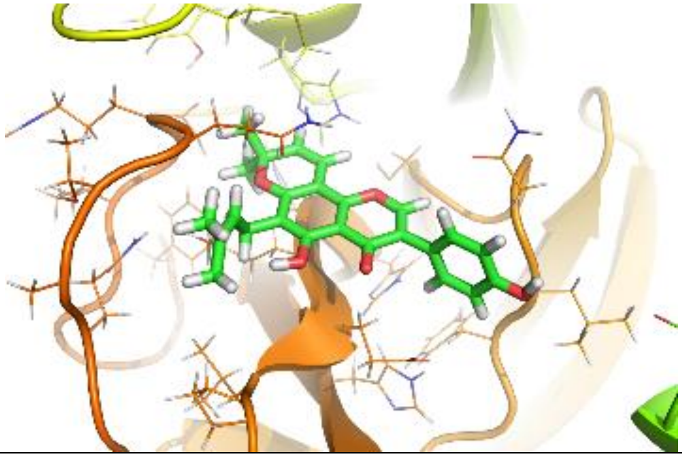

Oxyclozanide	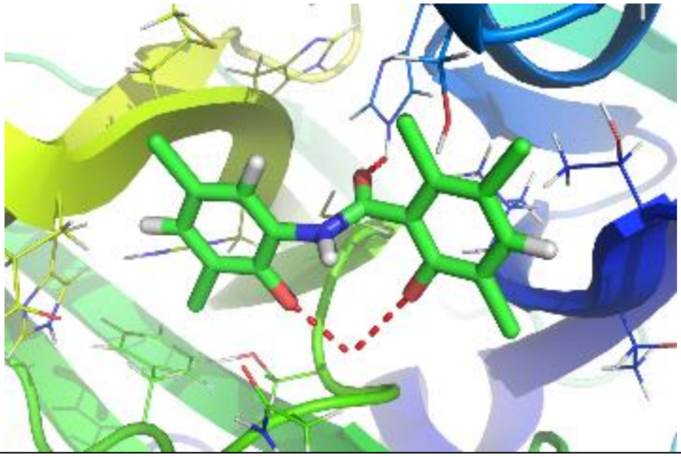	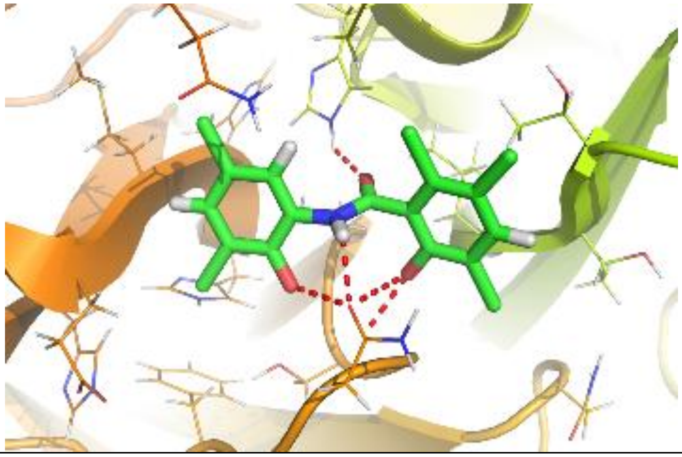
Penfluridol	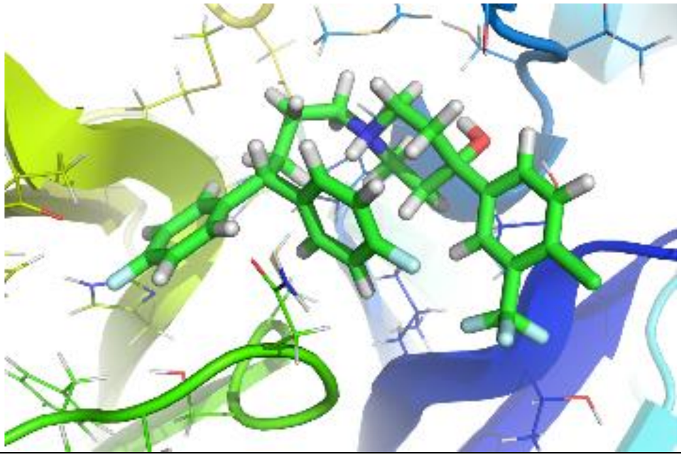	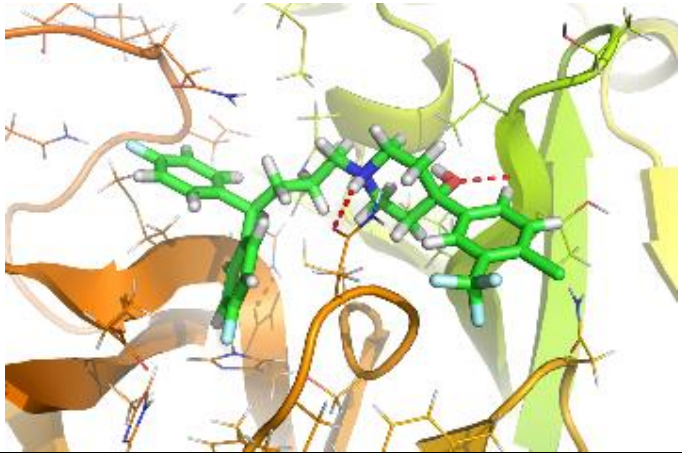

Phenazopyridine	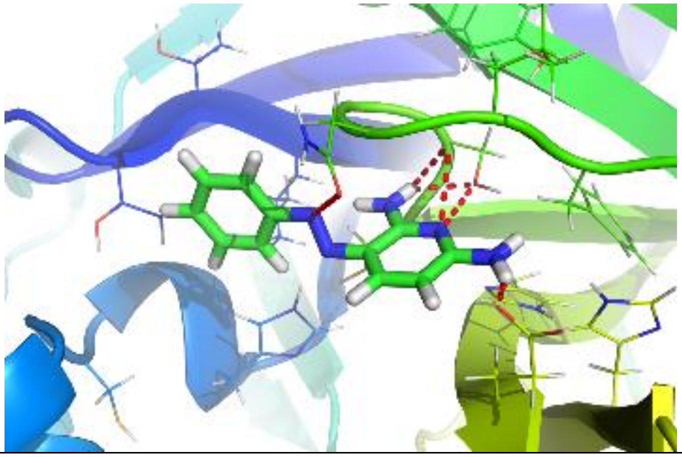	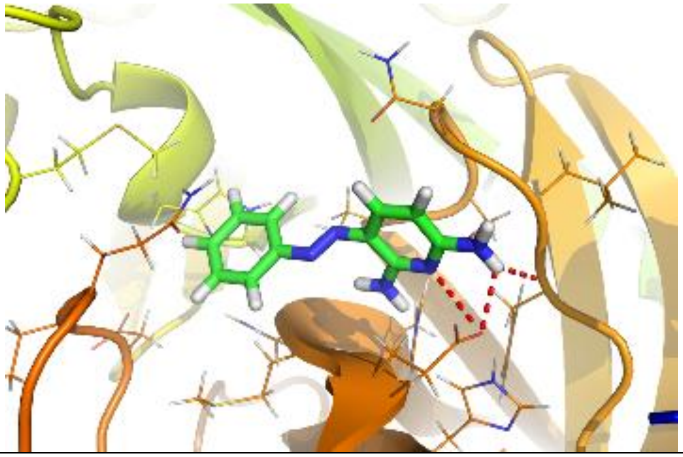
Proscillaridin	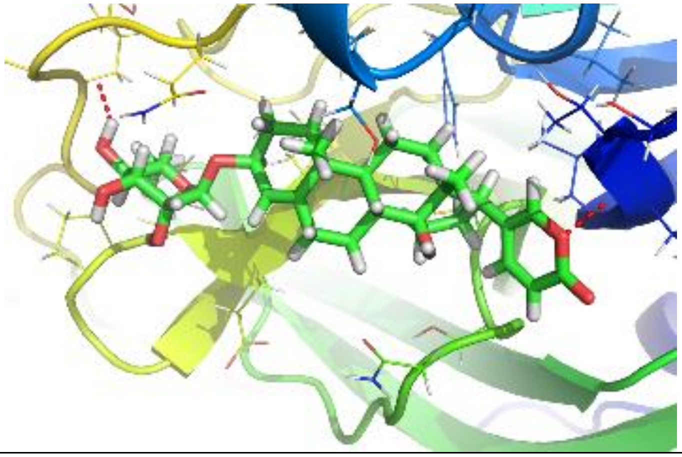	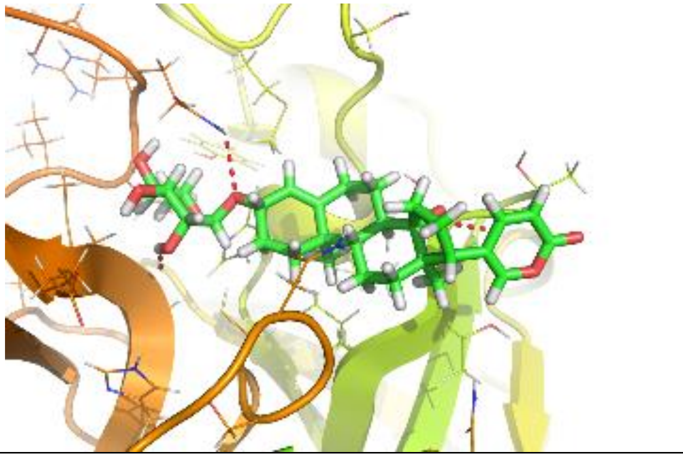

Tetrandrine	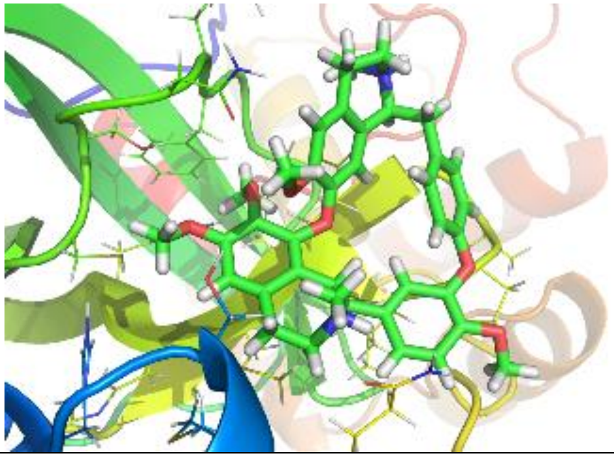	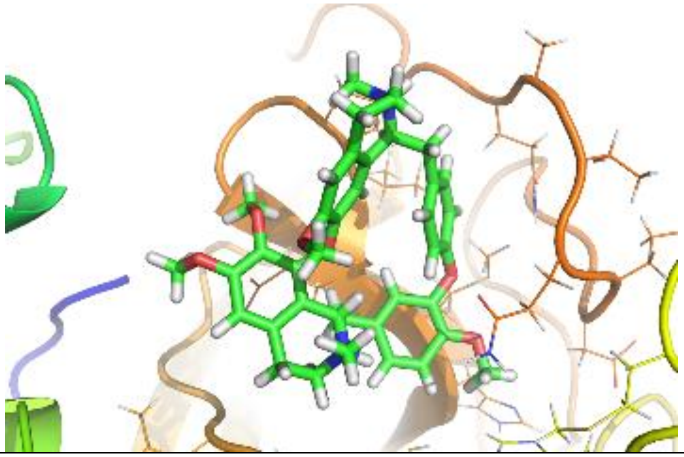

Table S6. The Pulling Force in Time Evolution.

Name	Monomer	Dimer
Bazedoxifene	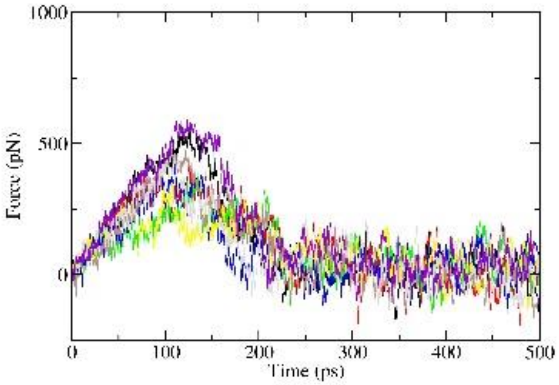	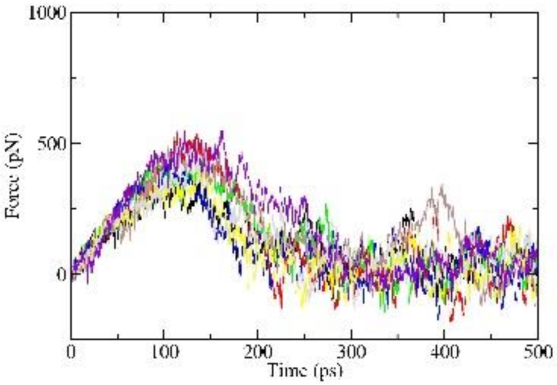
Cyclosporine	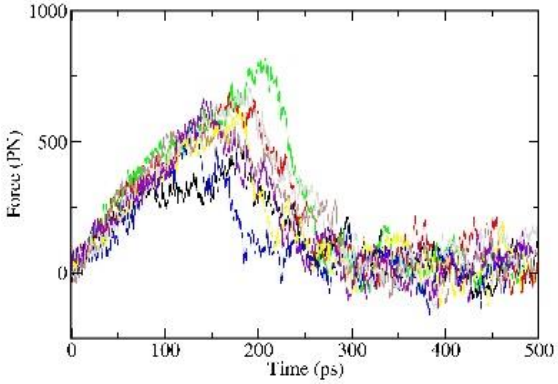	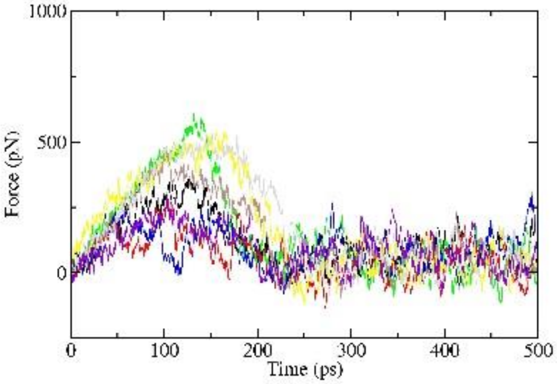

Digitoxin	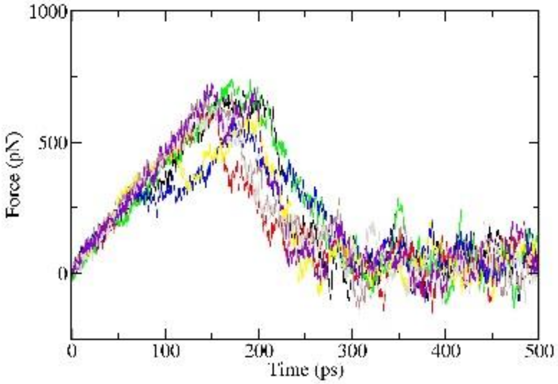	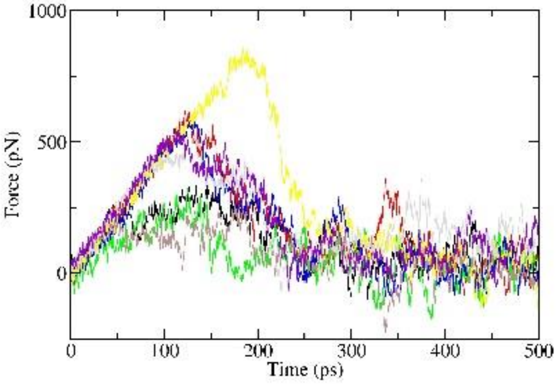
Digoxin	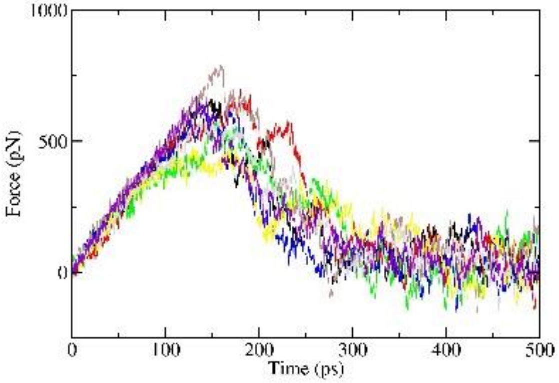	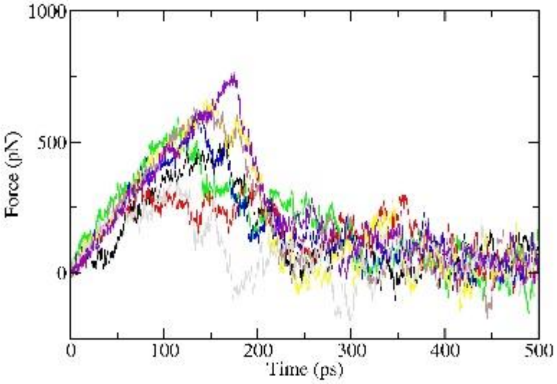

Dihydrogambogic Acid	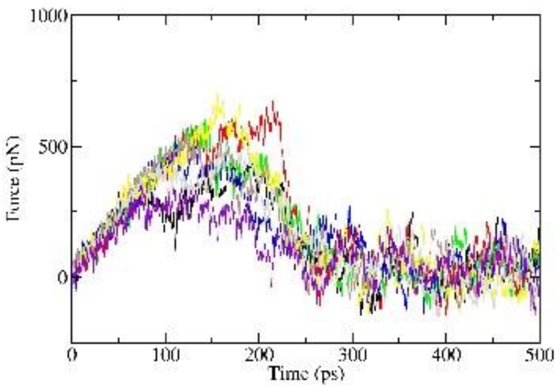	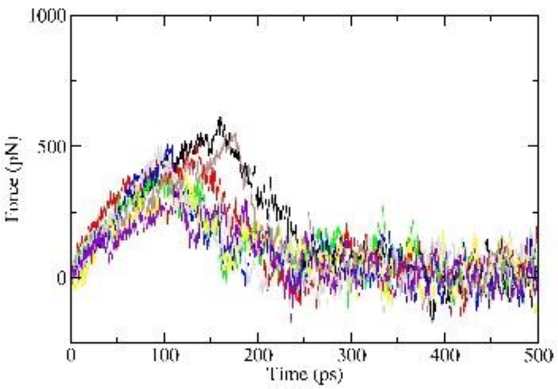
Ebastine	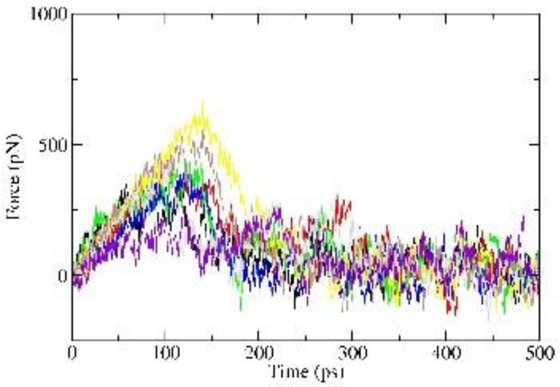	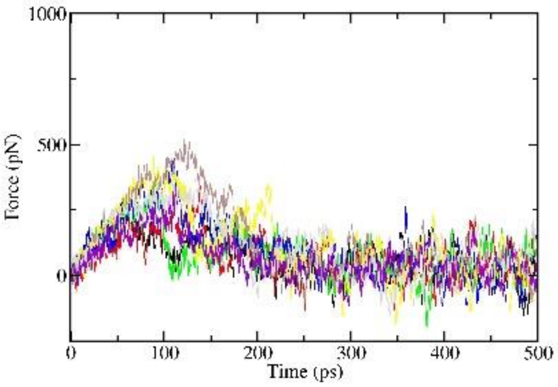

<table>
<tr>
<td>Favipiravir</td>
<td>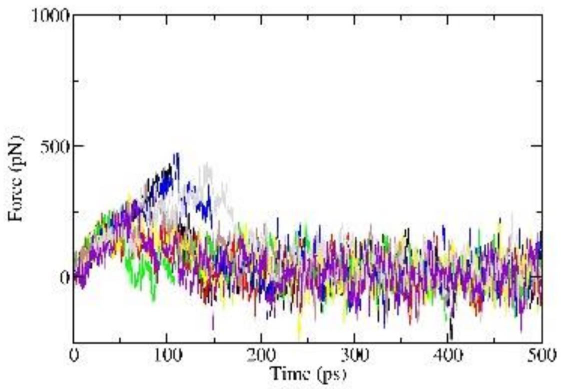
</td>
<td>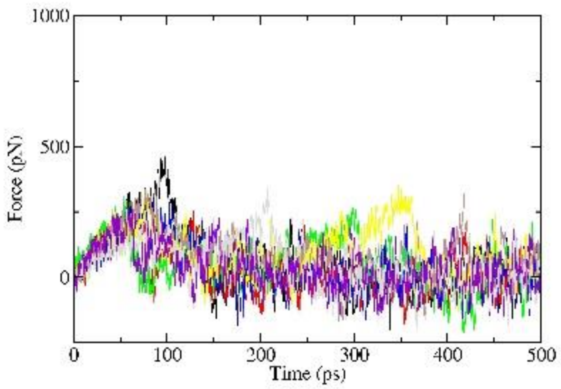
</td>
</tr>
<tr>
<td>Fluspirilene</td>
<td>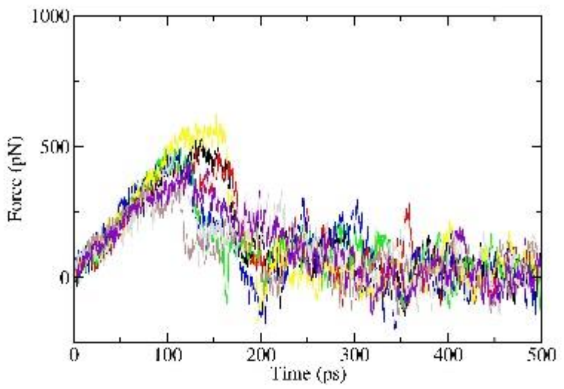
</td>
<td>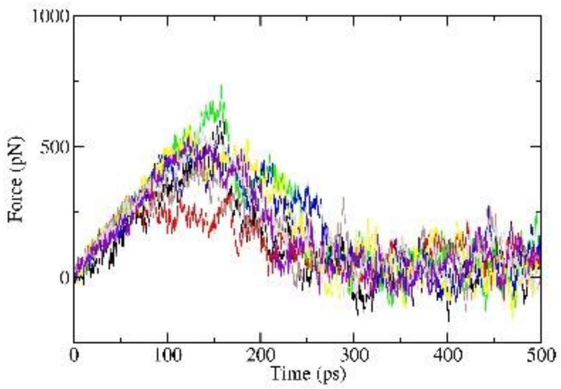
</td>
</tr>
</table>

Isoosajin	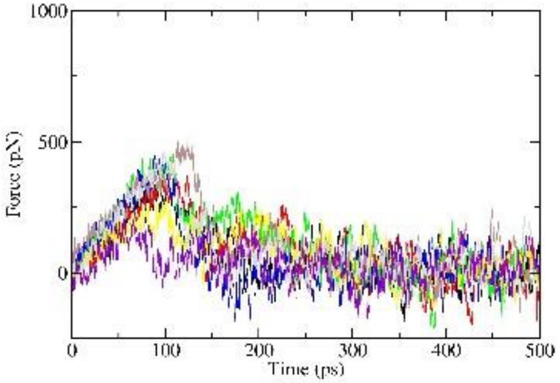	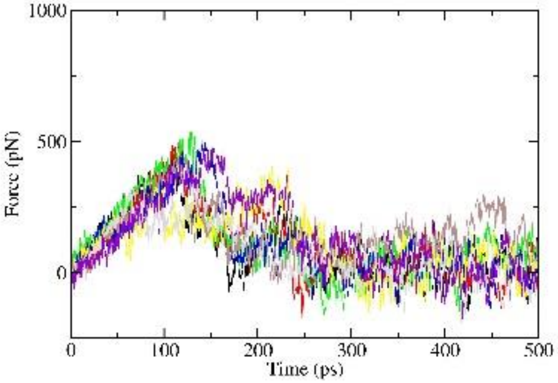
Ivacaftor	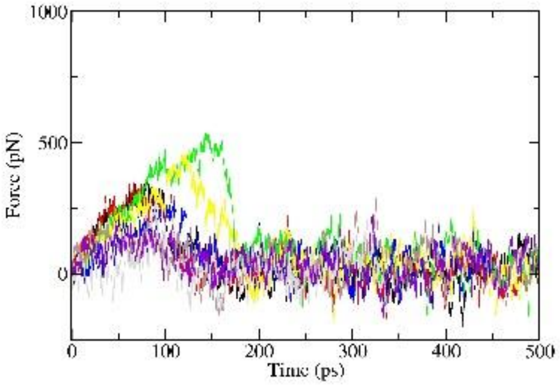	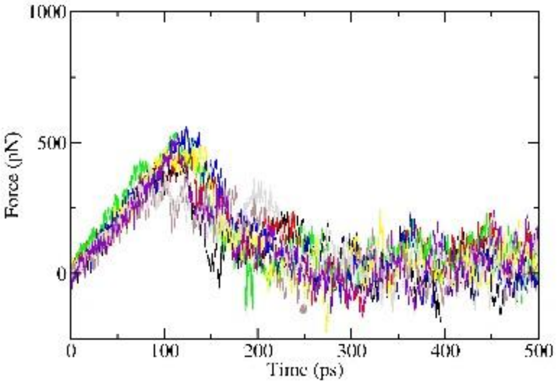

Lusutrombopag	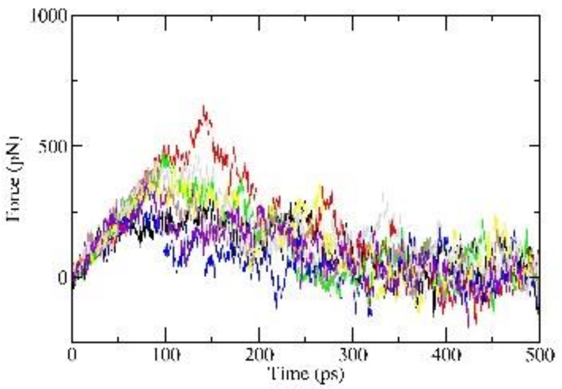	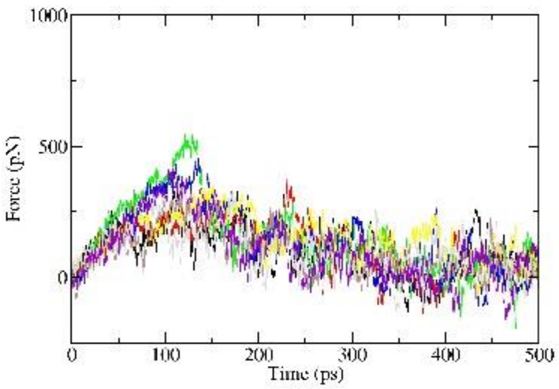
Mefloquine	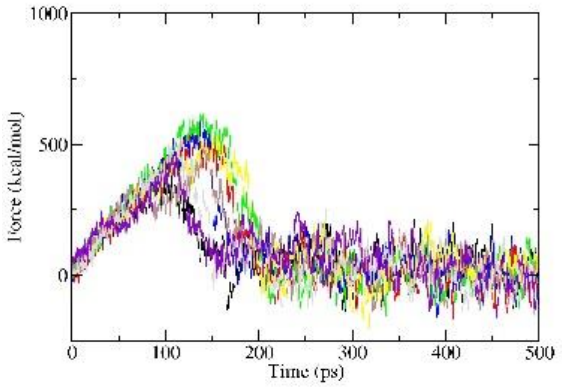	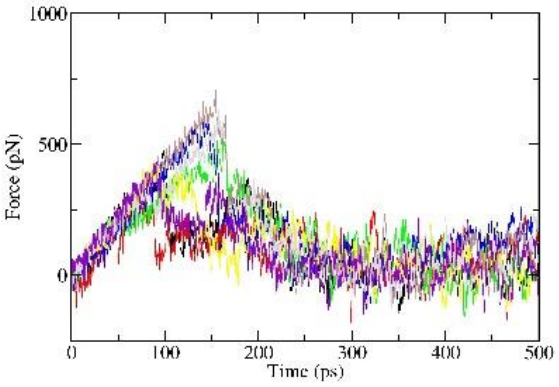

<table>
<tr><td>Mequitazine</td><td>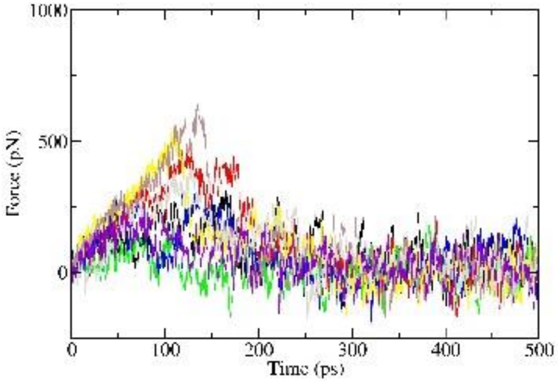
</td><td>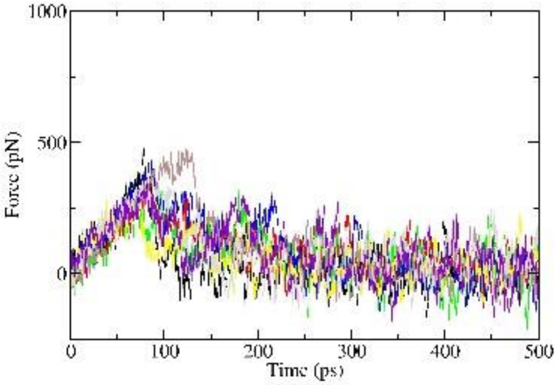
</td></tr>
<tr><td>Osajin</td><td>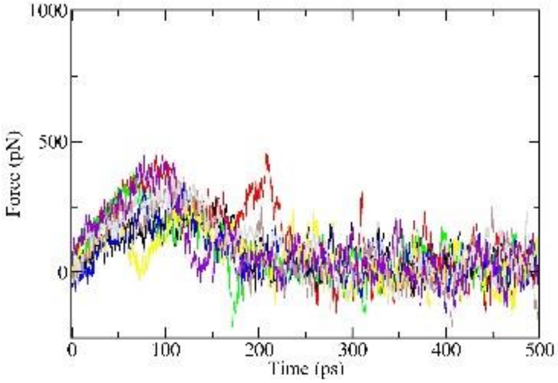
</td><td>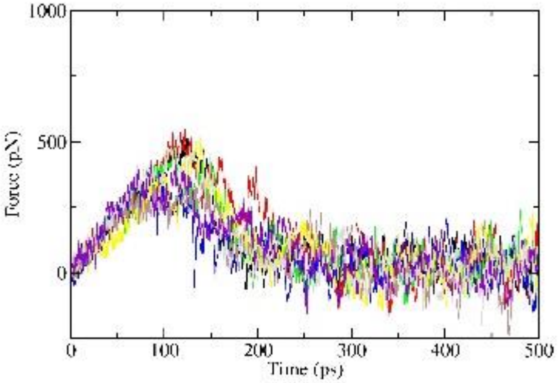
</td></tr>
</table>

Oxyclozanide	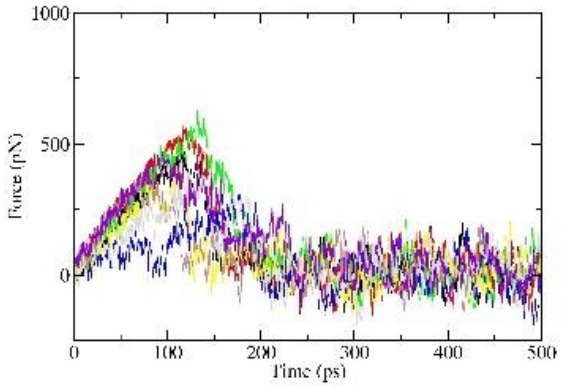	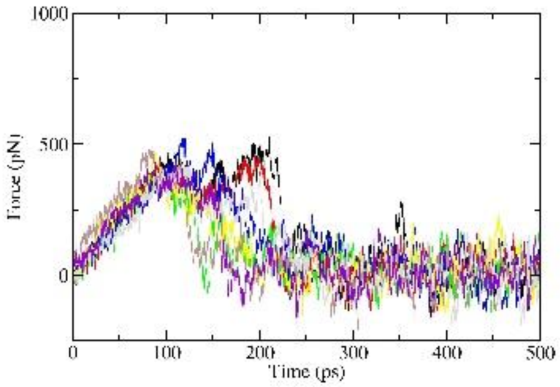
Penfluridol	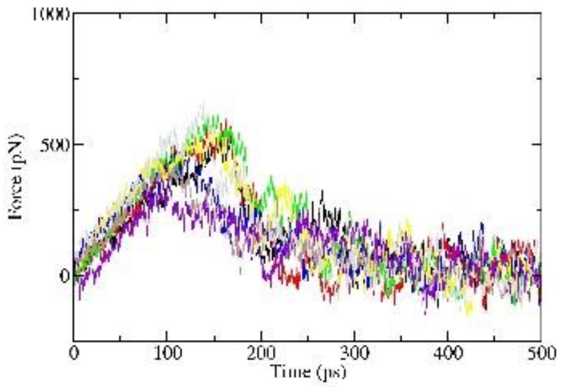	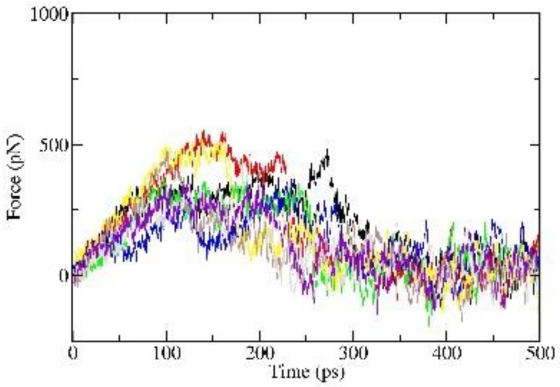

Phenazopyridine	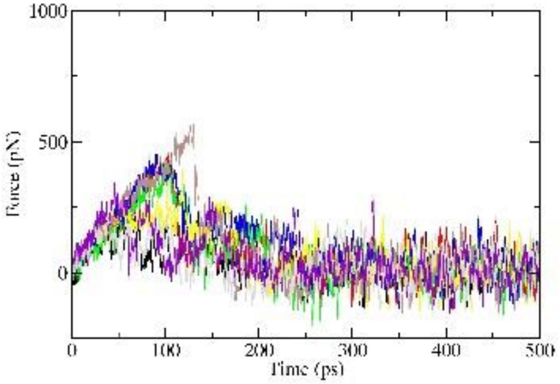	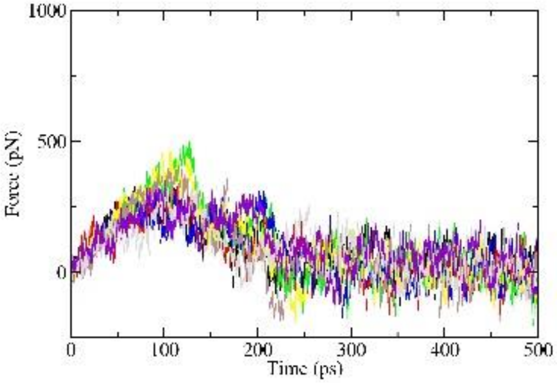
Proscillaridin	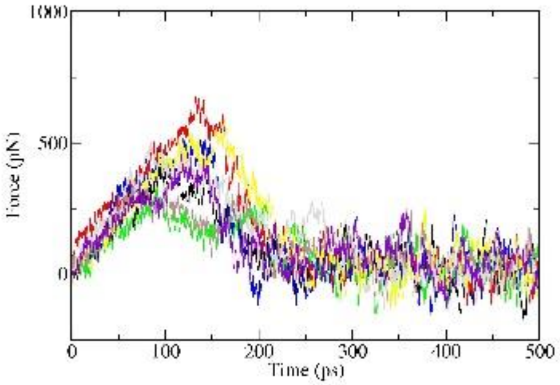	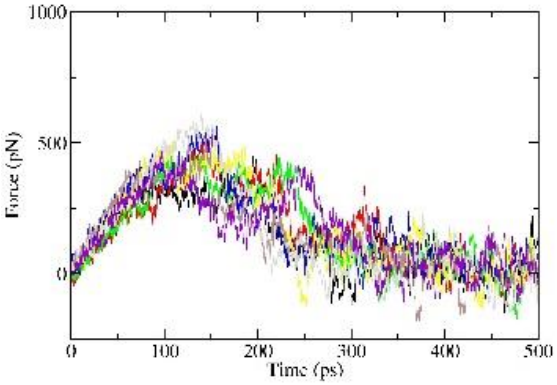

Tetrandrine	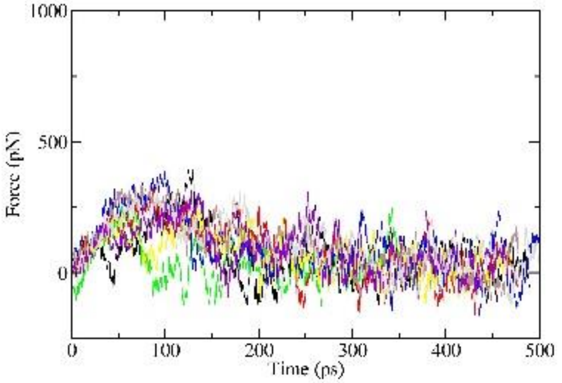	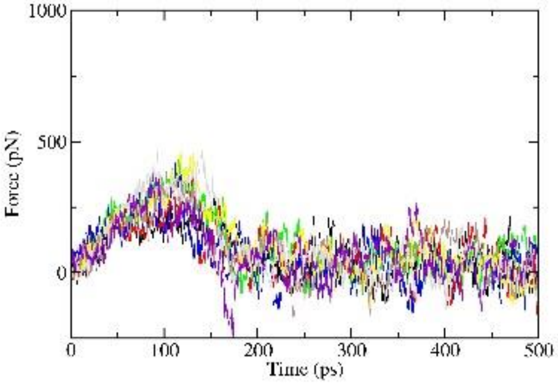

Table S7. The Pulling Work in Time Dependence over 8 Independent SMD Trajectories.

Name	Monomer	Dimer
Bazedoxifene	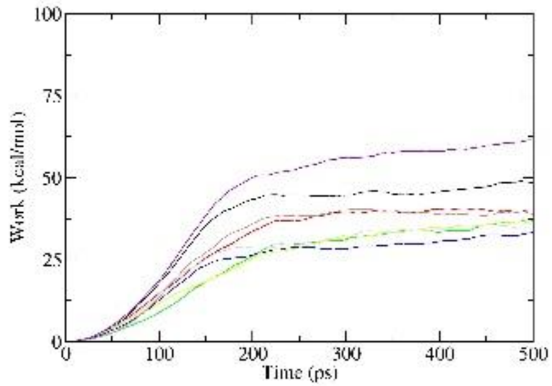	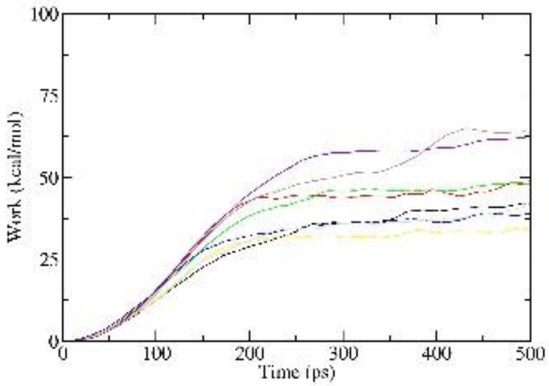
Cyclosporine	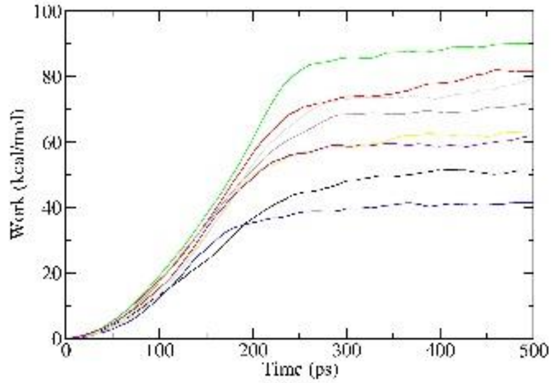	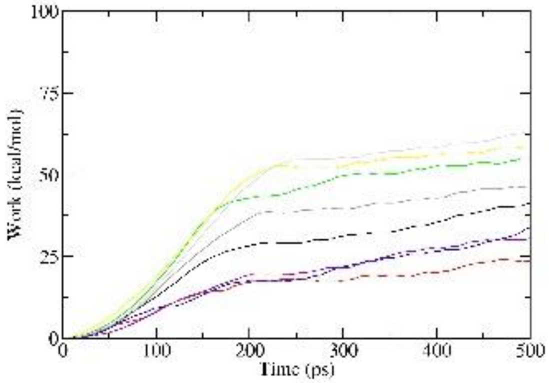

<table>
<tr><td>Digitoxin</td><td>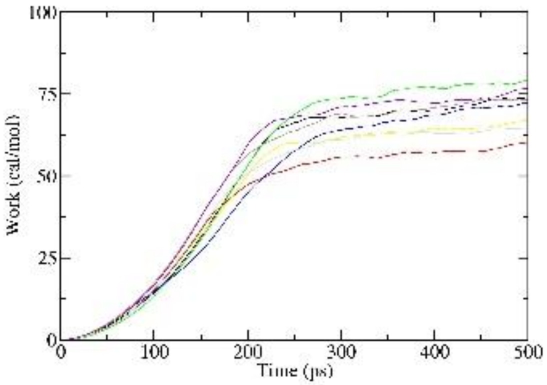
</td><td>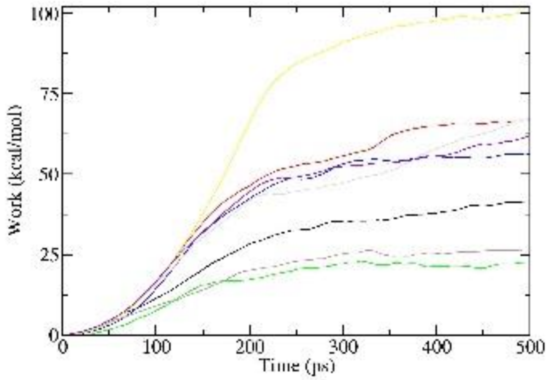
</td></tr>
<tr><td>Digoxin</td><td>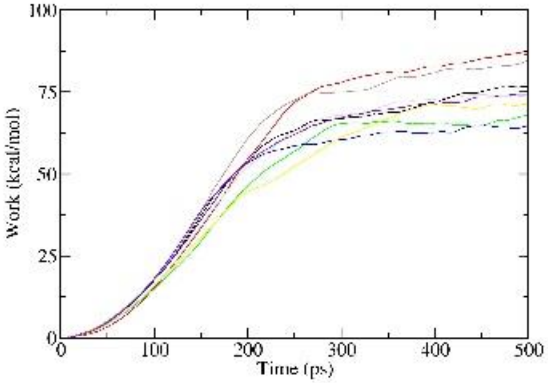
</td><td>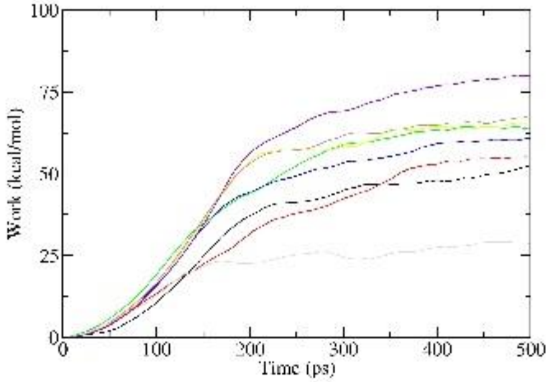
</td></tr>
</table>

Dihydrogambogic Acid	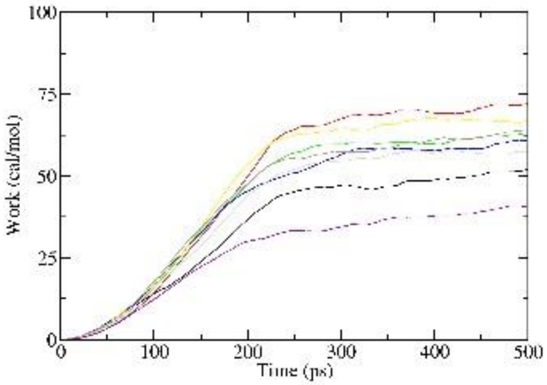	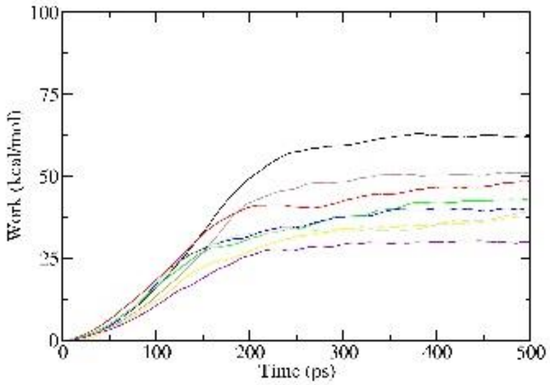
Ebastine	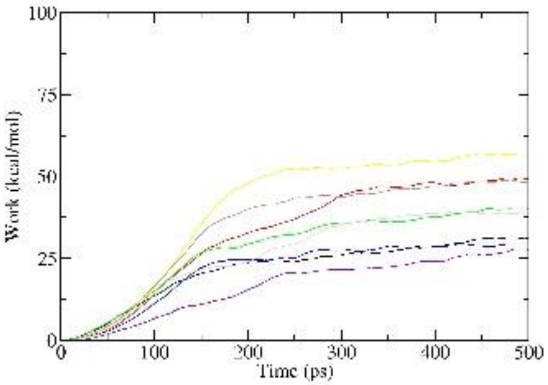	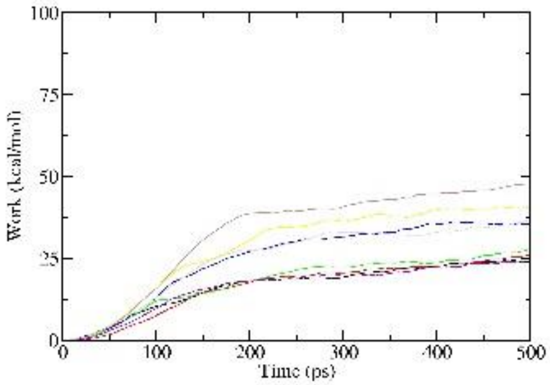

Favipiravir	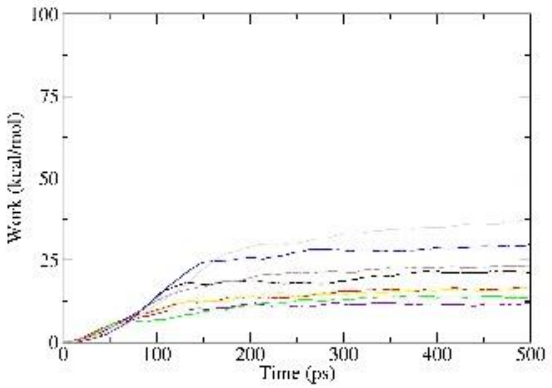	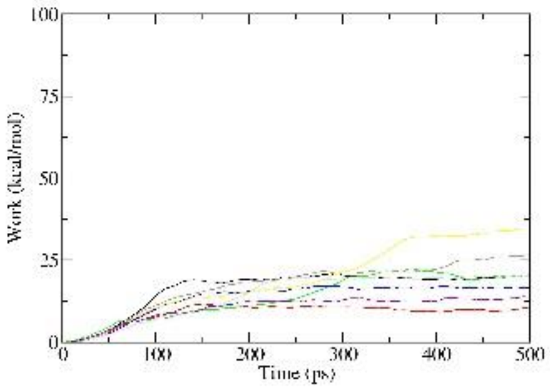
Fluspirilene	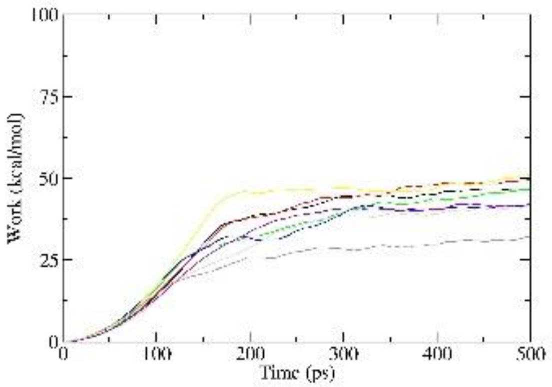	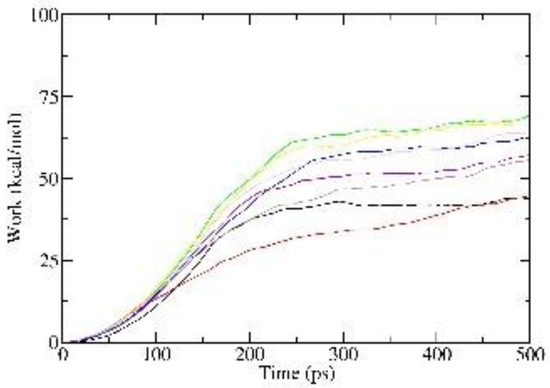

<table>
<tr><td>Isoosajin</td><td>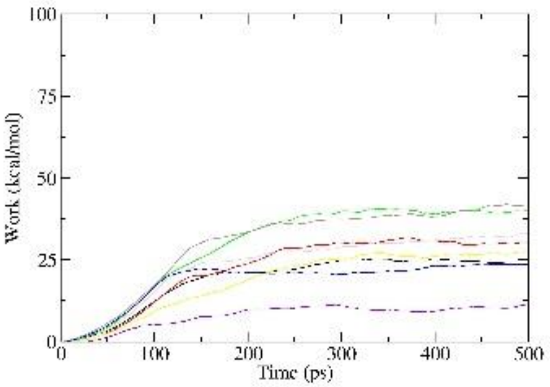
</td><td>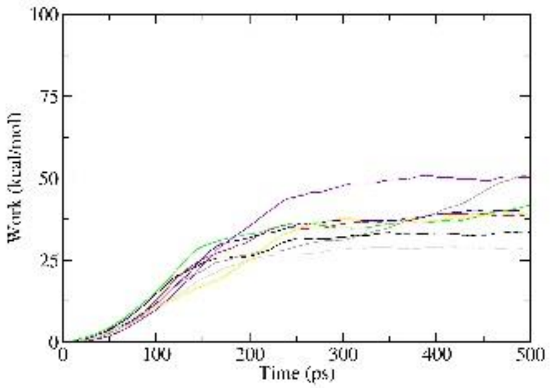
</td></tr>
<tr><td>Ivacaftor</td><td>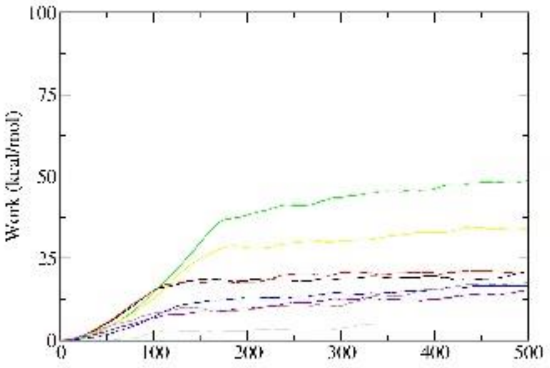
</td><td>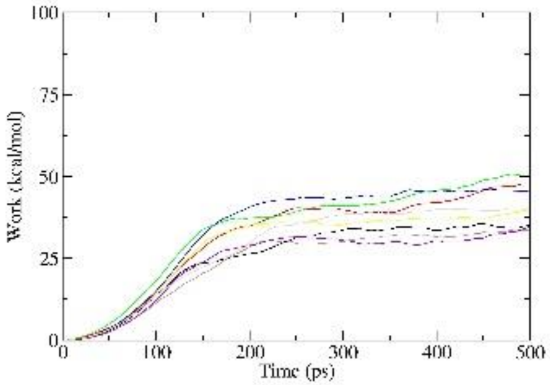
</td></tr>
</table>

Lusutrombopag	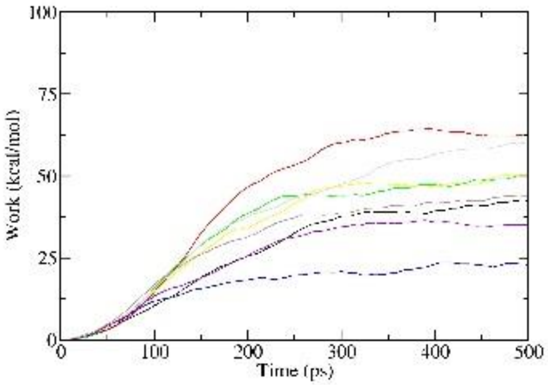	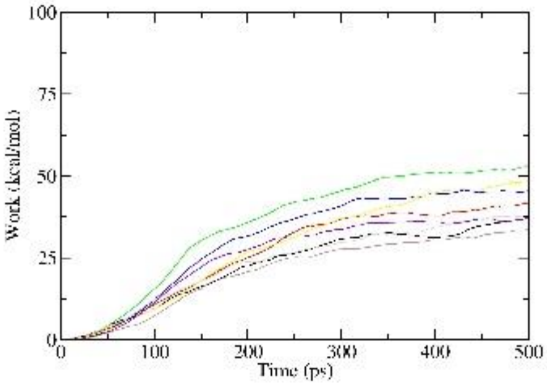
Mefloquine	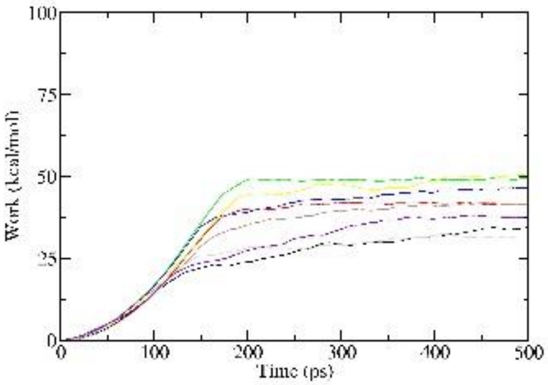	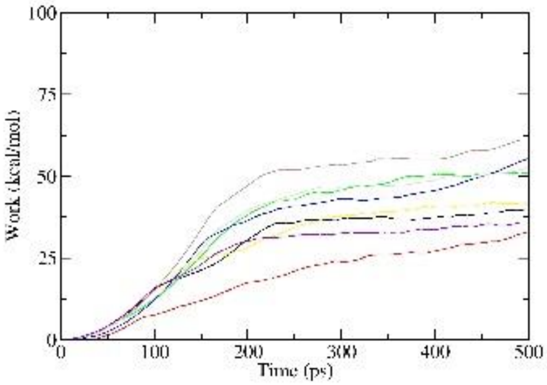

Mequitazine	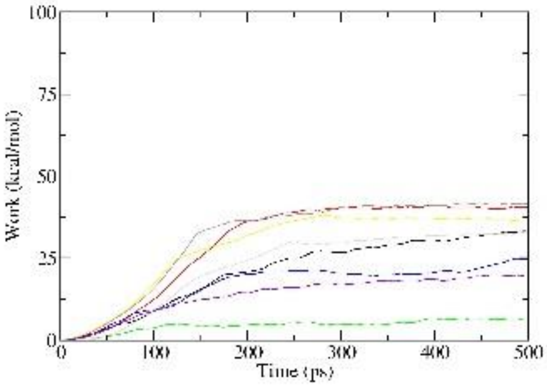	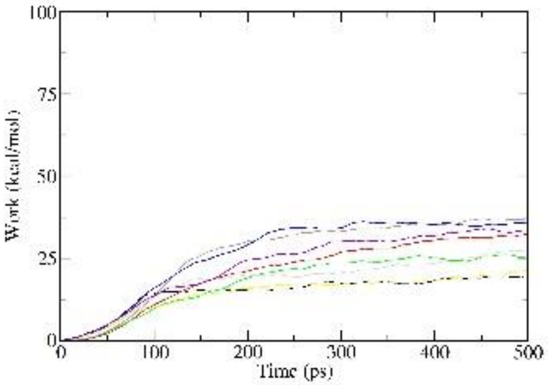
Osajin	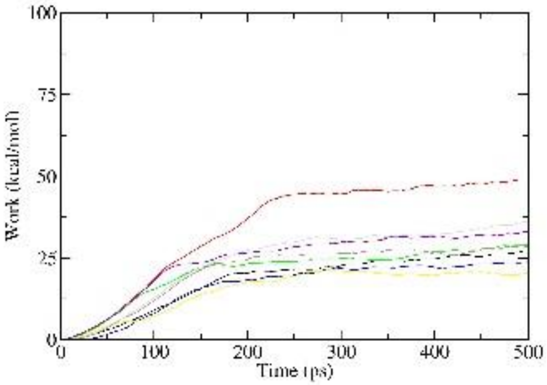	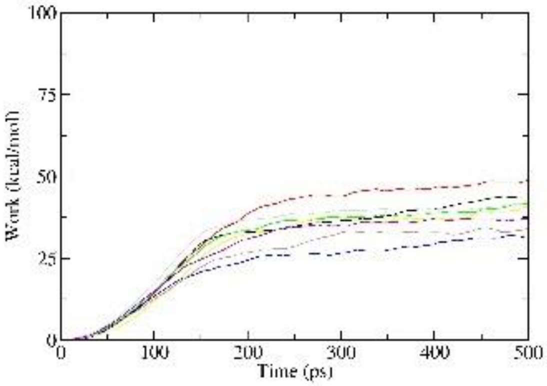

Oxyclozanide	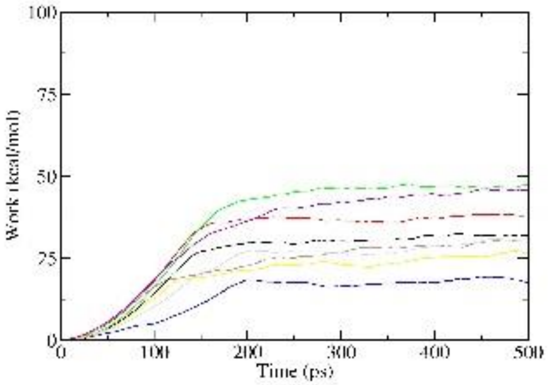	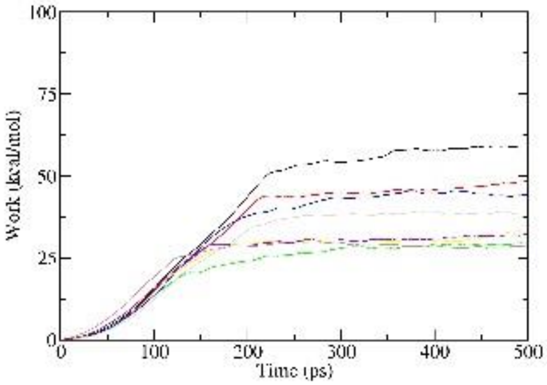
Penfluridol	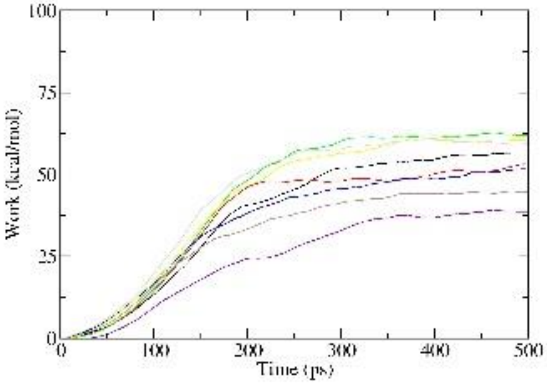	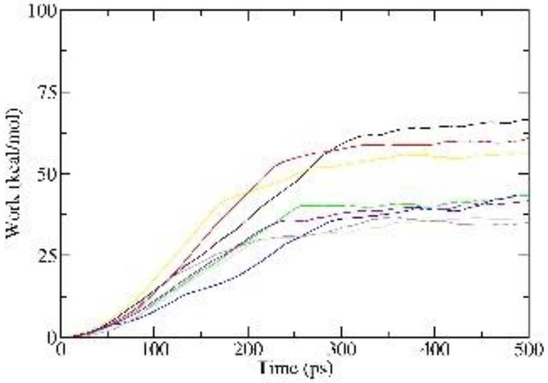

<table>
<tr>
<td>Phenazopyridine</td>
<td>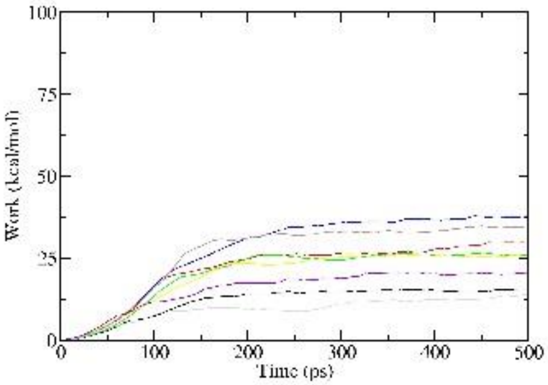
</td>
<td>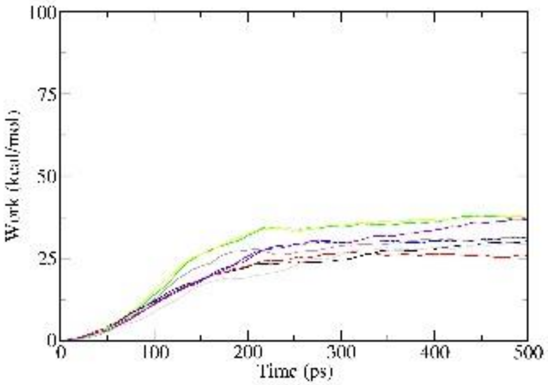
</td>
</tr>
<tr>
<td>Proscillaridin</td>
<td>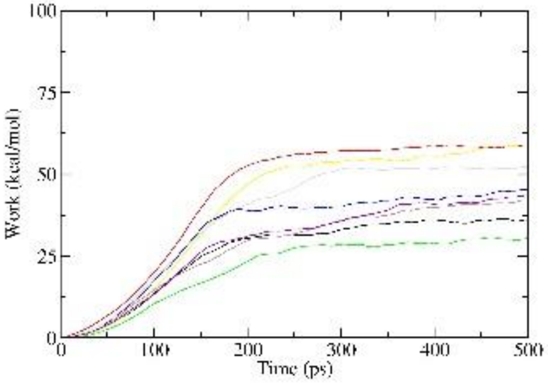
</td>
<td>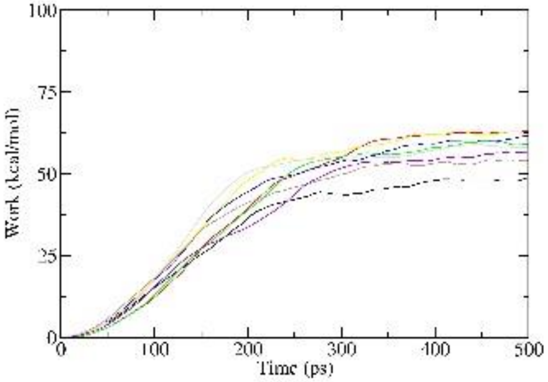
</td>
</tr>
</table>

Tetrandrine	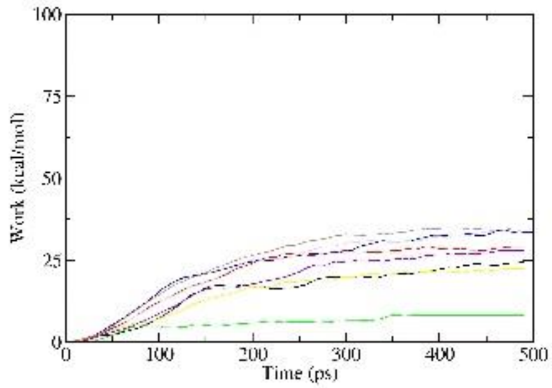	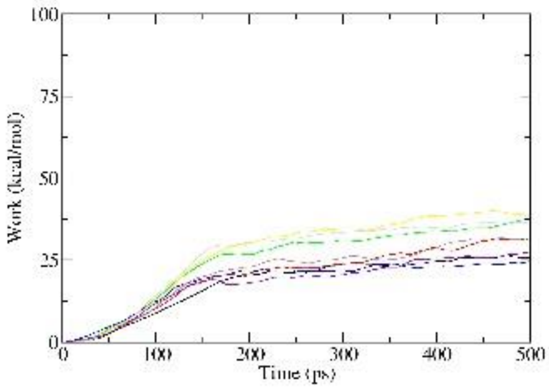

Reference:

1. Salentin, S.; Schreiber, S.; Haupt, V. J.; Adasme, M. F.; Schroeder, M., PLIP: fully automated protein–ligand interaction profiler. *Nucleic Acids Res.* **2015,** *43*, W443-W447.
